# LC-MS screening of poly- and perfluoroalkyl substances in contaminated soil by Kendrick mass analysis

**DOI:** 10.1007/s00216-019-02358-0

**Published:** 2020-01-09

**Authors:** Boris Bugsel, Christian Zwiener

**Affiliations:** Environmental Analytical Chemistry, Center for Applied Geoscience, University of Tübingen, Hölderlinstraße 12, 72074 Tübingen, Germany

**Keywords:** Poly- and perfluoroalkyl substances, High-resolution mass spectrometry, Screening, Kendrick mass, Soil, Contamination

## Abstract

The application of contaminated paper sludge on arable land in southwest Germany caused the occurrence of a broad range of poly- and perfluoroalkyl substances (PFASs) on soil. Recently, the dead-end transformation products (TPs) perfluorooctanoic acid and perfluorooctanesulfonic acid were detected in groundwater and drinking water. The precursors and other transformation products mostly remained unknown. Therefore, HRMS screening by Kendrick mass analysis and assignment of homologous series in combination with suspect screening were applied to identify original PFASs and their TPs in four different soil samples from sites where contaminated paper sludge was applied. In total, twelve compound classes comprising more than 61 PFASs could be fully or tentatively identified. The data reveal that contamination mainly originates from polyfluorinated dialkylated phosphate esters (from 4:2/6:2 to 12:2/14:2), *N*-ethyl perfluorooctane sulfonamide ethanol–based phosphate diesters (only C_8_/C_8_) and transformation products of these precursors. Contamination patterns can be attributed to PFASs used for paper impregnation and can vary slightly from site to site.

**Electronic supplementary material:**

The online version of this article (10.1007/s00216-019-02358-0) contains supplementary material, which is available to authorized users.

**Figure:**
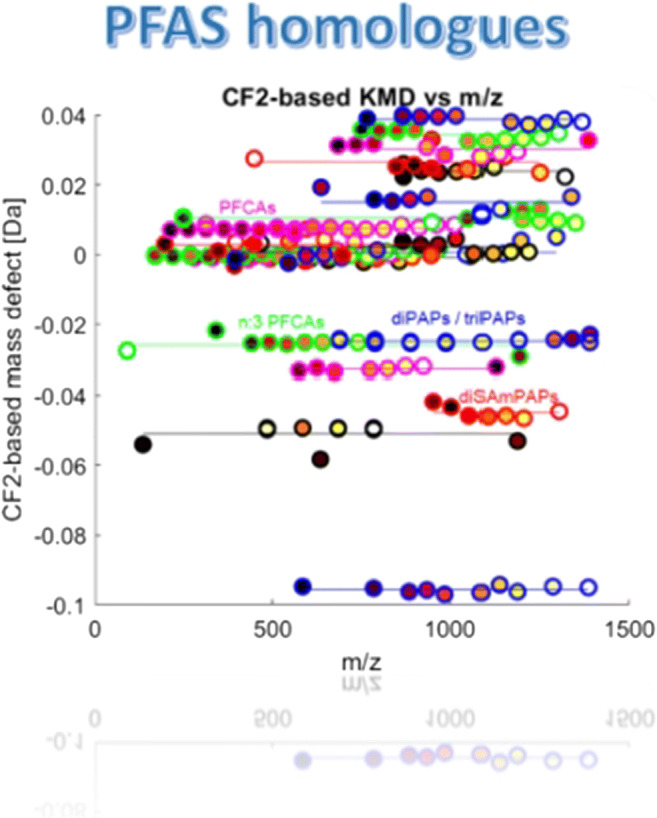
Graphical abstract

## Introduction

Poly- and perfluorinated alkylated substances (PFASs) are anthropogenic compounds with an increasing environmental significance [1]. They comprise a family of more than 4700 single compounds [2]. Due to their unique, both water- and grease-repelling properties, their fields of applications are textiles such as clothing and carpets [3], nonstick cookware [4], food packaging [5, 6], cosmetics [7], and firefighting foams [8, 9]. The widespread use of PFASs has led to a global distribution in biota [10], air [11], soil [12], and water where they were even proposed as novel tracers [13]. While the characteristics of PFASs render them desirable for the industry, their negative impacts on both environment and health are beyond question. Two of the best studied PFASs, perfluorooctanoic acid (PFOA) and perfluorooctanesulfonic acid (PFOS), accumulate in the liver, lung, and kidney of rats and are proposed to be a big threat to humans and biota [14, 15]. Their major manufacturer 3M has hence phased out the production of these two substances between 2000 and 2002 [1], yet other PFASs such as ammonium 2,3,3,3-tetrafluoro-2-(heptafluoropropoxy)-propanoate (GenX) have replaced these compounds [16]. Studies performed by Gomis et al. [17] and Sun et al. [18] even indicate a higher toxicity and a lower sorption on activated carbon for GenX than for PFOA, highlighting the importance of both legacy and novel PFASs.

Liquid chromatography coupled to mass spectrometry has earlier been used in the identification and determination of PFASs [5, 8, 19–22]. The use of high-resolution mass spectrometry (HRMS) reveals valuable accurate mass information which can be used in the identification of unknown contaminants. The nature of PFASs typically is their occurrence in multiple homologues, a beneficial characteristic in their identification. Coupling HRMS with homologous series (HS) detection has a variety of useful applications such as the detection of surfactants or polymers [23, 24] and PFASs [25, 26].

In summer 2013, a routine sampling has revealed PFAS contamination of a drinking water well near Rastatt (Baden-Württemberg, Germany) [27]. Further research showed that soil contamination on agricultural land has led to groundwater pollution. Compost mixed with paper sludge containing PFASs was applied to about 7.8 Mio m^2^ agricultural soil [28, 29]. A large variety of both anionic and non-ionic PFASs is known to be applied to food packaging paper and board in order to grant oil and water repellency [6]. Schaider et al. [30] found that 46% out of roughly 400 food contact papers showed detectable levels of fluorinated substances. Typically, these PFAS paper impregnation agents can contain multiple alkyl chains and functional groups [6]. Lab experiments revealed that the original PFAS paper impregnation products (precursors) are slowly degrading to short single-chain perfluoroalkyl acids (PFAAs) such as perfluorocarboxylic acids (PFCAs) and perfluorosulfonic acids (PFSAs) [31, 32]. The transformation products (TPs) are more mobile, migrate to groundwater, and can be taken up by plants [33, 34]. To evaluate the exposure of the population which received contaminated drinking water, blood tests were conducted. Results of the contaminated area showed increased blood levels of PFASs of the exposed people (e.g., 15.7 μg/L PFOA) compared to controls (1.7 μg/L PFOA) [35]. In other regions, PFOA blood concentrations of exposed people are also about 10-fold higher than those of control population [36].

In the present case study, information on the identity and amount of technical products used and discharged is very limited. These precursors are typically not included in target methods. The aim of the present work hence is the characterization and identification of the original contamination and its TPs. Here, we used a non-target approach with LC-high-resolution mass spectrometry and Kendrick mass analysis.

## Materials and methods

### Chemicals and reagents

Optima LC-MS grade methanol (MeOH), ammonium acetate (NH_4_Ac), and water were purchased from Fisher Scientific. Authentic reference standards of sodium (1*H*,1*H*,2*H*,2*H*-perfluorooctyl-1*H*,1*H*,2*H*,2*H*-perfluorodecyl)phosphate (6:2/8:2 diPAP), *N*-ethyl perfluorooctane sulfonamide ethanol–based phosphate diester (diSAmPAP), 2*H*-perfluoro-2-decenoic acid (8:2 FTUCA), *N*-ethylperfluoro-1-octanesulfonamidoacetic acid (EtFOSAA), sodium perfluoro-1-octanesulfonate (PFOS), 3-perfluoroheptyl propanoic acid (7:3 PFCA), 2*H*-perfluoro-2-decenoic acid (8:2 PFCA), and sodium 1*H*,1*H*,2*H*,2*H*-perfluorooctane sulfonate (6:2 FTSA) were obtained from Wellington Laboratories, Inc. (Guelph, Ontario, Canada). Tris(1*H*,1*H*,2*H*,2*H*-perfluorohexyl) phosphate (6:2 polyfluorinated trialkylated phosphate ester, triPAP) was purchased from Chiron AS (Trondheim, Norway). Bis(perfluorohexyl)phosphonic acid (perfluorinated phosphonic acid, PFPIA) was purchased from Toronto Research Chemicals (North York, Ontario, Canada). PFOA was obtained from ABCR (Karlsruhe, Germany). Perfluorooctanesulfonamide (FOSA) was obtained from SynQuest Laboratories (Alachua, FL, USA).

### Sample collection and preparation

The homogenized, freeze-dried soil samples from the contaminated area in Germany were collected in 2017 and provided by the Landwirtschaftliches Technologiezentrum Augustenberg (LTZ) (Karlsruhe, Germany). Sample 1 originated from Hügelsheim and samples 2, 3, and 4 from the area around Mannheim. Five grams of the soil samples was weighed in a 50-mL polypropylene (PP) tube, and 10 mL MeOH was added. The mixture was thoroughly vortexed for 2 min, sonicated for 10 min, and then put on a horizontal shaker for 24 h. After centrifuging (10 min, 4000 relative centrifugal force (rcf)), the supernatant was transferred to a 20-mL PP vessel using a glass pipette. The extraction was repeated with 10 mL MeOH as described. The supernatants were combined, heated to 40 °C, and evaporated to less than 1 mL with a gentle stream of nitrogen. Pure MeOH was used to adjust the volume to 1 mL. The concentrate was transferred into a PP vial and centrifuged (10 min, 4000 rcf) prior to analysis.

### Instrumental analysis

Samples were analyzed by LC-MS using a 1290 HPLC (Agilent Technologies, Waldbronn, Germany) coupled to a 6550 QTOF mass spectrometer (Agilent Technologies, Santa Clara, USA). An Agilent C18 column (Poroshell 120 EC-C18, 2.1 mm × 100 mm, particle size 2.7 μm) was used to separate the analytes, and the flow rate was set to 0.4 mL/min and the temperature to 40 °C. Eluent A (95:5 H_2_O/MeOH) and eluent B (5:95 H_2_O/MeOH), both with 2 mM NH_4_Ac, were used for gradient elution. The gradient started with 25% B, followed by a linear increase to 85% B within 2 min and to 100% B within 2.5 min, and kept isocratic for another 12 min. Initial conditions were reset at 12.1 min with a subsequent equilibration time of 15 min. The injection volume of the sample was 20 μL. The electrospray ionization (ESI) source, equipped with the Agilent Jet Stream technology, was operated in negative ionization mode. The MS measurements were performed in scan mode for screening and in both targeted MS/MS mode and all-ion fragmentation (AIF) for further structural information. AIF measurements were performed at collision energies of 0 eV, 20 eV, and 40 eV. Instrumental parameters for MS/MS measurements are given in Electronic Supplementary Material (ESM) 1 (section targeted MS/MS measurements).

To validate the extraction method, targeted MS/MS measurements were performed with uncontaminated soil spiked with 10 ng of several PFASs (diSAmPAP, 8:2 PFCA, EtFOSAA, 6:2 FTSA) each. Original uncontaminated and spiked soil samples were extracted in the same way as the contaminated samples 1–4. The results are shown in the ESM (Figs. S1.42 to S1.45).

### Data analysis and HS detection

The recorded data were processed using the molecular feature extraction (MFE) algorithm of the MassHunter software (versions B.07.00 and 10.0; Agilent Technologies). Features were narrowed down to mass defects between − 0.25 and + 0.1 Da as well as an intensity of at least 1000 counts. To validate the mass defect filtering, we used the curated Organisation for Economic Co-operation and Development (OECD) PFAS list (list of acronym PFASOECDNA, available from the EPA dashboard) and calculated the mass defect of all entries. Out of a total of 3213 PFASs, 2982 substances had a mass defect between − 0.25 and + 0.1, showing that an application of this filter covers 92.8% of all PFASs within this database. The resulting compounds after filtering are hydrocarbon compounds with chemical formulae containing more or less elements with negative mass defects (e.g., F, Cl, Br, I, O, P, S).

The selected features were exported as a CSV file which could then be imported to Matlab (MathWorks, Natick, USA) to automatically be processed by FindSeries, a code which was written in-house for Kendrick mass defect (KMD) analysis of PFAS. As proposed in 1963 by Edward Kendrick [37], the KMD was calculated according to Eqs. (1) and (2). By normalizing the observed mass (OM) of a compound by the integer mass of the repeating unit (e.g., 50:49.9968 in the case of CF_2_), all homologue compounds with the same core structure but varying numbers of the repeating unit reveal the same KMD. For example, perfluoroheptanoic acid (PFHpA), PFOA, and perfluorononanoic acid (PFNA) have the exact masses 363.9769, 413.9737, and 463.9705, respectively. They all bear a carboxylic acid group as a common structural moiety and are only distinguished by the number of the repeating units (CF_2_ with an exact mass of 49.9968). For example, the KMD for PFHpA is − 1.96 × 10^−4^ (Eq. 3) and exactly the same number for PFOA and PFNA or even larger PFCAs like perfluorohexadecanoic acid (C_16_HF_31_O_2_).

Detected HS are further visualized by plotting the KMD vs. m/z where compounds from the same compound class align horizontally.

1 $$ \mathrm{KM}=\mathrm{OM}\bullet \frac{\mathrm{nominal}\ \mathrm{mass}\ \left(\mathrm{repeating}\ \mathrm{unit}\right)}{\mathrm{exact}\ \mathrm{mass}\ \left(\mathrm{repeating}\ \mathrm{unit}\right)} $$

2 $$ \mathrm{KMD}=\mathrm{nominal}\ \mathrm{KM}-\mathrm{exact}\ \mathrm{KM} $$

3 $$ \mathrm{KMD}=364-363.9769\bullet \frac{50}{49.9968}=1.96\bullet {10}^{-4} $$

where KM is the Kendrick mass, OM is the accurate observed mass of the compound, and KMD is the Kendrick mass defect.

In step 1, FindSeries further compares the measured accurate masses with a database containing exact masses of known PFASs (curated OECD PFAS list, available online at EPA dashboard) [38] using a tolerance of 3 mDa. Matching masses are exported, including the respective name, yielding a list of potentially occurring PFASs in the sample. In step 2, FindSeries performs a Kendrick mass analysis for different repeating units such as –CF_2_–, –CF_2_O–, or –C_2_F_4_O– using a tolerance of 5 mDa. This higher tolerance is chosen in step 2 because unlike in step 1, there is no reference mass value from a database, and all members from one HS need to deviate less than the applied tolerance from one another. The resulting HS are subsequently validated by a manual inspection of the chromatographic peaks (Gaussian peak shape, signal-to-noise ratio > 10) and a systematic retention time (RT) shift. The RT difference between the first members of a HS defines a RT shift, and the following homologues need to be in the same range. With increasing mass, a decreasing retention time shift has to be observed due to the increasing elution strength of the LC gradient. For example, four consecutive members of a HS with RT 3 min, 4 min, 4.1 min, and 6 min would be discarded (vastly different time shifts with no clear trend). In turn, four consecutive members of a HS with RT 3 min, 3.6 min, 4.1 min, and 4.5 min would be considered for further evaluation (plausible RT shift trend).

If available, one authentic standard per HS was purchased and used for confirmation of the compound identity. According to the scheme of identification confidence from Schymanski et al. [39], detected PFASs were classified by defined confidence levels. For level 1, the structure was confirmed by an authentic standard based on accurate mass and RT. Level 2a was used for members of a HS if one candidate was successfully confirmed by a reference standard and the other candidates exhibited the previously mentioned systematic mass and RT shifts. For level 2b, members of a HS with a systematic RT shift were detected and structures were validated with fragmentation patterns. Single features not belonging to a HS were classified as level 3, and compound identifications were tentatively proposed based on accurate mass and matching with the OECD database.

## Results and discussion

Highly contaminated soil samples were analyzed for PFAS precursors and TPs by LC-QTOF-MS. The procedure of the data evaluation is shown in detail for sample 1. Further results for samples 2, 3, and 4 are presented subsequently using the same work flow. For readability, only integer masses are shown in the result tables. The corresponding accurate masses are shown in Tables S1–S6 (see ESM).

Accurate mass scans showed in total 4837 features in the contaminated soil sample 1, 1940 of those had negative mass defects in the range between − 0.25 and + 0.1 Da, which typically characterize compounds with a high number of elements with negative mass defects, such as fluorine, chlorine, bromine, oxygen, phosphorus, or sulfur, and with a low number of hydrogen atoms with a positive mass defect. The mass defect filter considerably reduced the dataset, even though not all features with negative mass defects can be attributed to highly fluorinated compounds. Accurate mass data have been evaluated by Kendrick mass analysis for compounds characterized by HS with CF_2_, CF_2_O, and C_2_F_4_O repeating units.

### Data evaluation of sample 1

**Fig. 1:**
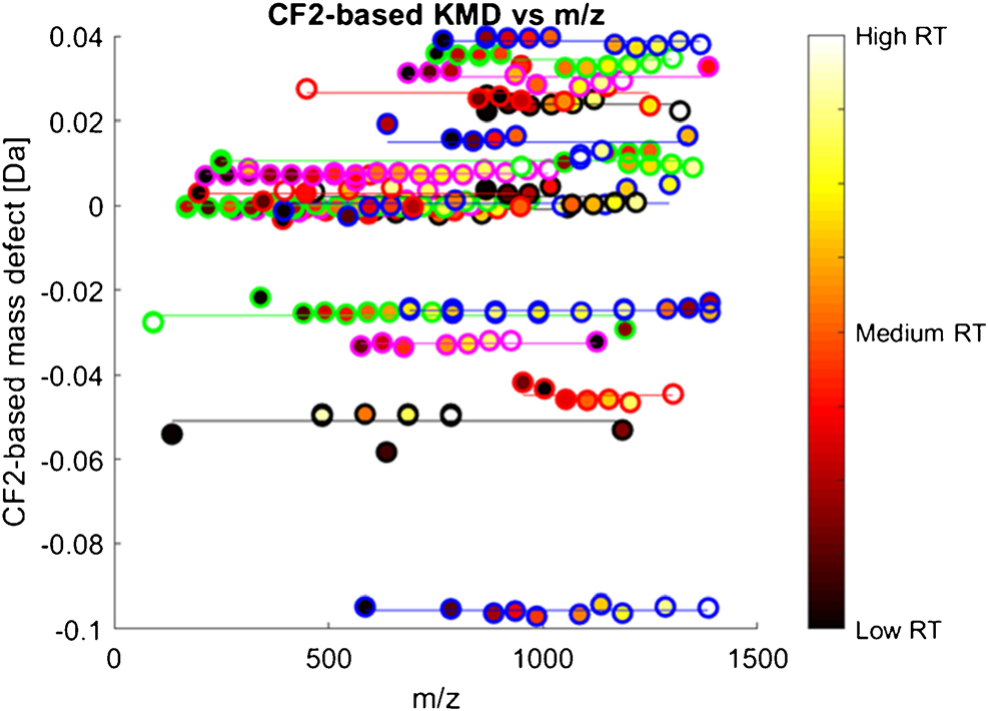
Kendrick mass defect for CF_2_ repeating units vs. mass-to-charge ratio. Only selected HS with at least 7 homologues are shown. Each feature is represented by a circle in different colors to distinguish HS. The color code of the dot filling corresponds to the normalized RT and therefore comprises the full range for each HS from dark to white for short to long RT

FindSeries revealed the occurrence of 329 HS based on CF_2_ repeating units (see ESM Table S1). This still includes HS with only two compounds. Reducing this list to HS with at least three individual compounds condensed this dataset to 133 HS (see ESM Table S2). Visualization in a CF_2_-based Kendrick mass defect plot (Fig. 1) easily allows a first overview on the data. Compounds belonging to the same class are aligned horizontally. As a second criterion, increasing retention time with increasing mass is used to identify members of a true HS. This is visualized by color code of the data points in Fig. 1 from dark to white for increasing retention times.

Finally, 70 HS fulfilled the criteria of HS detection with at least 3 homologues and systematic RT shifts. They are marked in green in ESM Table S2.

**Fig. 2:**
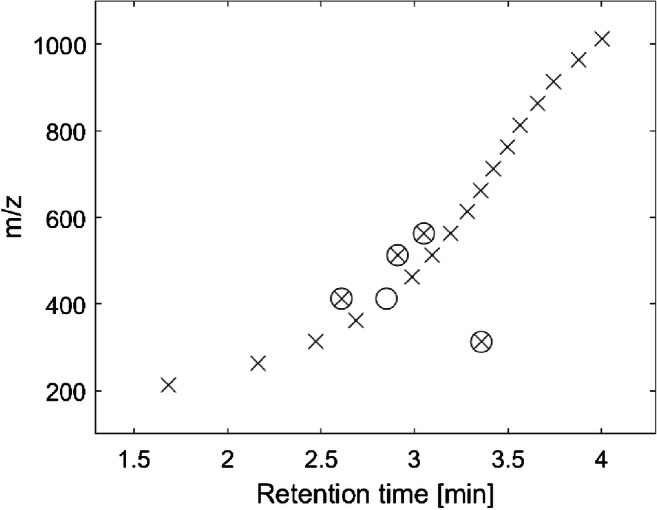
Systematic retention time shift for HS 82 (PFCAs). Legend: multiplication sign, feature from FindSeries following the RT shift trend; encircled multiplication sign, feature from FindSeries not following the RT shift trend; empty circle, missing feature from FindSeries (possible miss from the MFE)

This reduced list of the accurate masses is matched with the OECD database (available in ESM Table S3) for a first tentative identification. For example, HS 82 consists of 20 single features (see ESM Table S1). Plotting these features in a m/z vs. RT diagram (see Fig. 2) shows a clear RT shift for most of the features. Four features obviously differ from the clear trend (marked with encircled multiplication sign), and another feature with m/z 413 at the correct RT is missing (marked with empty circle). The absence of a signal may be due to missing of the compound, low signal intensity, or mismatch in the feature-finding algorithm MFE. All features of this HS could be matched with at least one compound of the OECD PFAS database. The number of matches is indicated in the “Presence” row. The lowest m/z in this HS, m/z 212.9793, was matched with the two isomeric PFCAs heptafluorobutyric acid (CAS number 375-22-4) and heptafluoroisobutyric acid (CAS number 335-10-4). Also, all other homologues of HS 82 could be assigned to PFCAs.

Next, the extracted ion chromatograms (EICs) of the features were checked manually at a mass accuracy of 10 ppm (ESM Fig. S1). Since the MFE algorithm could always miss single features, also missing members of the HS were included and confirmed, for example the presence of the previously discussed missing feature at m/z 413. Since some peaks exhibit shoulders or not well separated peaks in front (m/z 412.9664), the MFE algorithm registered two peaks which were marked with encircled multiplication sign in Fig. 2. m/z 412.9664 (also a fronting peak) was integrated once with a rather short RT and therefore explains the missing feature in Fig. 2. Identification of m/z 412.9664 as PFOA could be confirmed by an authentic standard (ESM Fig. S1.1). The other PFCA homologues of HS 82 could be identified with confidence level 2a. This procedure was repeated for each of the 70 HS which are marked in green in ESM Table S2. This resulted in the identification of the following substance classes: PFSAs, diSAmPAPs, FTUCAs, diPAPs, and *n*:3 PFCAs. Identification based on standards for these evaluated substance classes is shown in Figs. S1.2 to S1.8 (see ESM).

Further identifications based on matches with the OECD PFAS database with only one homologue are shown in ESM Table S3. For example, m/z 497.9457 (line 153 in ESM Table S3) was assigned to FOSA and finally confirmed by an authentic standard (ESM Fig. S1.3). Chromatograms were checked manually for further perfluoroalkanesulfonamide (FASA) homologues which were not detected in this case. This procedure was repeated for most compounds in ESM Table S3. Compounds are discarded which are likely to be false positives, for example if no ionization in ESI negative is expected. This procedure further identified the following substance classes: FASA, *N*-methyl perfluoroalkanesulfonamide (MeFASA), *N*-ethyl perfluoroalkanesulfonamide (EtFASA), and *N*-ethylperfluoro-1-alkanesulfonamidoacetic acid (EtFASAA) (one compound each). A complete list of all findings, including the identification level according to Schymanski et al. [39], is presented in Table 1, and the identified substance classes are discussed subsequently.

**Table 1:** Identified PFAS classes and their number of homologues in sample 1

HS no.	Identified as	Number of homologues	Mass range	Identification level
252	diPAPs	8	689–1389	Level 2a (1× level 1*)
M1	triPAPs	> 4	N/A	Level 3 (1× level 1*)
40	FTUCAs	5	557–957	Level 2b
265	*n*:3 PFCAs	5	441–841	Level 2a (1× level 1*)
82	PFCAs	16	263–1013	Level 2a (1× level 1*)
17	diSAmPAPs	3	1103–1263	Level 2a (1× level 1*)
M2	EtFASAAs	1	584	Level 1
M3	MeFASAs	1	512	Level 3
M4	EtFASAs	1	526	Level 3
M5	FASAs	1	498	Level 1
321	PFSAs	5	399–599	Level 2a (1× level 1*)

Details can be found in ESM Table S1 under the corresponding HS no. Manually detected compound classes are marked with an M. For accurate masses of all homologues, see ESM Table S4

*For reference standards, see ESM Table S4

### diPAPs (HS 252) and triPAPs (HS M1)

A total of 8 diPAP homologues were detected (corresponding to 4:2/6:2, 6:2/6:2, 6:2/8:2, 8:2/8:2, 8:2/10:2, 10:2/10:2, 10:2/12:2, 12:2/12:2, and 12:2/14:2) and confirmed by an authentic standard of 6:2/8:2 diPAP. diPAPs are known to be used in food contact paper to act as grease repellant [6], making their occurrence in the paper sludge that was applied on the arable land plausible. Manual inspection of the diPAP chromatograms revealed further peaks with longer retention times. However, these peaks were suspected to originate from triPAPs which are impurities in diPAP products [6]. During ESI, triPAPs are fragmented in-source by the loss of a fluorotelomer alcohol (FTOH) group and therefore appear as diPAPs in LC-MS chromatograms [6, 40, 41]. However, from one triPAP, multiple peaks can result from in-source fragmentation (e.g., 6:2/8:2/10:2 triPAP) depending on which FTOH chain is lost. As a result, an unequivocal identification of triPAPs is challenging, also because authentic standards are not available for all homologues. Therefore, only 6:2/6:2/6:2 triPAP could be identified with an authentic standard, and the other homologues could be tentatively identified and were assigned to confidence level 3.

### diPAP TPs: FTUCAs (HS 40), *n*:3 PFCAs (HS 265), and PFCAs (HS 82)

DiPAPs and triPAPs can be transformed into FTOHs by cleavage of the phosphate ester bonds [42], which can be further degraded to several compound classes like FTUCAs, PFCAs, or *n*:3 PFCAs [43].

Five homologues of FTUCAs have been detected in sample 1 (10:2, 12:2, 14:2, 16:2, and 18:2). Based on a study by Liu and Avendano [44], 8:2 FTUCA is a precursor of several PFCAs (perfluorohexanoic, perfluoroheptanoic, and perfluorooctanoic acids). Longer FTUCA homologues are hence expected to transform into longer-chain PFCAs. An authentic standard for 8:2 FTUCA was used to evaluate the mass fragmentation, which is characterized by the loss of HFCO_2_ at a collision energy of 20 eV. The same fragments were observed for the FTUCA congeners that are found in the soil sample (data available in ESM Figs. S1.11–S1.16), and confidence level 2b was hence assigned to this HS.

Further degradation of FTUCAs can produce *n*:3 PFCAs of which five were detected (7:3, 9:3, 11:3, 13:3, and 15:3) and confirmed by a reference standard of 7:3 PFCA. These *n*:3 PFCAs are suggested to further break down into PFCAs [44]. In total, 16 different PFCA homologues were found, ranging from carbon chain lengths C_5_ up to C_20_. PFOA was used as reference standard. PFCAs are rather persistent compounds which will hardly be further degraded in the environment [43].

### diSAmPAPs (HS 17)

Three homologues of the diSAmPAP class (C_7_/C_8_, C_8_/C_8_, and C_8_/C_9_) were detected in sample 1. C_8_/C_8_ was confirmed by a standard and is with 98% of the intensity of all diSAmPAPs by far the most abundant homologue in sample 1. This points to the use of PFAS products on a C8-based chemistry. DiSAmPAP is a chemical that was used directly in food contact papers and had a high production volume until 2002 [31].

### diSAmPAP TPs: EtFASAAs (HS M2), MeFASAs (HS M3), EtFASAs (HS M4), FASAs (HS M5), and PFSAs (HS 321)

The degradation of diSAmPAPs in marine sediments showed several TPs like EtFASAAs, EtFASAs, FASAs, and PFSAs [22], which could be confirmed in our work. MeFASA most likely is an impurity in diSAmPAP products. Identification of diSAmPAPs, EtFASAAs, FASAs, and PFSAs was confirmed by a C_8_-based reference standard of each compound class.

As already expected from the dominant occurrence of C_8_/C_8_ diSAmPAP, no other homologues than C_8_ could be detected for EtFASAA, MeFASA, EtFASA, and FASA. The intensity of PFOS was higher than 99.5% compared to the sum of the intensities from all measured PFSAs. This finding suggests PFOS as the degradation product of C_8_/C_8_ diSAmPAPs.

### False positive assignments

PFPIAs were first suggested to occur in the contaminated soil sample (see ESM Table S1, HS 4). Even though only higher homologues than the available reference standard of the C_6_/C_6_-PFPIA (m/z 700.9221) were detected in the soil, the standard was used to record an MS/MS spectrum at 40 eV (ESM Fig. S1.9). Two fragments at m/z 62.9638 (PO_2_) and m/z 400.9415 (C_6_F_14_O_2_P) have been detected. While the fragment m/z 400.9415 is dependent on the PFPIA chain length, the PO_2_ fragment should occur for any PFPIA homologue but did not. Therefore, PFPIA homologues could not be identified (ESM Fig. S1.10).

### Further unknown PFASs

In addition to the identified PFASs, the data still contain many unidentified PFASs occurring in HS with CF_2_, CF_2_O, and C_2_F_4_O repeating units. FindSeries suggested 50 HS based on CF_2_ repeating units, 23 HS based on CF_2_O, and 13 HS based on C_2_F_4_O, each with a minimum number of 5 individual compounds (data available in ESM Tables S5 and S6). While some of them may be false positives for example due to in-source fragmentation or adduct formation, we expect also the presence of true positives which are absent in the OECD PFAS database and may be degradation products of the original PFAS products.

#### Results for samples 2, 3, and 4 and comparison of all four samples

Analogous to sample 1, samples 2, 3, and 4 were evaluated and the results are summarized in Tables 2, 3, and 4. Detailed information and accurate masses are presented in ESM Table S4.

**Table 2:** Identified PFAS classes and their number of homologues in sample 2

HS no.	Identified as	Number of homologues	Mass range	Identification level
248	diPAPs	7	789–1389	Level 2a (1× level 1)
M1	triPAPs	> 4	N/A	Level 3 (1× level 1)
86	PFCAs	17	213–1013	Level 2a (1× level 1)
23	diSAmPAPs	1	1203	Level 1
M2	EtFASAAs	1	584	Level 1
M3	FASAs	1	498	Level 1
312	PFSAs	7	299–599	Level 2a (1× level 1)
166	FTSAs	6	427–927	Level 2a (1× level 1)

Manually detected compound classes are marked with an M. For accurate masses of all homologues, see ESM Table S4

**Table 3:** Identified PFAS classes and their number of homologues in sample 3

HS no.	Identified as	Number of homologues	Mass range	Identification level
250	diPAPs	8	689–1389	Level 2a (1× level 1)
M1	triPAPs	> 4	N/A	Level 3 (1× level 1)
265	*n*:3 PFCAs	5	441–841	Level 2a (1× level 1)
M2	PFCAs	15	213–963	Level 2a (1× level 1)
16	diSAmPAPs	1	1203	Level 1
M3	EtFASAAs	1	584	Level 1
M4	MeFASA	1	512	Level 3
M5	FASAs	1	498	Level 1
M6	PFSAs	4	399–549	Level 2a (1× level 1)

Manually detected compound classes are marked with an M. For accurate masses of all homologues, see ESM Table S4

**Table 4:** Identified PFAS classes and their number of homologues in sample 4

HS no.	Identified as	Number of homologues	Mass range	Identification level
290	diPAPs	7	789–1389	Level 2a (1× level 1)
M1	triPAPs	> 4	N/A	Level 3 (1× level 1)
M2	PFCAs	14	213–913	Level 2a (1× level 1)
M3	diSAmPAPs	1	1203	Level 1
M4	EtFASAAs	1	584	Level 1
M5	FASAs	1	498	Level 1
M6	PFSAs	4	399–549	Level 2a (1× level 1)
192	FTSAs	4	627–927	2a

Manually detected compound classes are marked with an M. For accurate masses of the homologues, see ESM Table S4

Also samples 2, 3, and 4 are characterized by the presence of diPAPs (7 to 8 homologues: 4:2/6:2, 6:2/6:2, 6:2/8:2, 8:2/8:2, 8:2/10:2, 10:2/10:2, 10:2/12:2, 12:2/12:2, and 12:2/14:2), C_8_/C_8_ diSAmPAP (only 1 dominating homologue), and their TPs. In all four samples, PFCAs reveal a large number of homologues between 14 and 17. *n*:3 PFCAs were detected only in samples 1 and 3 and FTSAs only in samples 2 and 4. FTSAs are not expected to be TPs of either diPAPs or diSAmPAPs. Hence, they could either occur from the degradation of another, so-far unidentified or already fully degraded, precursor.

### Estimated concentration levels

Based on the response factors of the authentic standards for single PFASs at one concentration level (each at 5 μg/L corresponding to 10 μg/kg soil), concentration levels of these PFASs have been estimated for the soil samples. In soil sample 1, PFOA occurred at about 60 μg/kg (ESM Fig. S1.1), PFOS at 100 μg/kg (ESM Fig. S1.4), EtFOSAA at 100 μg/kg (ESM Fig. S1.5), 6:2/8:2 diPAP at 210 μg/kg (ESM Fig. S1.7), and diSAmPAP at 630 μg/kg (ESM Fig. S1.8). In sample 2, PFOA was found at 250 μg/kg (ESM Fig. S1.46) and 6:2/8:2 diPAP at 20 μg/kg (ESM Fig. S1.47); in sample 3, PFOA was 240 μg/kg (ESM Fig. S1.48) and 6:2/8:2 diPAP 90 μg/kg (ESM Fig. S1.49); in sample 4, PFOA had 90 μg/kg (ESM Fig. S1.50) and 6:2/8:2 diPAP 70 μg/kg (ESM Fig. S1.51).

Based on these results, diPAPs and PFCAs are suggested to be the major contaminants in all four samples. In sample 1, diSAmPAP and its TPs (PFOS and EtFOSAA) are the dominating contaminants followed by diPAPs and its TPs.

## Conclusions

Paper sludge contaminated with PFAS products caused a complex contamination on agricultural soils, since different not further characterized PFAS products and a considerable number of different TPs contribute to the overall contamination. LC-high-resolution mass spectrometry (LC-HRMS) data revealed the complex contamination. The identification of homologous series (HS) of original compounds and transformation products (TPs) by Kendrick mass analysis and systematic retention time shifts was a successful strategy to confidently identify PFASs also with a very limited number of authentic standards. DiPAPs (7–8 homologues, 4:2/6:2 to 12:2/14:2) and diSAmPAP (C_8_/C_8_) have been identified as the major contaminants of the soils. These PFASs were also used in products for paper impregnation. All other major compound classes could be linked to known TPs of these products with the only exception of FTSAs. The low availability of authentic standards and suitable database entries especially for TPs mainly limit the identification of numerous unknowns which are still in the HRMS data. The application of the proposed workflow on technical products in original form and after degradation tests is a further promising approach to increase the fraction of identified PFAS and their TPs in environmental samples.

## Electronic supplementary material

ESM 1 (PDF 1766 kb)

ESM 2 (XLSX 156 kb)

ESM 3 (XLSX 22 kb)

## Abbreviations

AIF: All-ion fragmentation
Da: Dalton
diPAP: Polyfluorinated dialkylated phosphate ester
diSAmPAP: *N*-Ethyl perfluorooctane sulfonamide ethanol–based phosphate diester
EIC: Extracted ion chromatogram
ESI: Electrospray ionization
EtFASA: *N*-Ethyl perfluoroalkanesulfonamide
EtFASAA: *N*-Ethylperfluoro-1-alkanesulfonamidoacetic acid
EtFOSAA: *N*-Ethylperfluoro-1-octanesulfonamidoacetic acid
FASA: Perfluoroalkanesulfonamide
FOSA: Perfluorooctanesulfonamide
FTOH: Fluorotelomer alcohol
FTSA: Fluorotelomer sulfonic acid
FTUCA: Fluorotelomer unsaturated carboxylic acid
GenX: Ammonium 2,3,3,3-tetrafluoro-2-(heptafluoropropoxy) propanoate
HRMS: High-resolution mass spectrometry
HS: Homologous series
KM: Kendrick mass
KMD: Kendrick mass defect
LC: Liquid chromatography
LTZ: Landwirtschaftliches Technologiezentrum Augustenberg
MeFASA: *N*-Methyl perfluoroalkanesulfonamide
MeOH: Methanol
MFE: Molecular feature extraction
NH_4_Ac: Ammonium acetate
OM: Observed mass
PFAAs: Perfluoroalkyl acids
PFASs: Poly- and perfluoroalkyl substances
PFCAs: Perfluorocarboxylic acids
PFHpA: Perfluoroheptanoic acid
PFNA: Perfluorononanoic acid
PFOA: Perfluorooctanoic acid
PFOS: Perfluorooctanesulfonic acid
PFPIA: Perfluorinated phosphonic acid
PFSAs: Perfluorosulfonic acids
PP: Polypropylene
rcf: Relative centrifugal force
RT: Retention time
TP: Transformation product
triPAP: Polyfluorinated trialkylated phosphate ester
