# Supplementary material for: LC-MS screening of poly- and perfluoroalkyl substances in contaminated soil by Kendrick mass analysis
Source: Anal Bioanal Chem. 2020 Jan 9;412(20):4797–805. doi: 10.1007/s00216-019-02358-0 (PMC7334281; doi:10.1007/s00216-019-02358-0)
Supplement: Supplementary file 1 — (PDF 1766 kb) [file 216_2019_2358_MOESM1_ESM.pdf]

Analytical and Bioanalytical Chemistry

Electronic Supplementary Material

LC-MS screening of poly- and perfluoroalkyl substances in contaminated soil by Kendrick mass analysis

Boris Bugsel, Christian Zwiener

Additional files available under 10.1007/s00216-019-02358-0

Contents

Abbreviations and Acronyms 3
Examples for structures of compounds from different PFAS classes 4
Example for manual verification of homologues 6
Sample 1: Confirmation of the identification with an authentic standard 7
Sample 2: Confirmation of the identification with an authentic standard 22
Sample 3: Confirmation of the identification with an authentic standard 29
Sample 4 Confirmation of the identification with an authentic standard 37
Targeted MS/MS measurements 48
Confirmation of the identification with an authentic standard for samples 2, 3, and 4 52

Abbreviations and Acronyms

AIF	All-ion fragmentation
Da	Dalton
diPAP	Polyfluorinated dialkylated phosphate ester
diSAmPAP	N-ethyl perfluorooctane sulfonamide ethanol-based phosphate diester
EIC	Extracted Ion Chromatogram
ESI	Electrospray Ionization
EtFASA	N-ethyl perfluoroalkanesulfonamide
EtFASAA	N-ethylperfluoro-1-alkanesulfonamidoacetic acid
EtFOSAA	N-ethylperfluoro-1-octanesulfonamidoacetic acid
FASA	Perfluoroalkanesulfonamide
FOSA	Perfluorooctanesulfonamide
FTOH	Fluorotelomer alcohol
FTSA	Fluorotelomer sulfonic acid
FTUCA	Fluorotelomer unsaturated carboxylic acid
GenX	Ammonium 2,3,3,3-tetrafluoro-2-(heptafluoropropoxy)-propanoate
HRMS	High resolution mass spectrometry
HS	Homologous series
KM	Kendrick mass
KMD	Kendrick mass defect
LC	Liquid Chromatography
LTZ	Landwirtschaftliches Technologiezentrum Augustenberg
MeFASA	N-methyl perfluoroalkanesulfonamide
MeOH	Methanol
MFE	Molecular feature extraction
NH4Ac	Ammonium acetate
OM	Observed Mass
PFAAs	Perfluoroalkyl acids
PFASs	Poly- and perfluorinated alkylated substances
PFCAs	Perfluorocarboxylic acids
PFHpA	Perfluoroheptanoic acid
PFNA	Perfluorononanoic acid
PFOA	Perfluorooctanoic acid
PFOS	Perfluorooctanesulfonic acid
PFPIA	Perfluorinated phosphinic acid
PFSAs	Perfluorosulfonic acids
PP	Polypropylene
rcf	Relative centrifugal force
RT	Retention time
TP	Transformation product
triPAP	Polyfluorinated trialkylated phosphate ester

Examples for structures of compounds from different PFAS classes

This table contains examples for structures of identified PFAS classes. Further PFAS homologues can be formed by variation of the number of CF2 units.

PFSAs 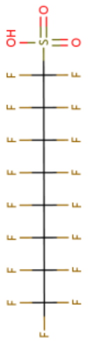	PFPIAs 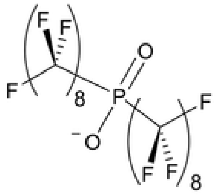	FTUCAs 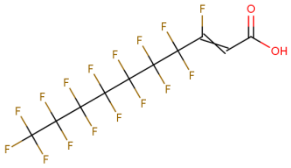	FASAs 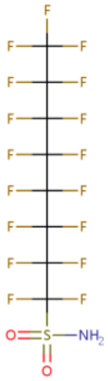
diPAPs 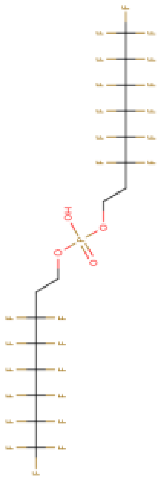	EtFASAAs 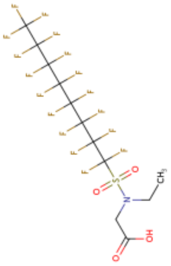	n:3 PFCAs 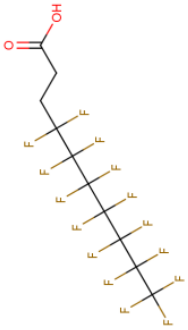	PFCAs 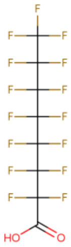
MeFASAs 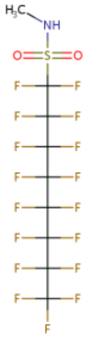	FTSAs 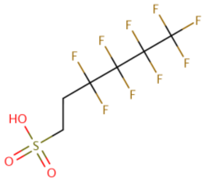	diSAmPAPs 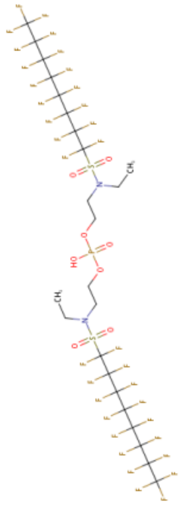	EtFASAs 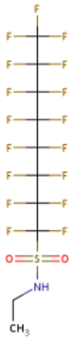

Confirmation of the identification

The instrumental parameters used for acquisition of the chromatograms were generally the same as described in the methods section of the paper. But for soil samples a 1:10 dilution was injected (5 µL) and the LC gradient was slightly changed and started at 25% B with a linear increase to 85% B at 4 min and to 100% B at 5 min, isocratic for another 15 min.

All extracted ion chromatograms in this document were extracted with a 10 ppm mass window with the exception of the targeted MS/MS data. The top chromatogram shows the reference standard (each at 5 µg/L corresponding to 10 µg/kg soil), the bottom chromatogram the soil sample (1:10 dilution) in each case.

Example for manual verification of homologues

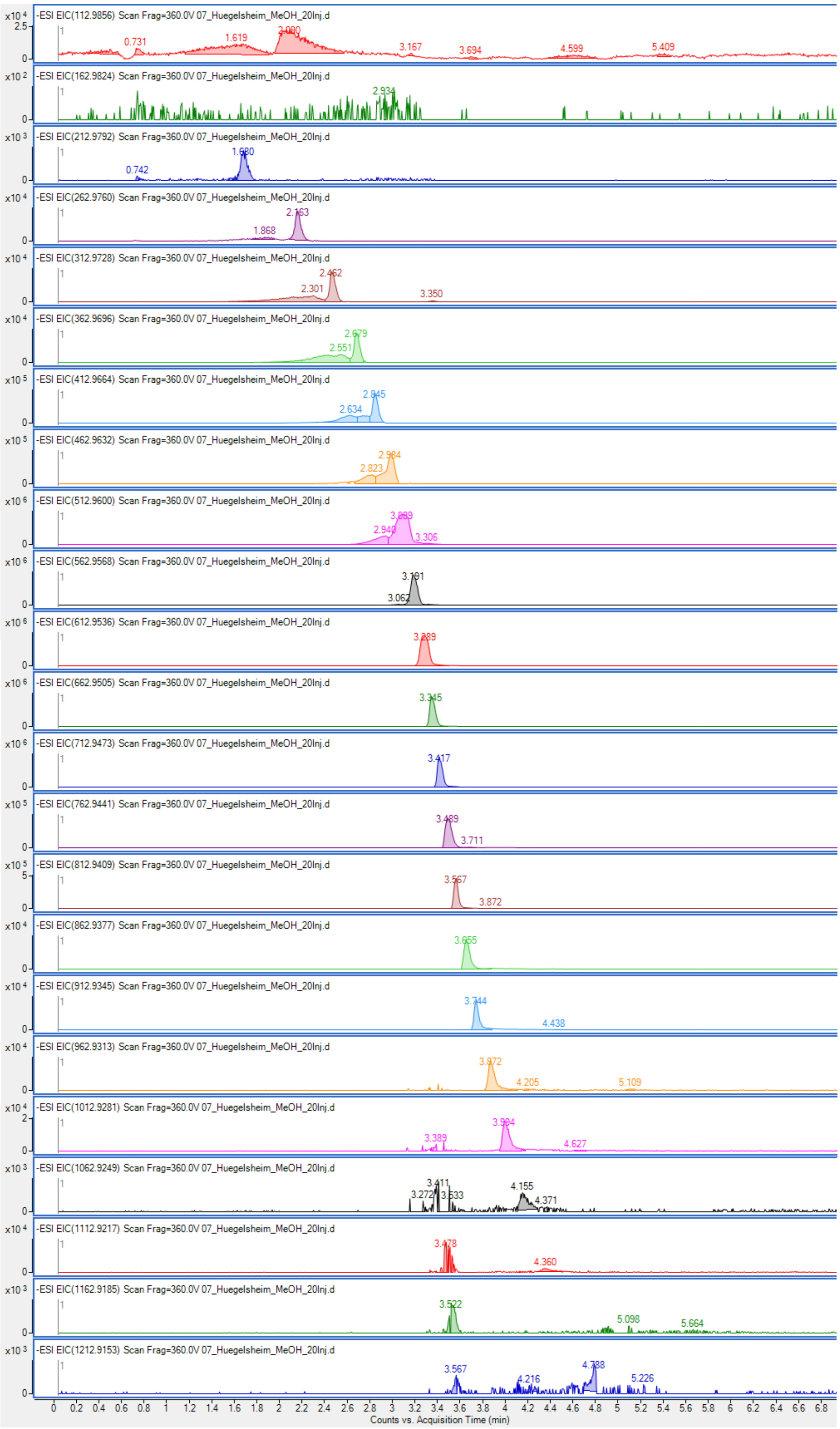

Fig. S1 EICs of all theoretical homologues of the PFCA substance class (m/z 113 - 1213) based on accurate mass and systematic retention time shift

Sample 1: Confirmation of the identification with an authentic standard

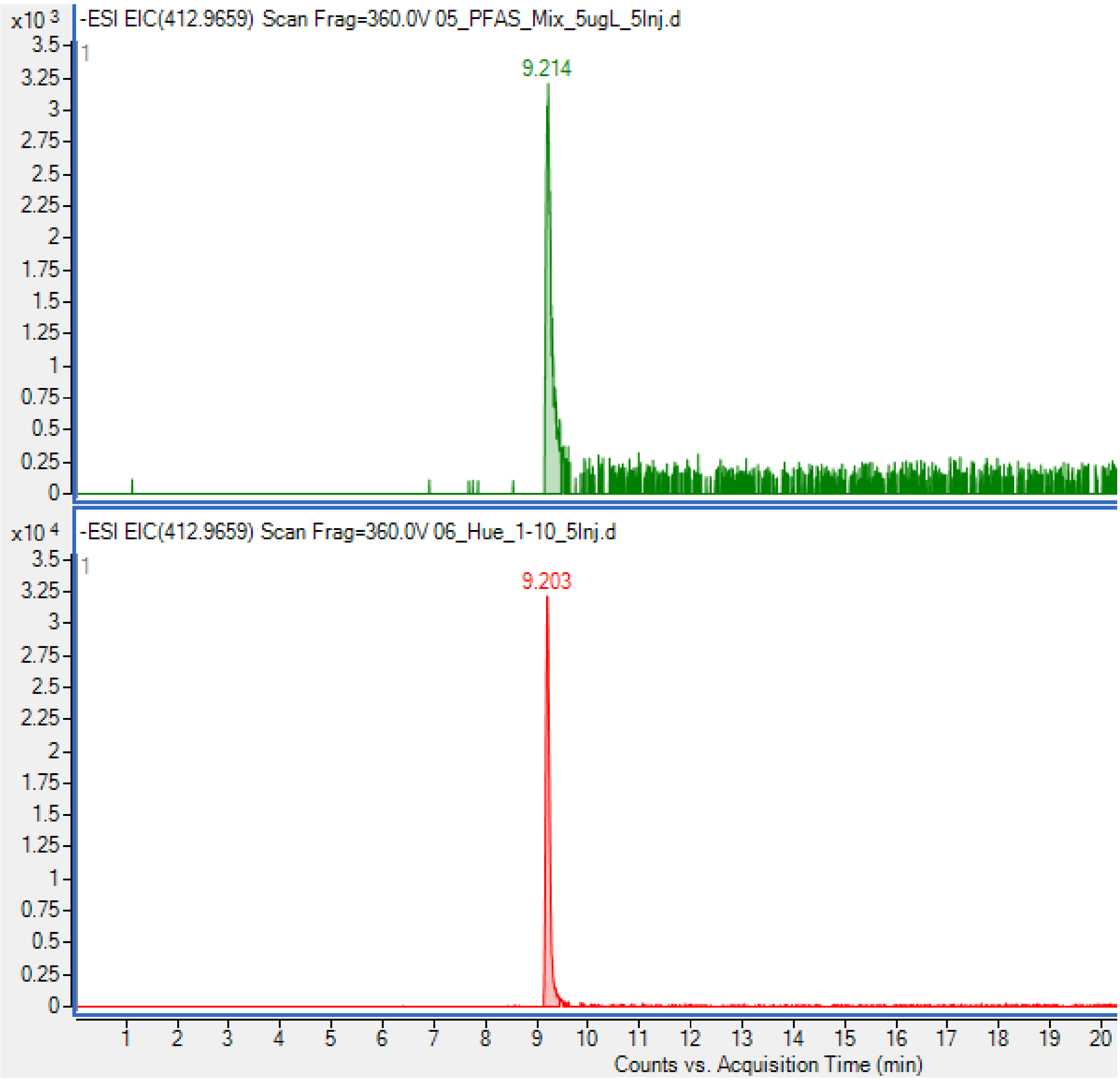

Fig. S1.1 Top: EIC of PFOA standard 5 µg/L, bottom: 1/10 dilution of sample 1

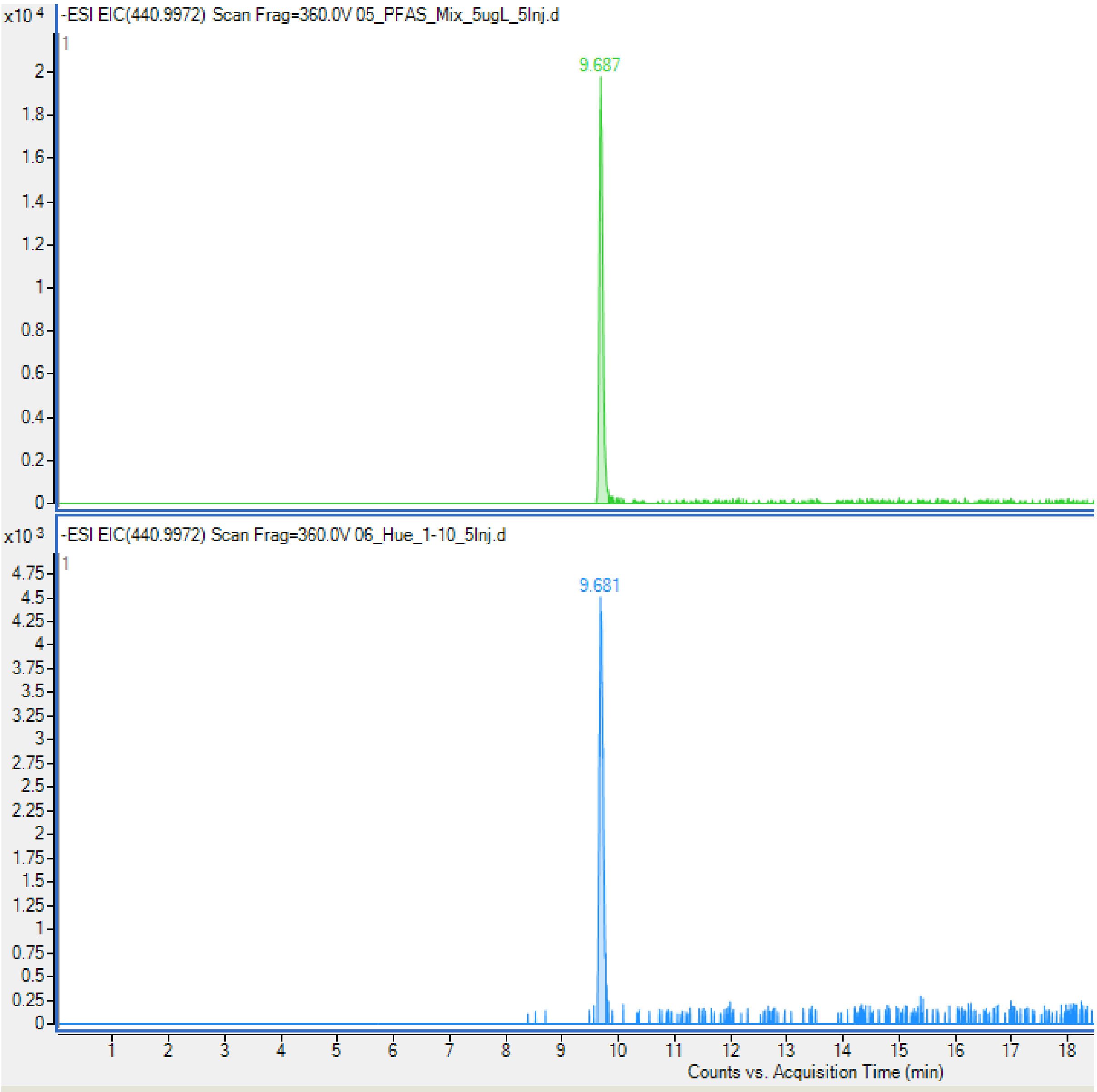

Fig. S1.2 Top: EIC of 7:3 PFCA standard 5 µg/L, bottom: 1/10 dilution of sample 1

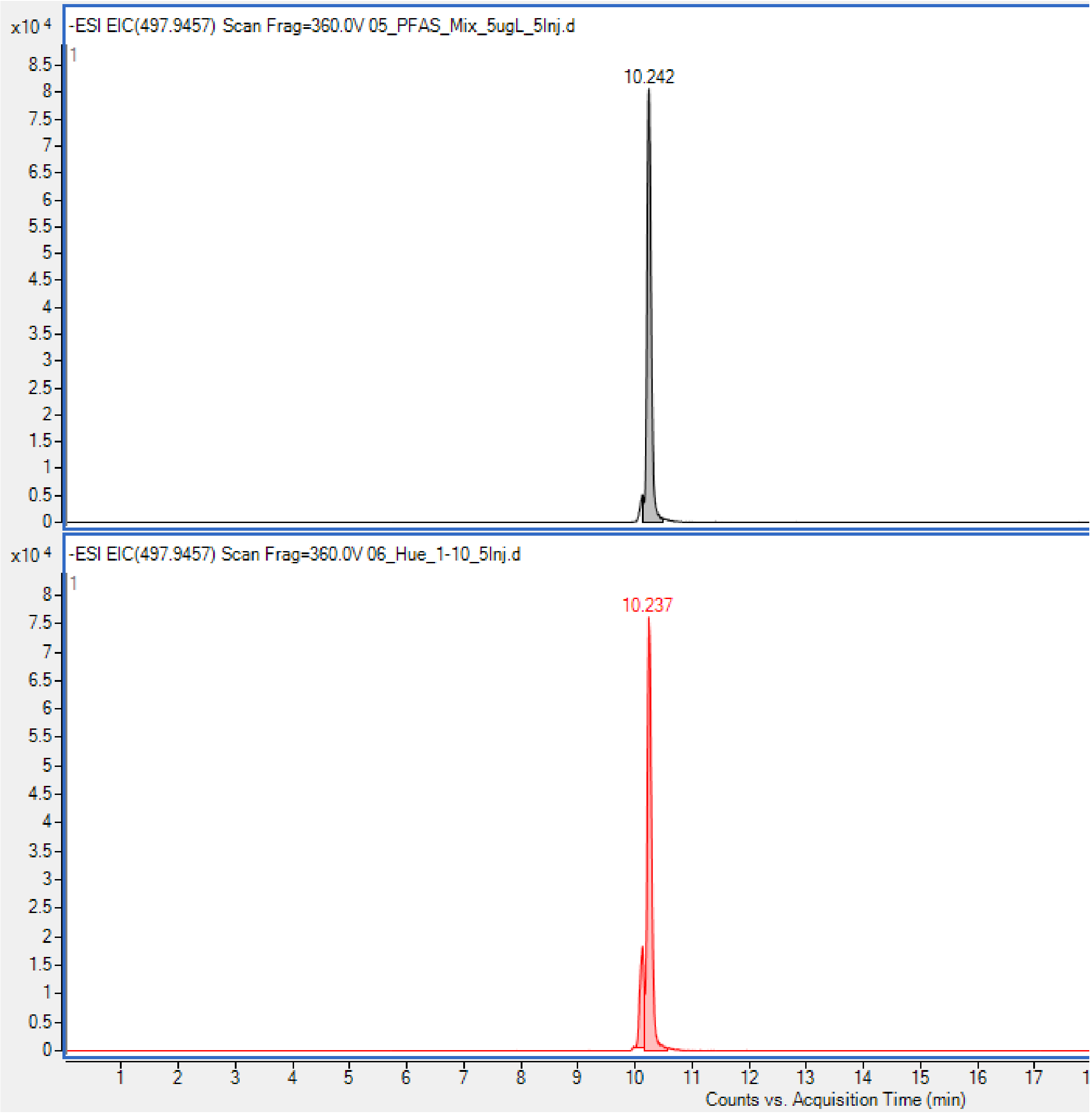

Fig. S1.3 Top: EIC of FOSA standard 5 µg/L, bottom: 1/10 dilution of sample 1

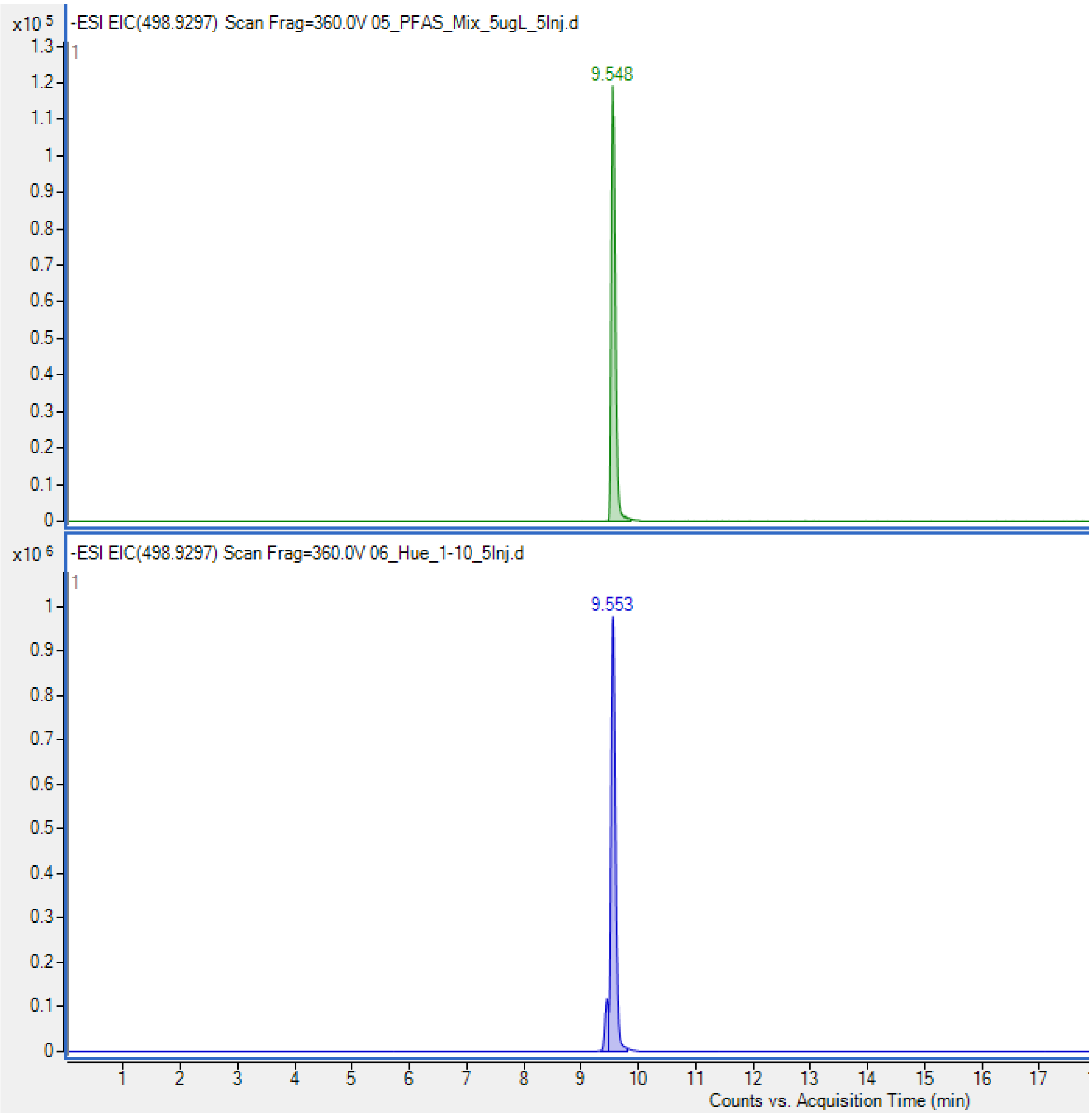

Fig. S1.4 Top: EIC of PFOS standard 5 µg/L, bottom: 1/10 dilution of sample 1

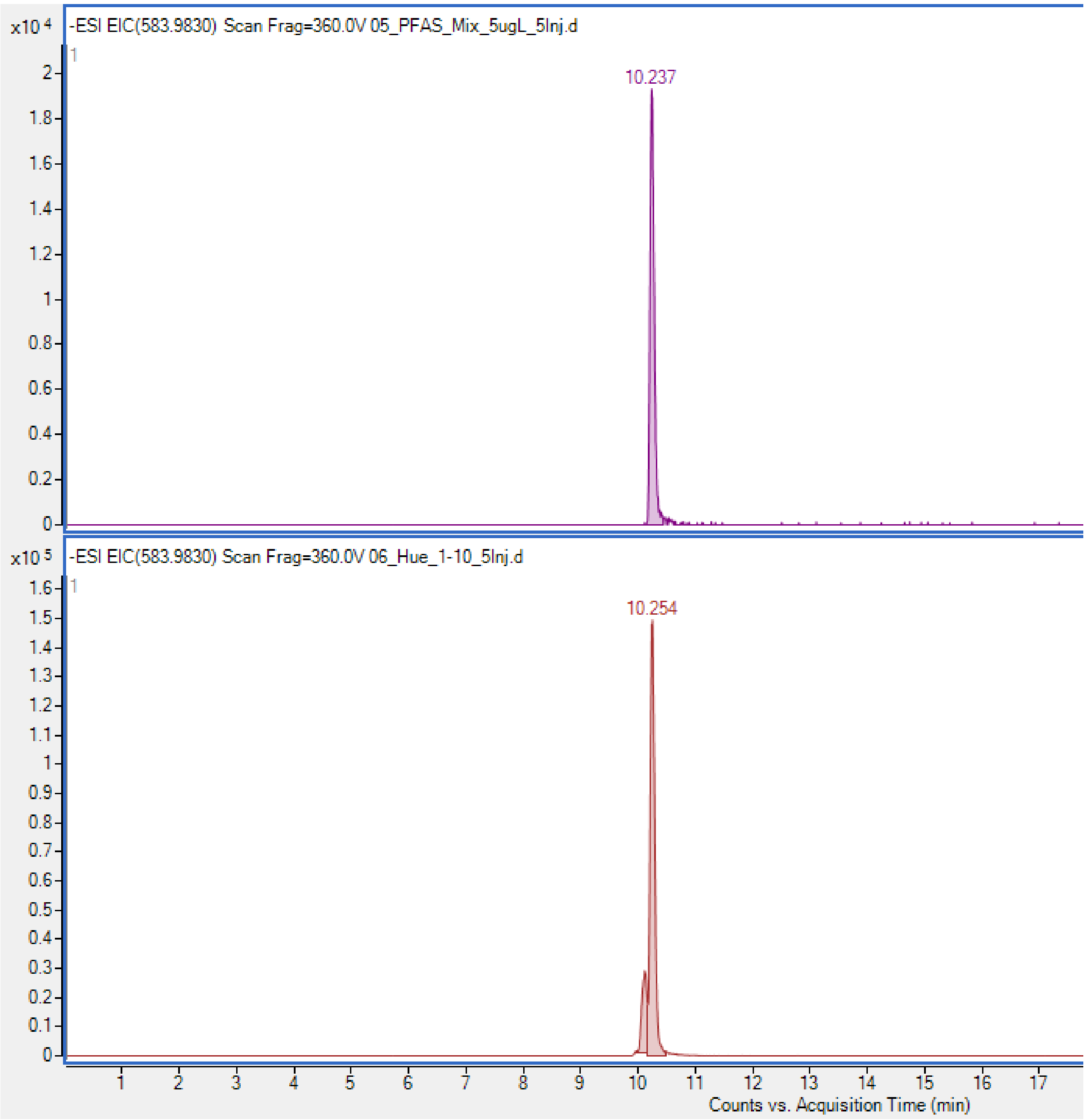

Fig. S1.5 Top: EIC of EtFOSAA standard 5 µg/L, bottom: 1/10 dilution of sample 1

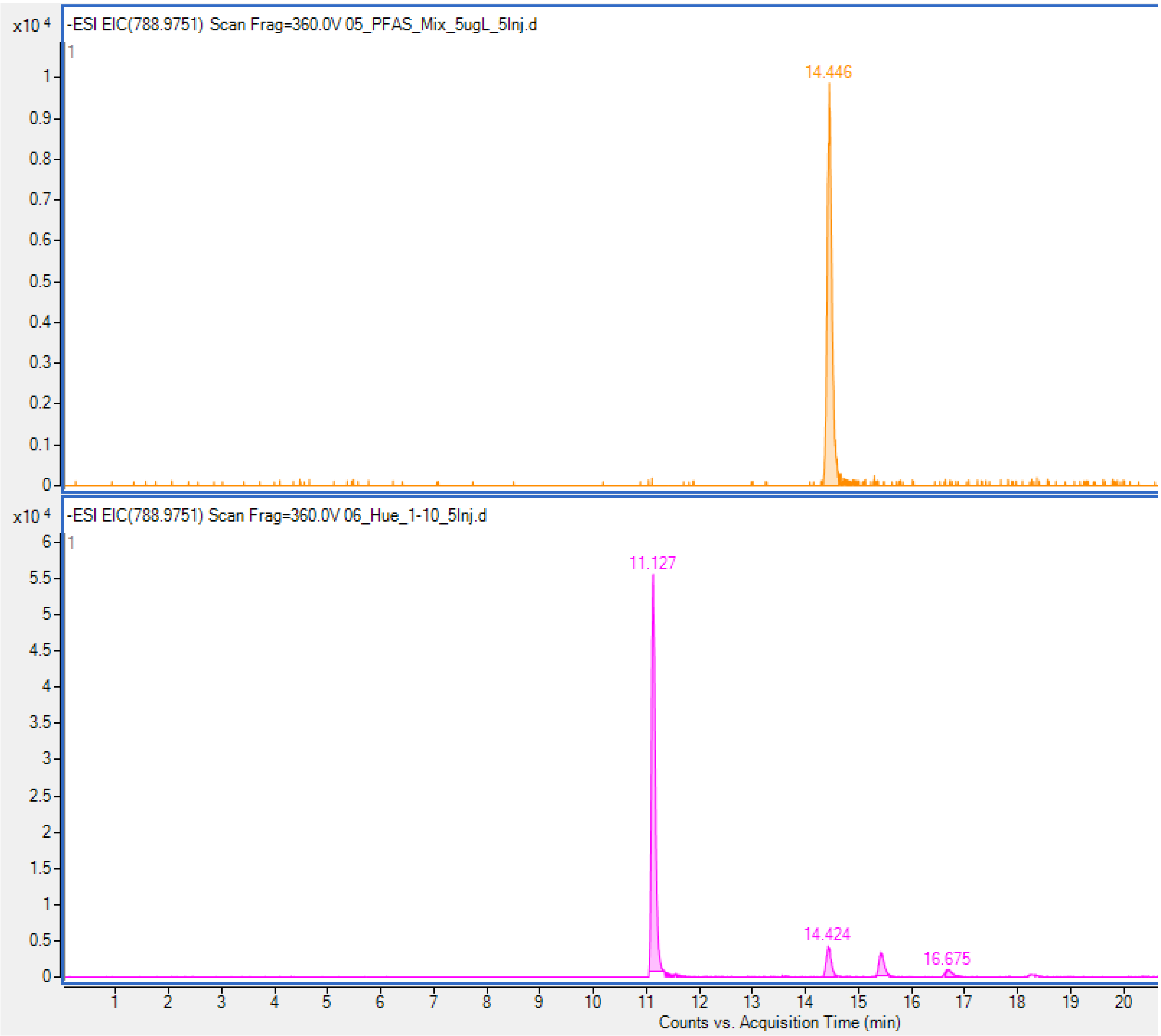

Fig. S1.6 Top: 6:2 / 6:2 / 6:2 triPAP standard 5 µg/L, bottom: 1/10 dilution of sample 1. The diPAP peak and further triPAP peaks in sample 1 are visible

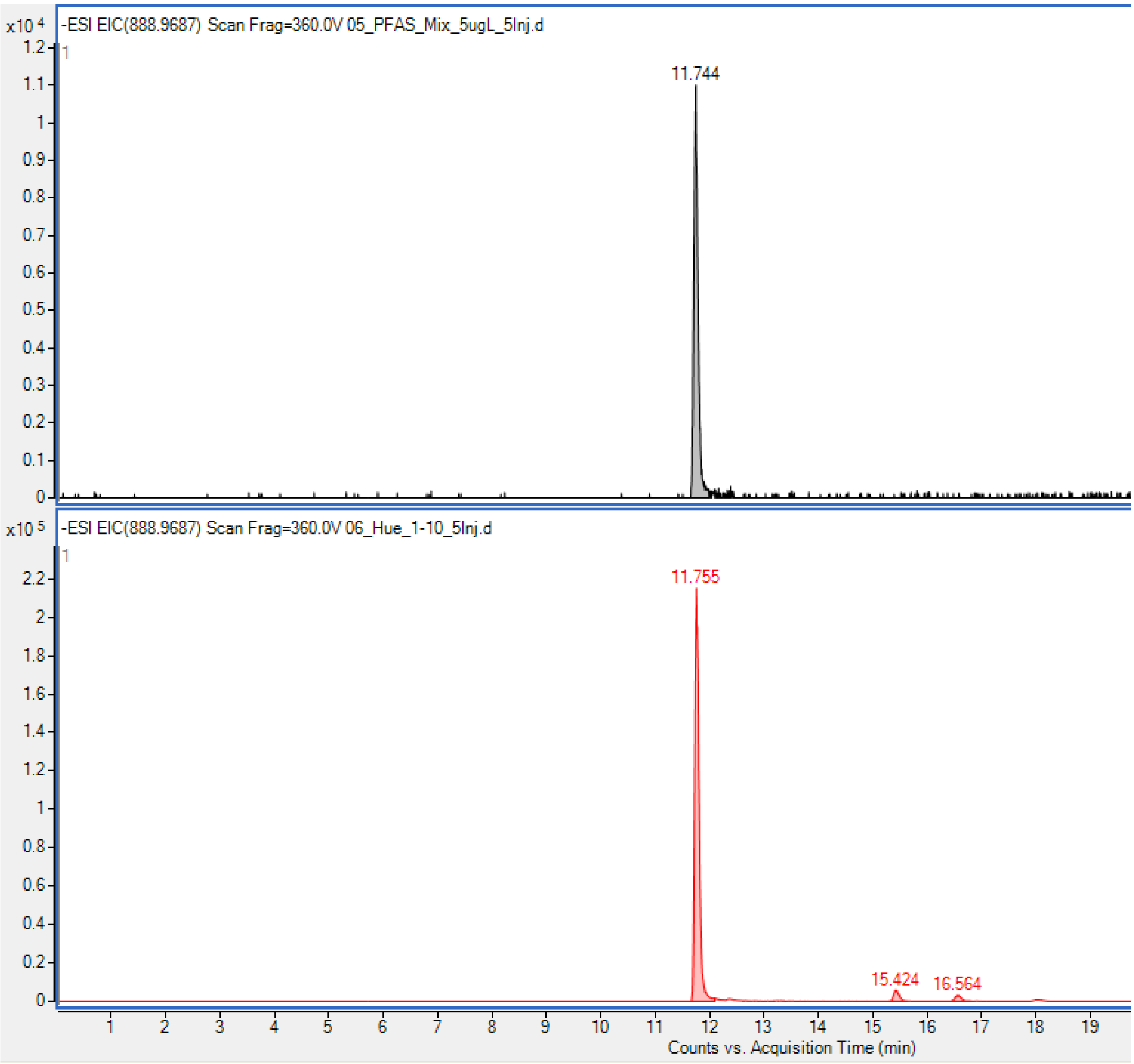

Fig. S1.7 Top: EIC of 6:2 / 8:2 diPAP standard 5 µg/L, bottom: 1/10 dilution of sample 1. TriPAP peaks are visible in sample 1

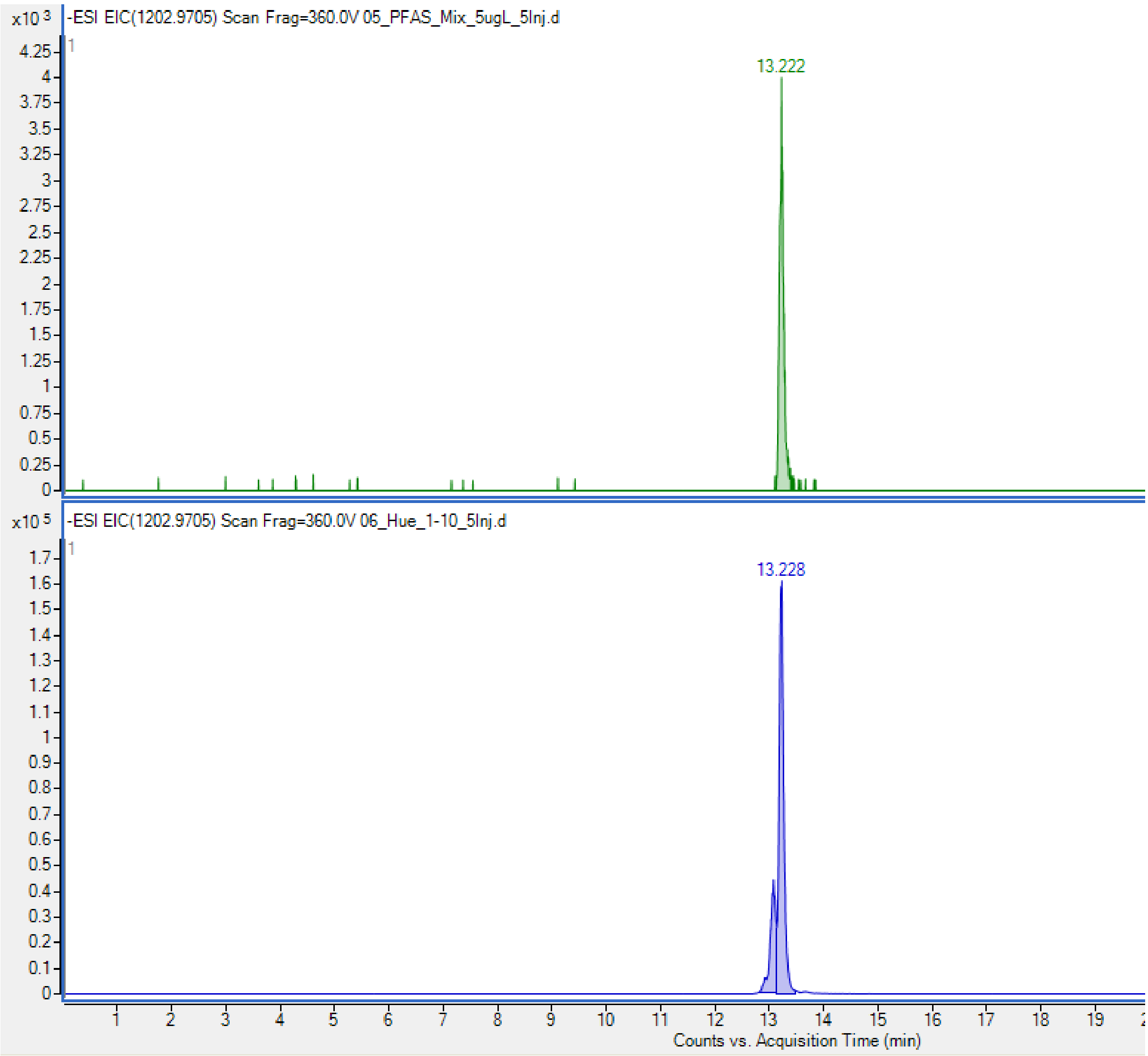

Fig. S1.8 Top: EIC of diSAmPAP standard 5 µg/L, bottom: 1/10 dilution of sample 1

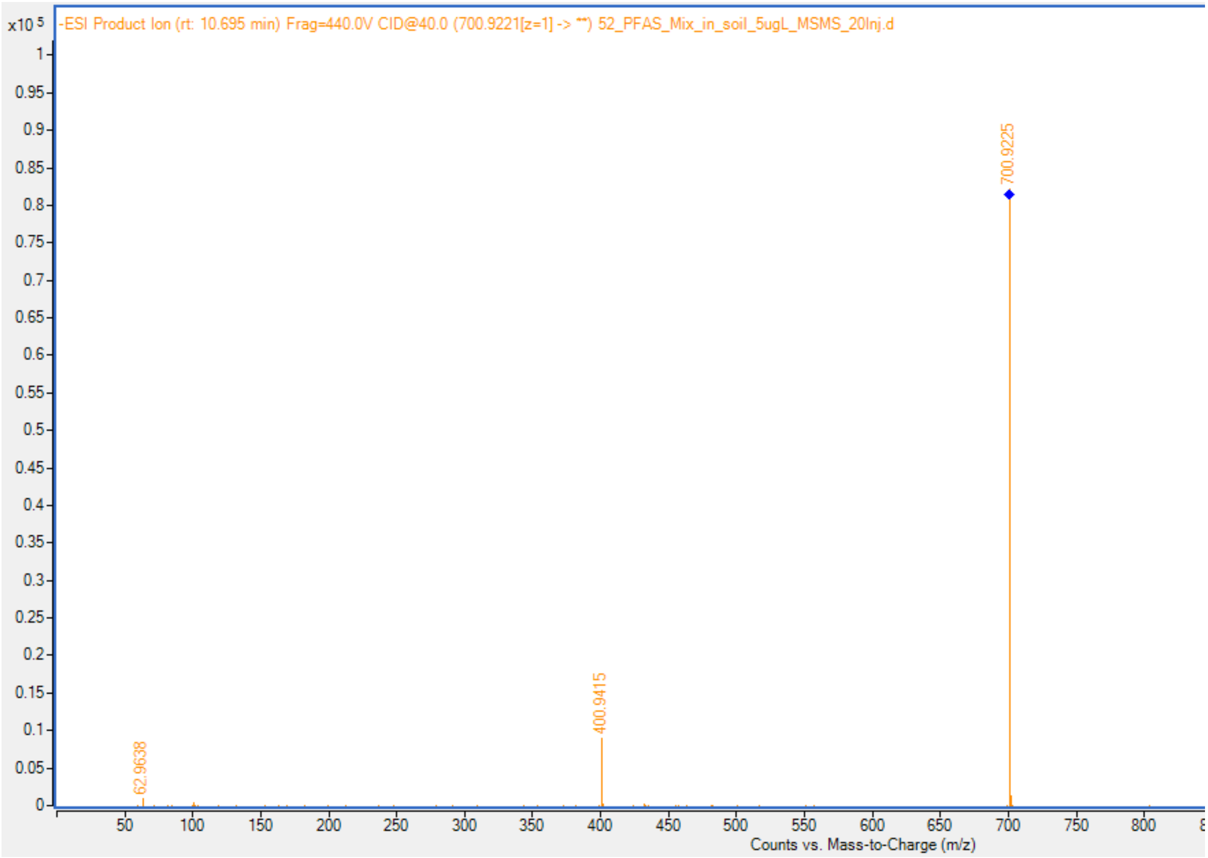

Fig. S1.9 Recorded MS/MS spectrum of C6 PFPIA standard 5 µg/L @ 40eV

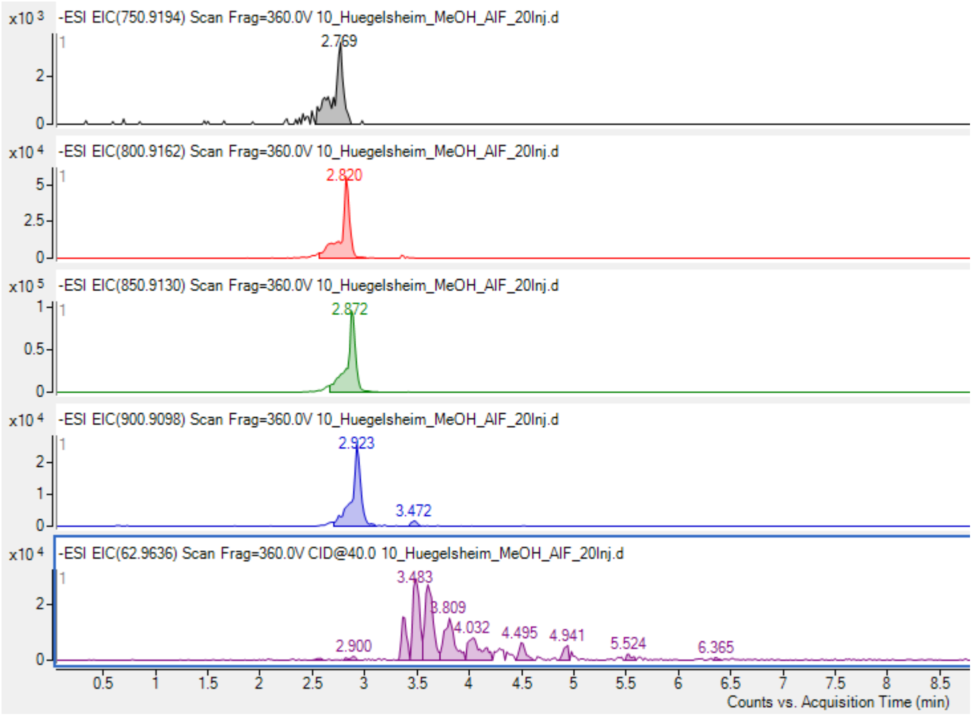

Fig. S1.10 Chromatogram of PFPIA masses and the fragment m/z 62.9638 @40 eV (AIF measurement). Chromatograms of m/z 750.9194 (black), 800.9162 (red), 850.9130 (green), 900.9098 (blue) (these values fit the PFPIA substance class) and the fragment m/z 62.9638 @40 eV (purple). No coelution can be observed

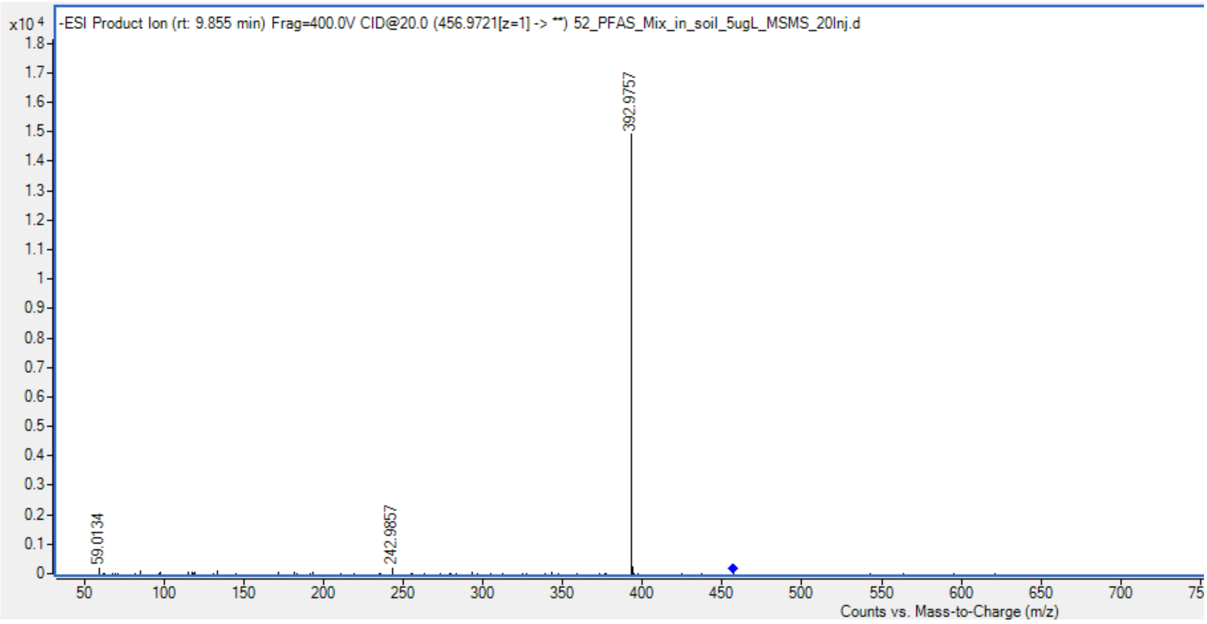

Fig. S1.11 MSMS of 8:2 FTUCA standard 5 µg/L @ 20ev

Dominant peak at 392.9757: Corresponding to loss of CO2HF

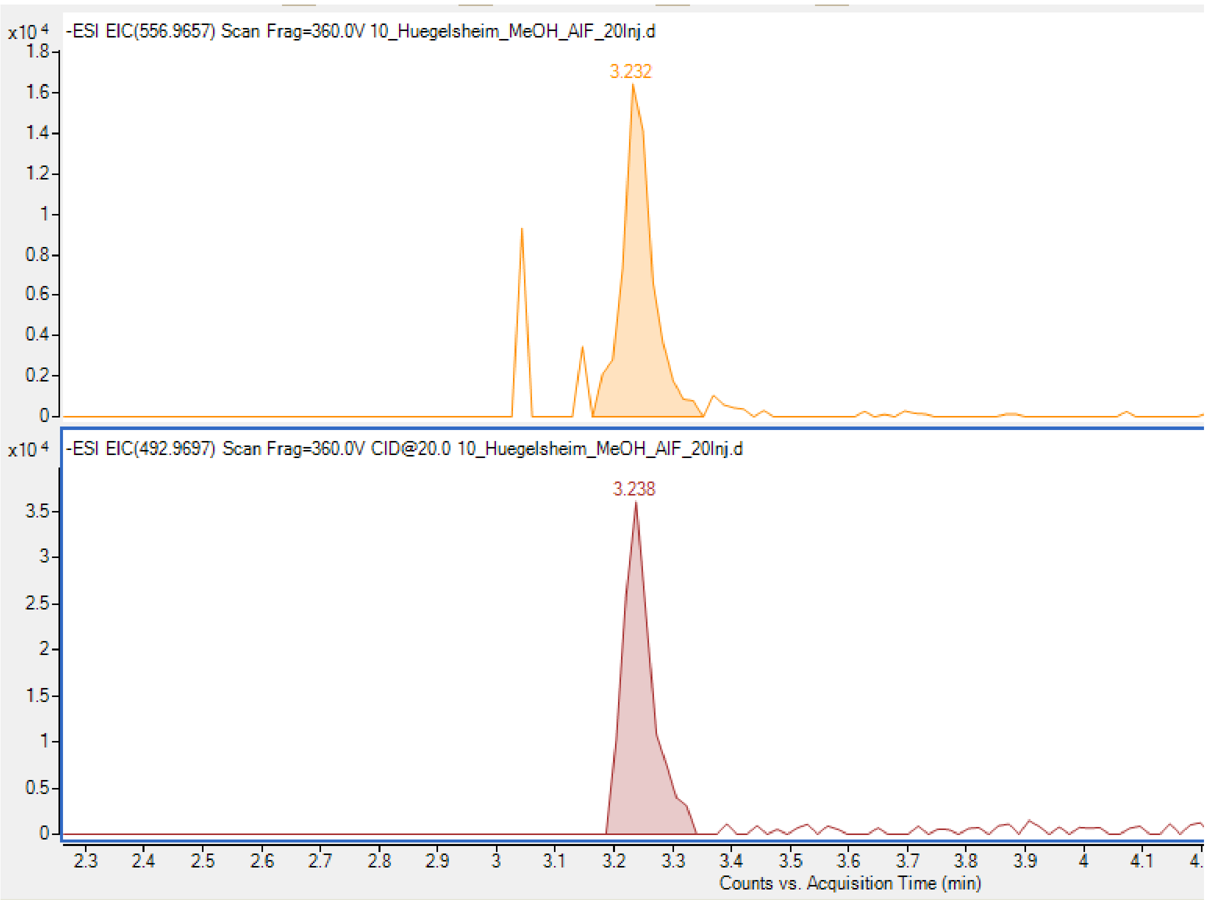

Fig. S1.12 Top: EIC of 556.9657 (m/z of 10:2 FTUCA, 0 eV); bottom: EIC of 492.9697 (m/z of 10:2 FTUCA minus CO2HF, 20 eV)

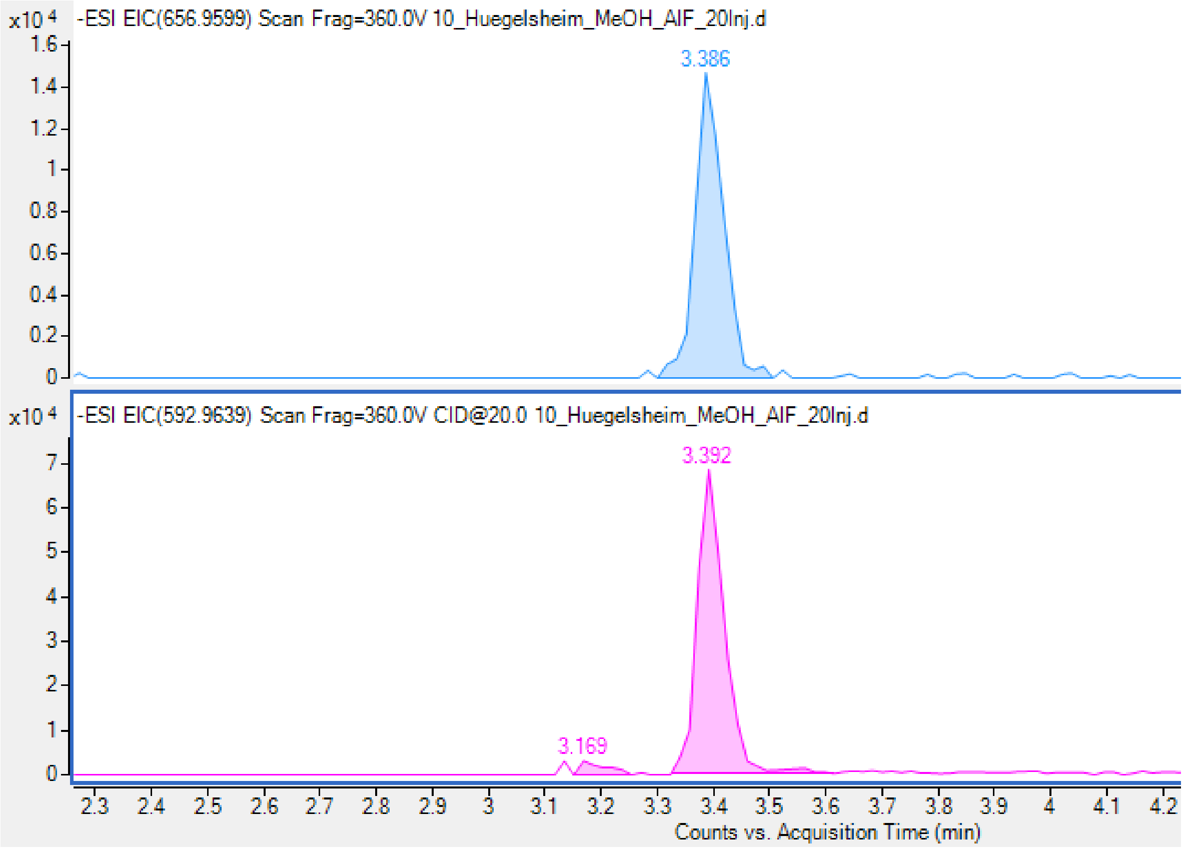

Fig. S1.13 Top: EIC of 656.9599 (m/z of 12:2 FTUCA, 0 eV); bottom: EIC of 592.9639 (m/z of 12:2 FTUCA minus CO2HF, 20 eV)

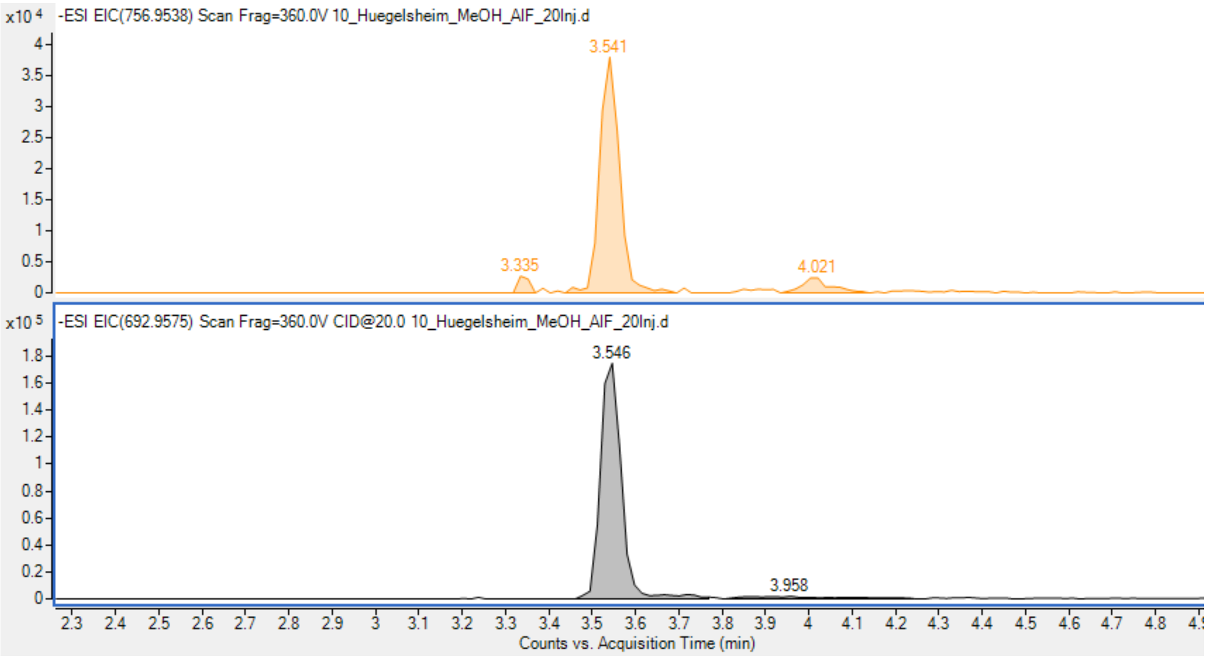

Fig. S1.14 Top: EIC of 756.9538 (m/z of 14:2 FTUCA, 0eV); bottom: EIC of 592.9639 (m/z of 14:2 FTUCA minus CO2HF, 20 eV)

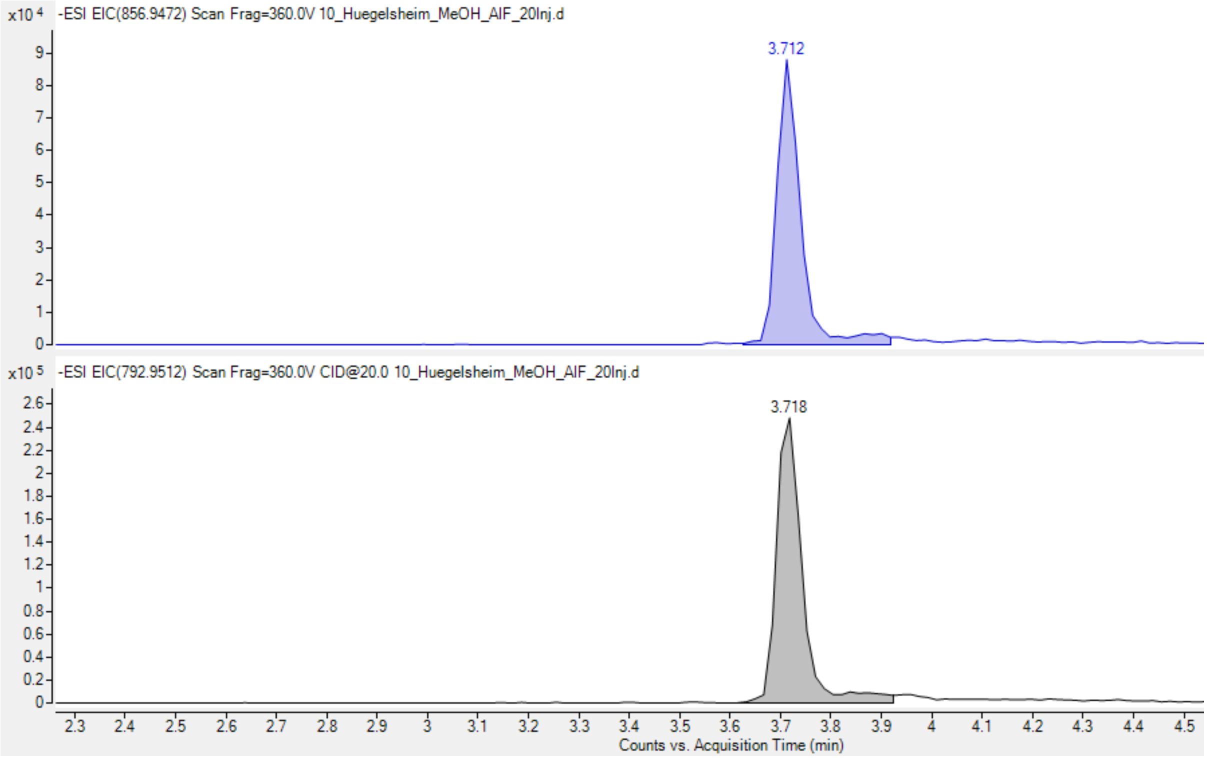

Fig. S1.15 Top: EIC of 856.9472 (m/z of 16:2 FTUCA, 0eV); bottom: EIC of 792.9512 (m/z of 16:2 FTUCA minus CO2HF, 20 eV)

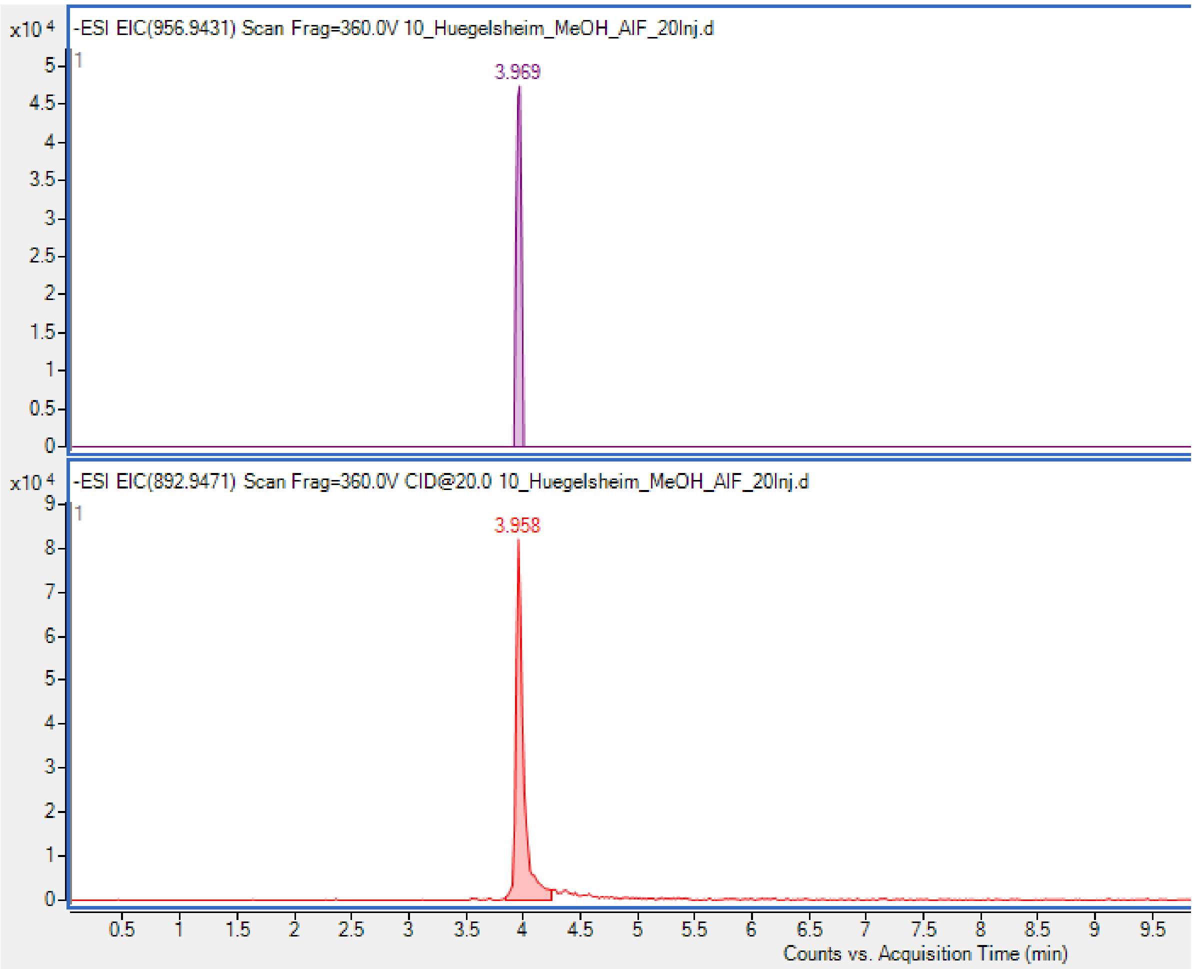

Fig. S1.16 Top: EIC of 956.9431 (m/z of 18:2 FTUCA, 0eV); bottom: EIC of 892.9471 (m/z of 18:2 FTUCA minus CO2HF, 20 eV)

Sample 2: Confirmation of the identification with an authentic standard

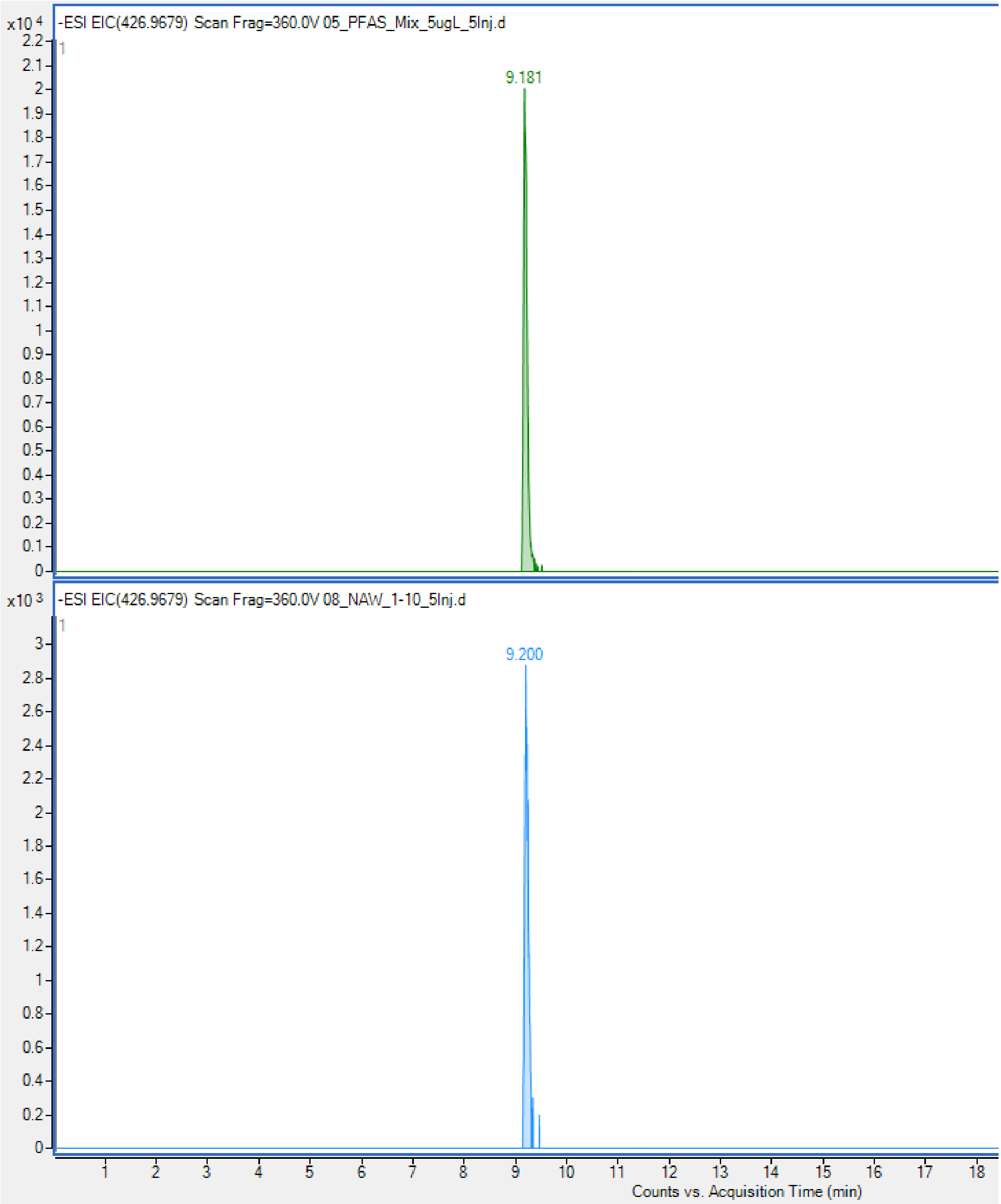

Fig. S1.17 Top: EIC of 6:2 FTS standard 5 µg/L, bottom: 1/10 dilution of sample 2

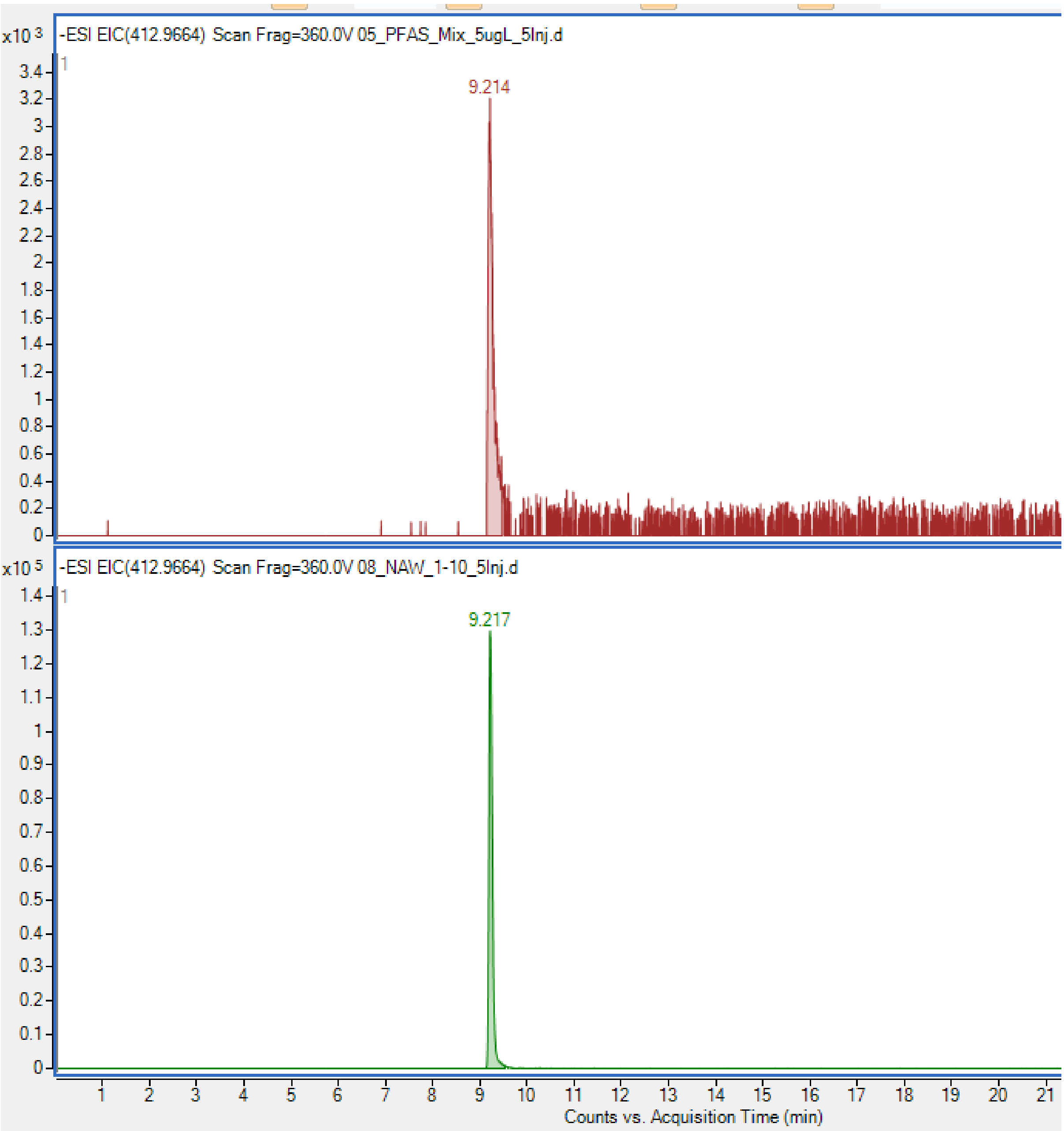

Fig. S1.18 Top: EIC of PFOA standard 5 µg/L, bottom: 1/10 dilution of sample 2

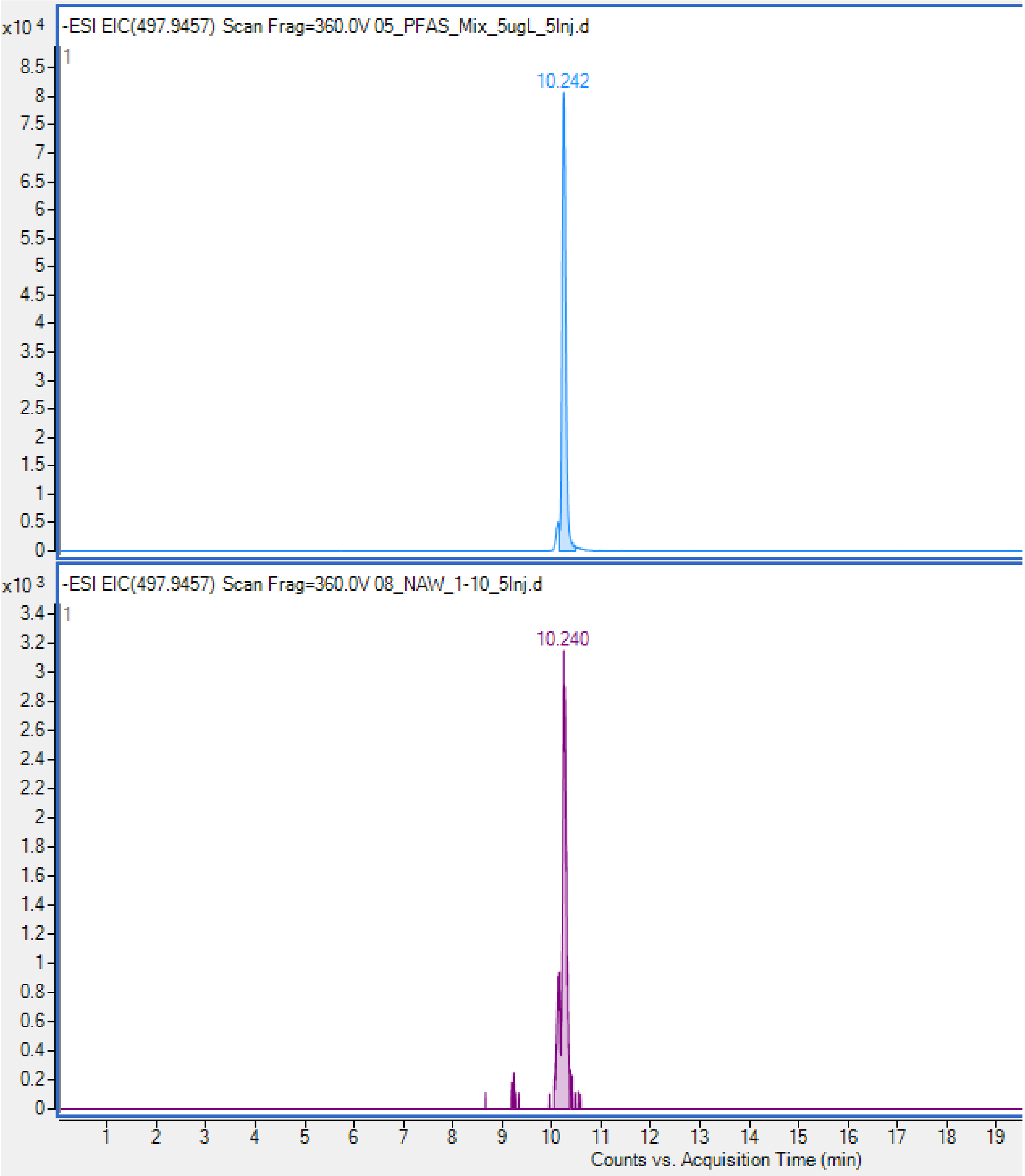

Fig. S1.19 Top: EIC of FOSA standard 5 µg/L, bottom: 1/10 dilution of sample 2

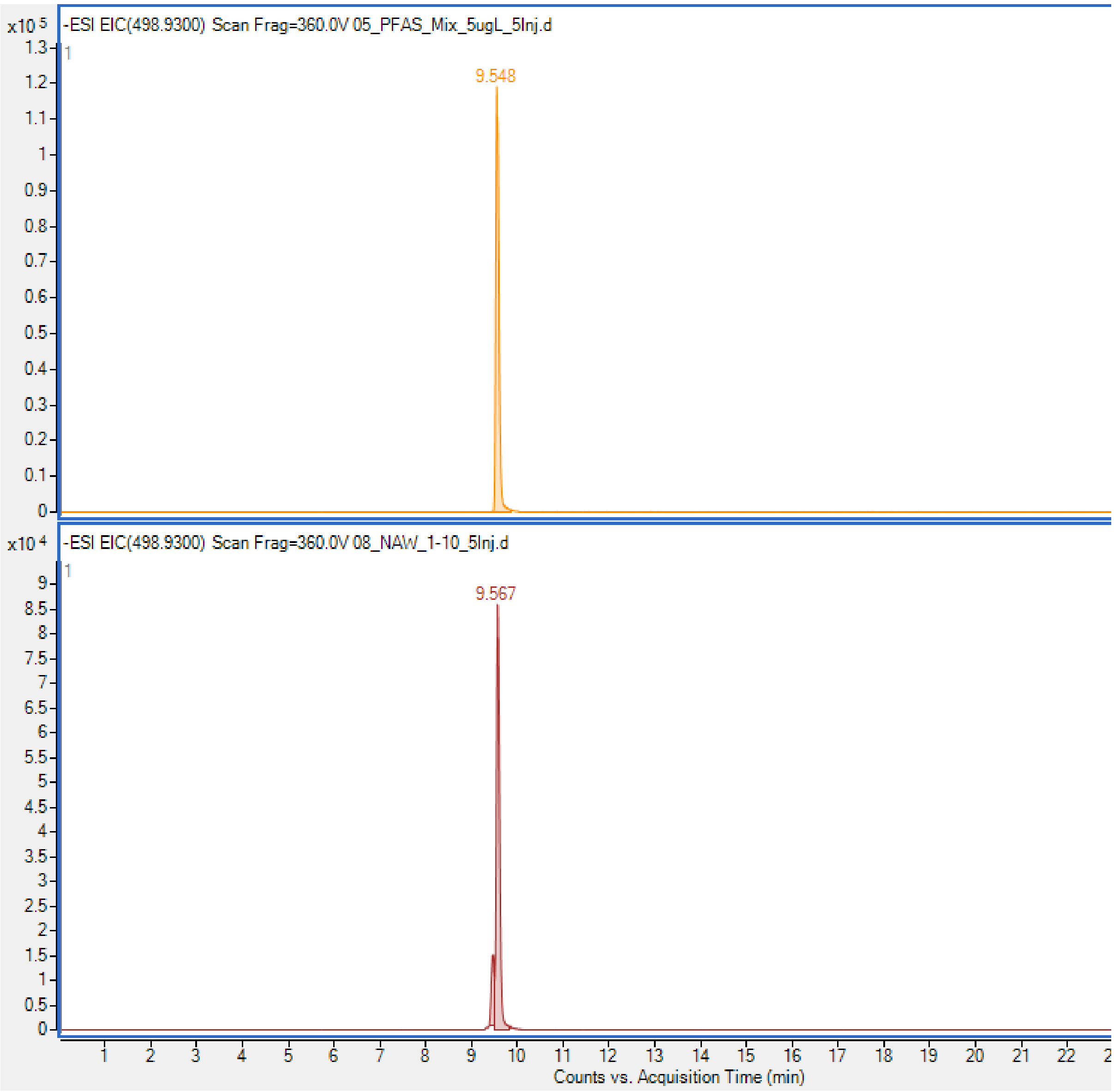

Fig. S1.20 Top: EIC of PFOS standard 5 µg/L, bottom: 1/10 dilution of sample 2

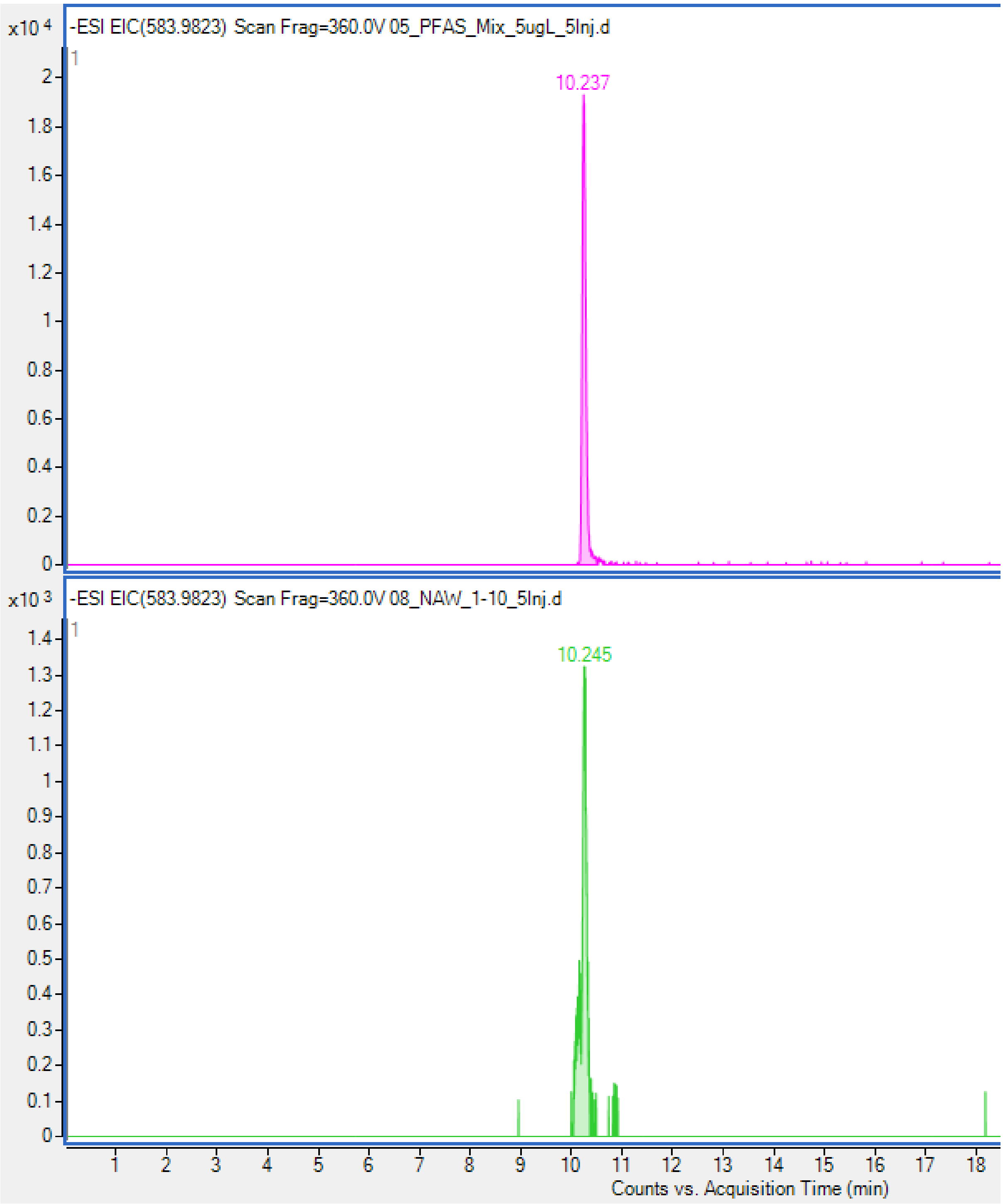

Fig. S1.21 Top: EIC of EtFOSAA standard 5 µg/L, bottom: 1/10 dilution of sample 2

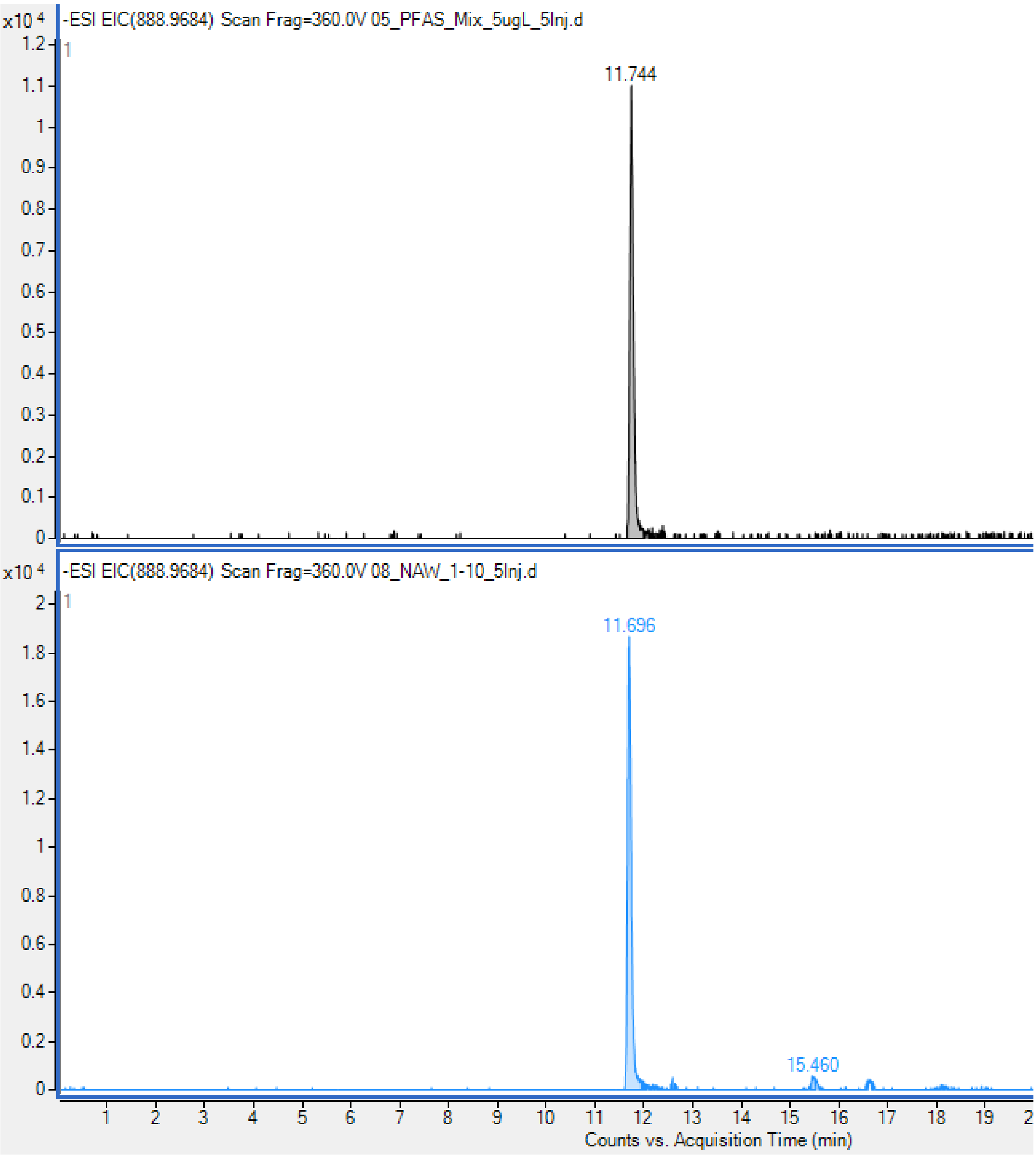

Fig. S1.22 Top: EIC of 6:2 diPAP standard 5 µg/L, bottom: 1/10 dilution of sample 2

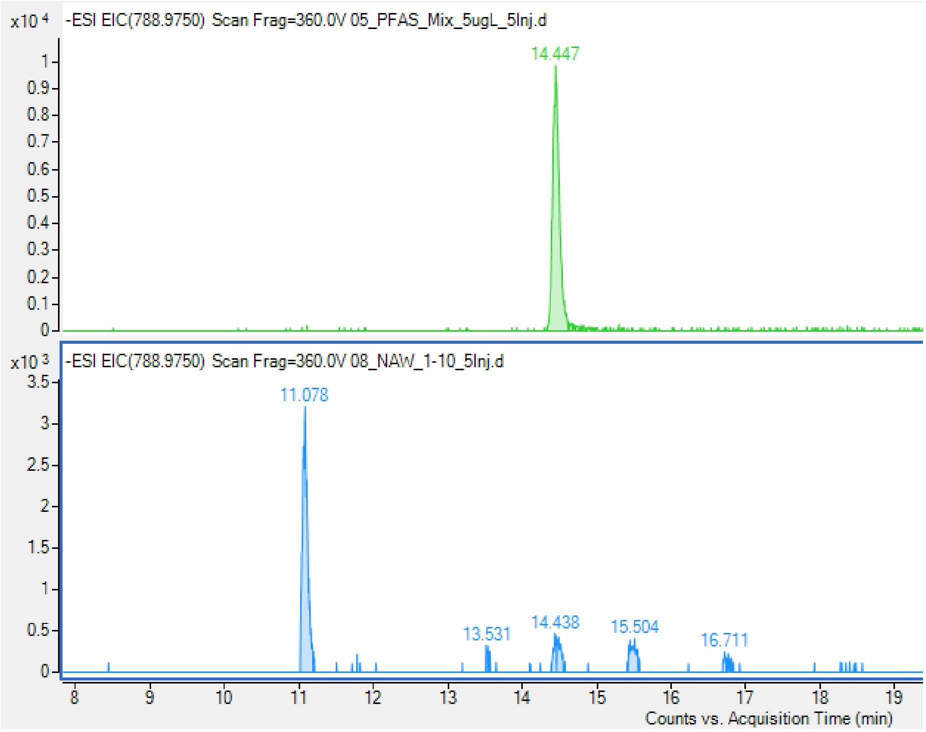

Fig. S1.22b Top: EIC of triPAP standard 5µg/L, bottom: 1/10 dilution of sample 2

Sample 3: Confirmation of the identification with an authentic standard

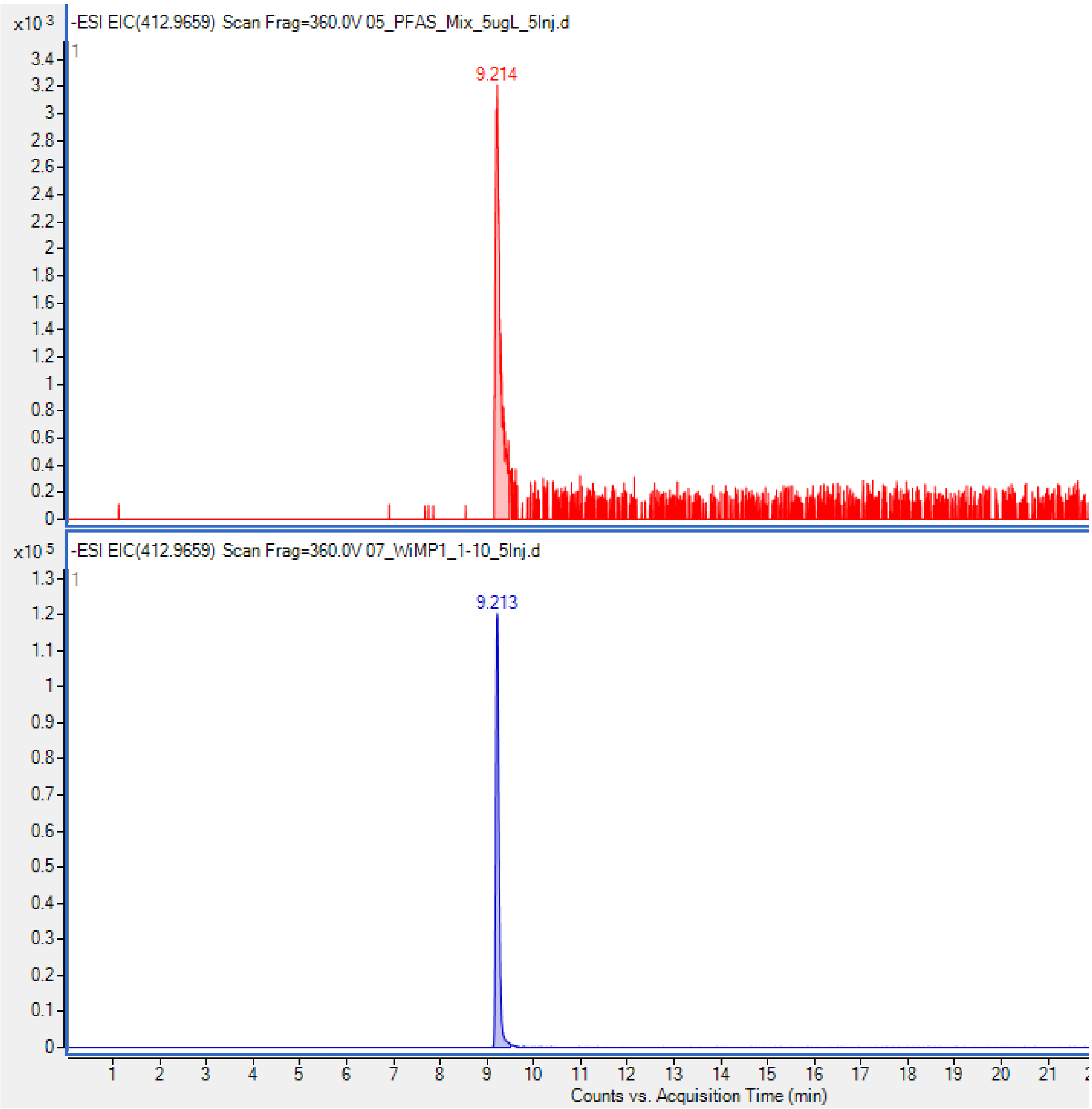

Fig. S1.23 Top: EIC of PFOA standard 5 µg/L, bottom: 1/10 dilution of sample 3

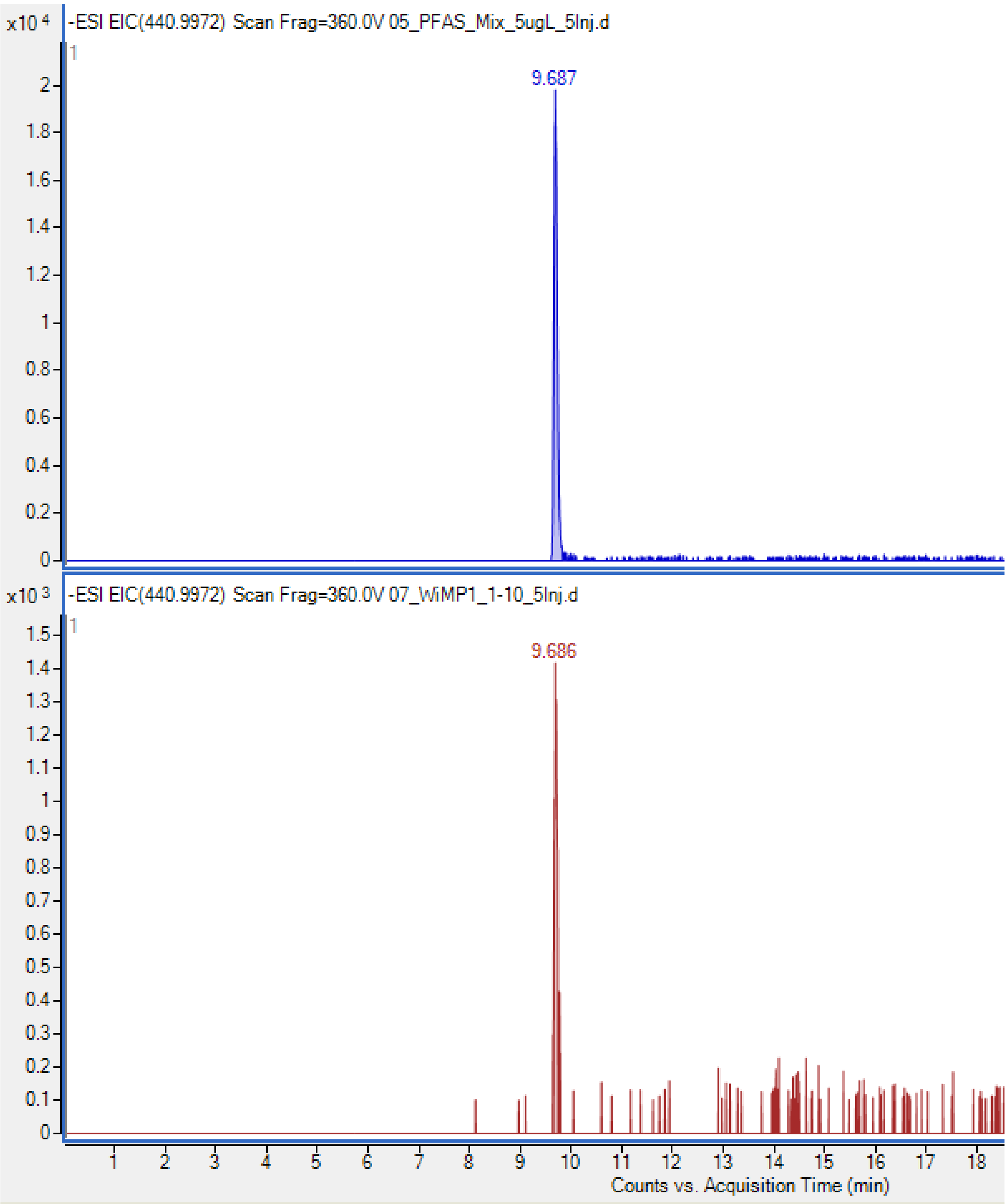

Fig. S1.24 Top: EIC of 7:3 PFCA standard 5 µg/L, bottom: 1/10 dilution of sample 3

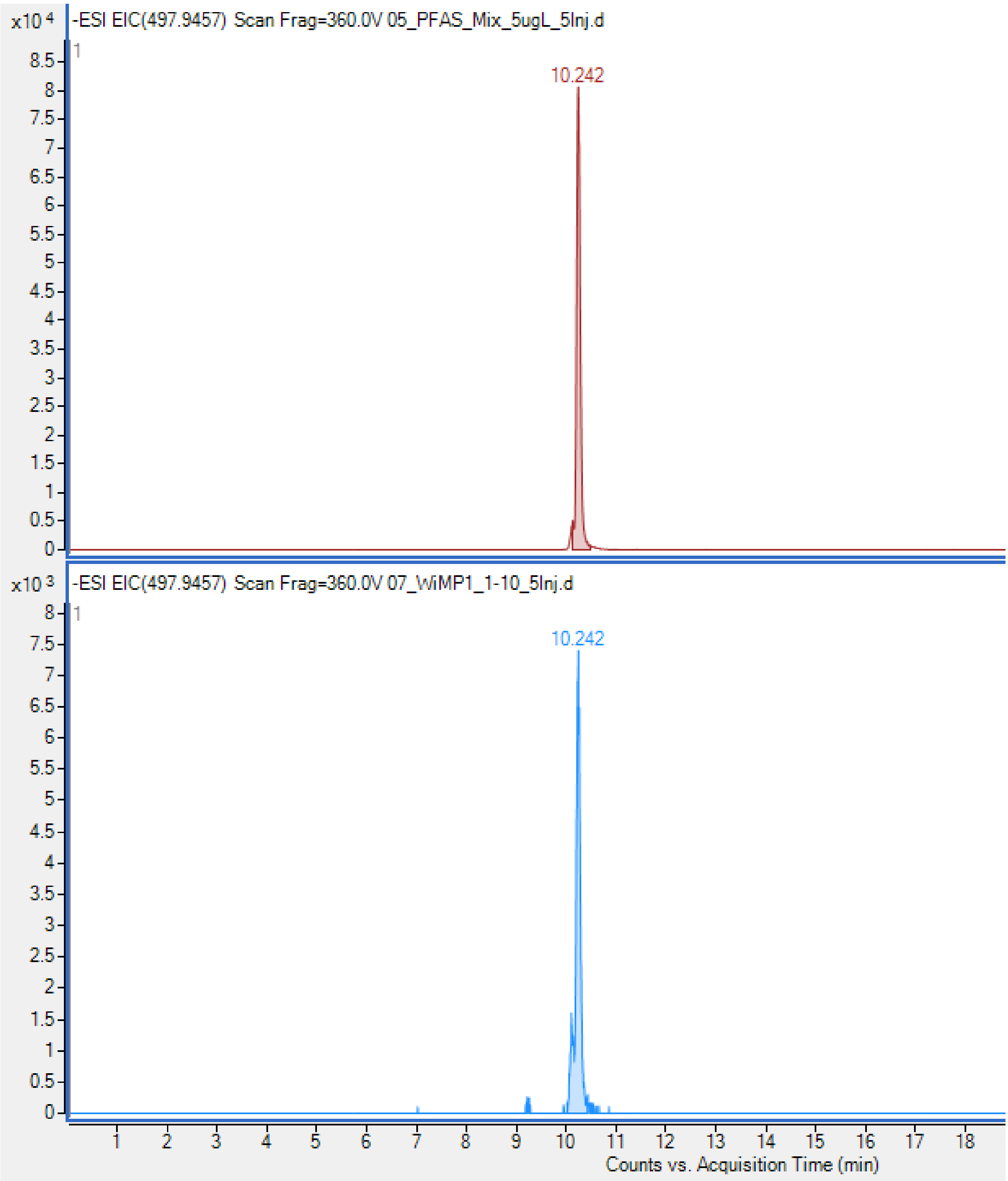

Fig. S1.25 Top: EIC of FOSA standard 5 µg/L, bottom: 1/10 dilution of sample 3

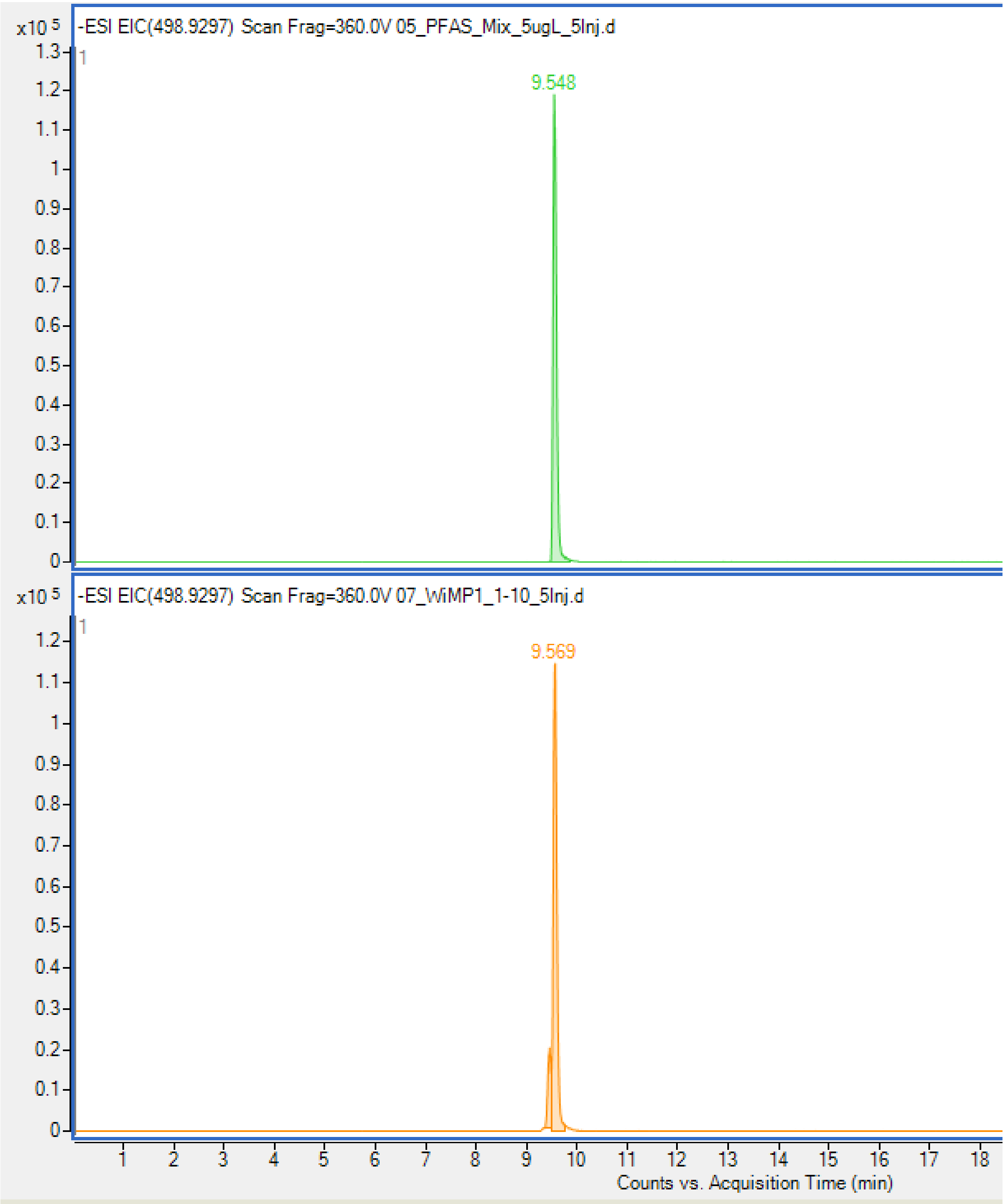

Fig. S1.26 Top: EIC of PFOS standard 5 µg/L, bottom: 1/10 dilution of sample 3

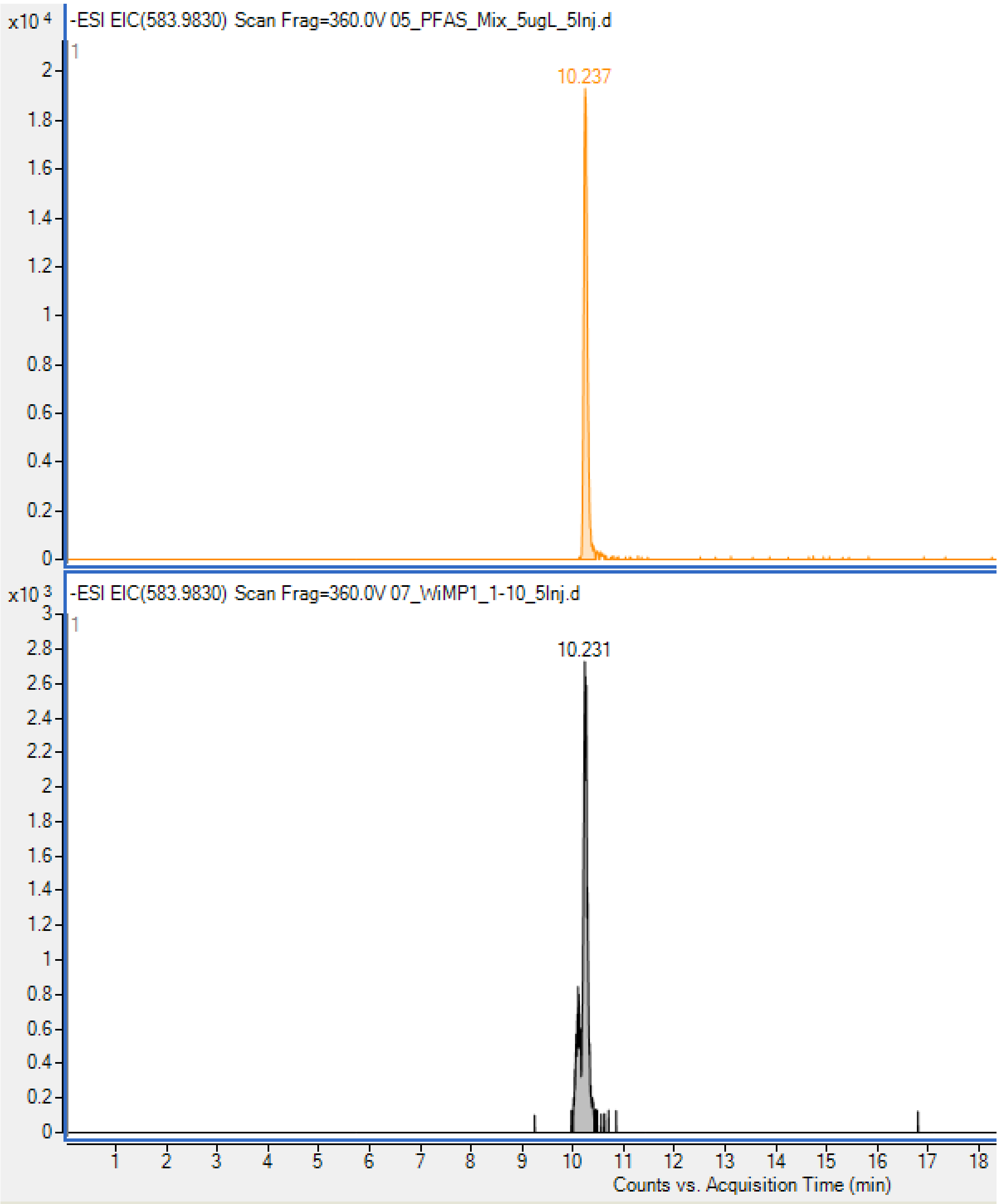

Fig. S1.27 Top: EIC of EtFOSAA standard 5 µg/L, bottom: 1/10 dilution of sample 3

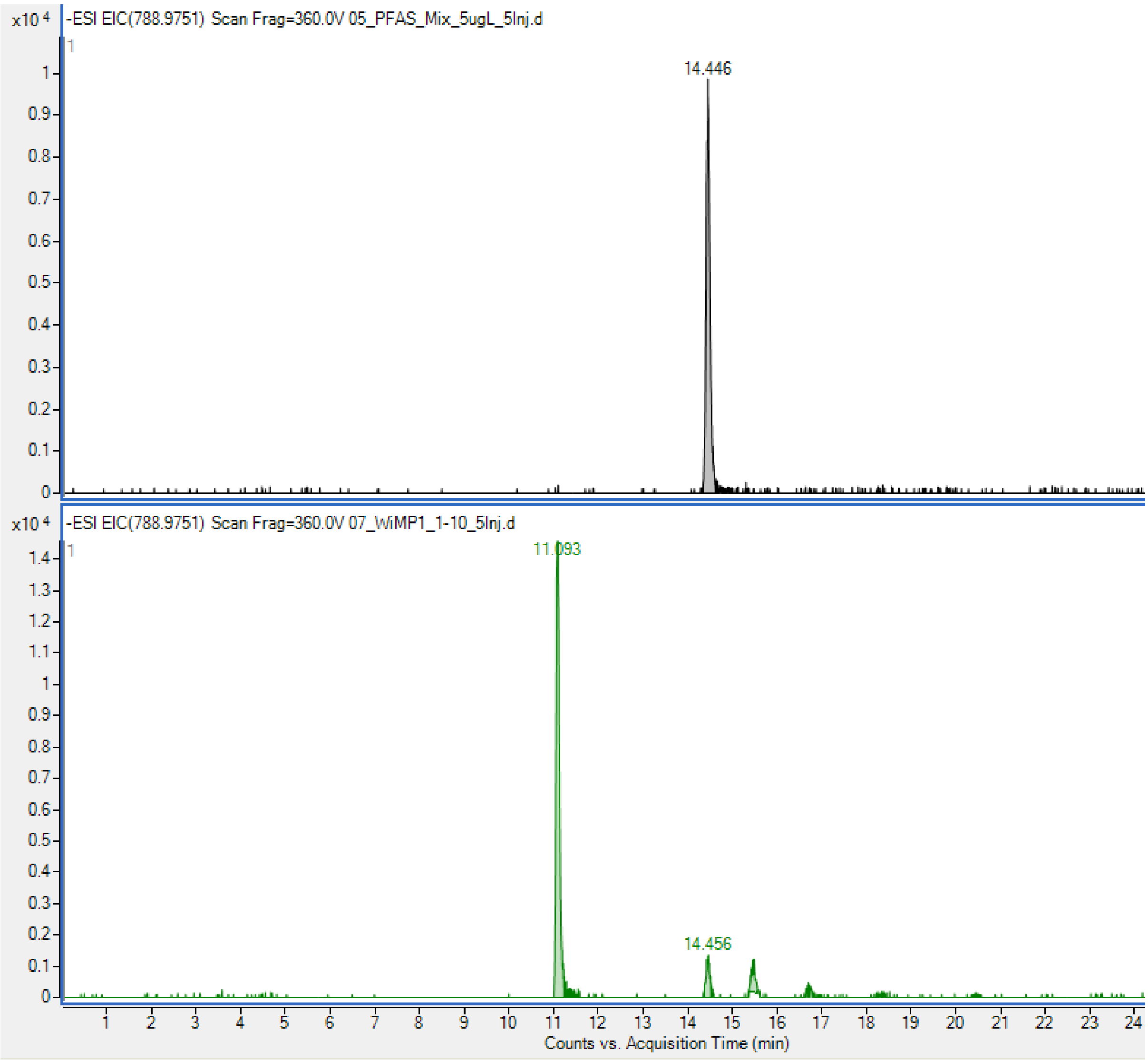

Fig. S1.28 Top: EIC of 6:2 triPAP standard 5 µg/L, bottom: 1/10 dilution of sample 3

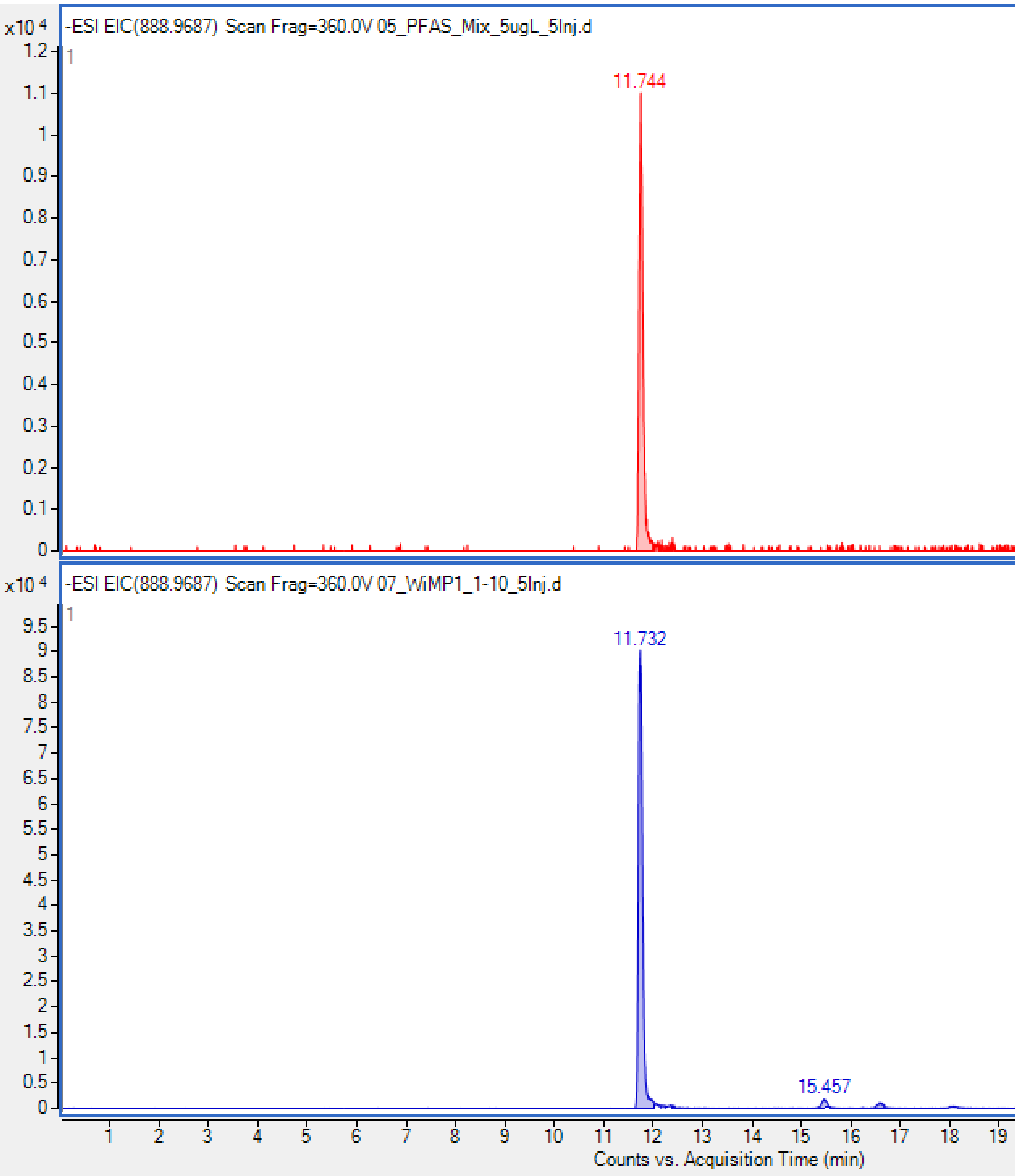

Fig. S1.29 Top: EIC of 6:2 / 8:2 diPAP standard 5 µg/L, bottom: 1/10 dilution of sample 3

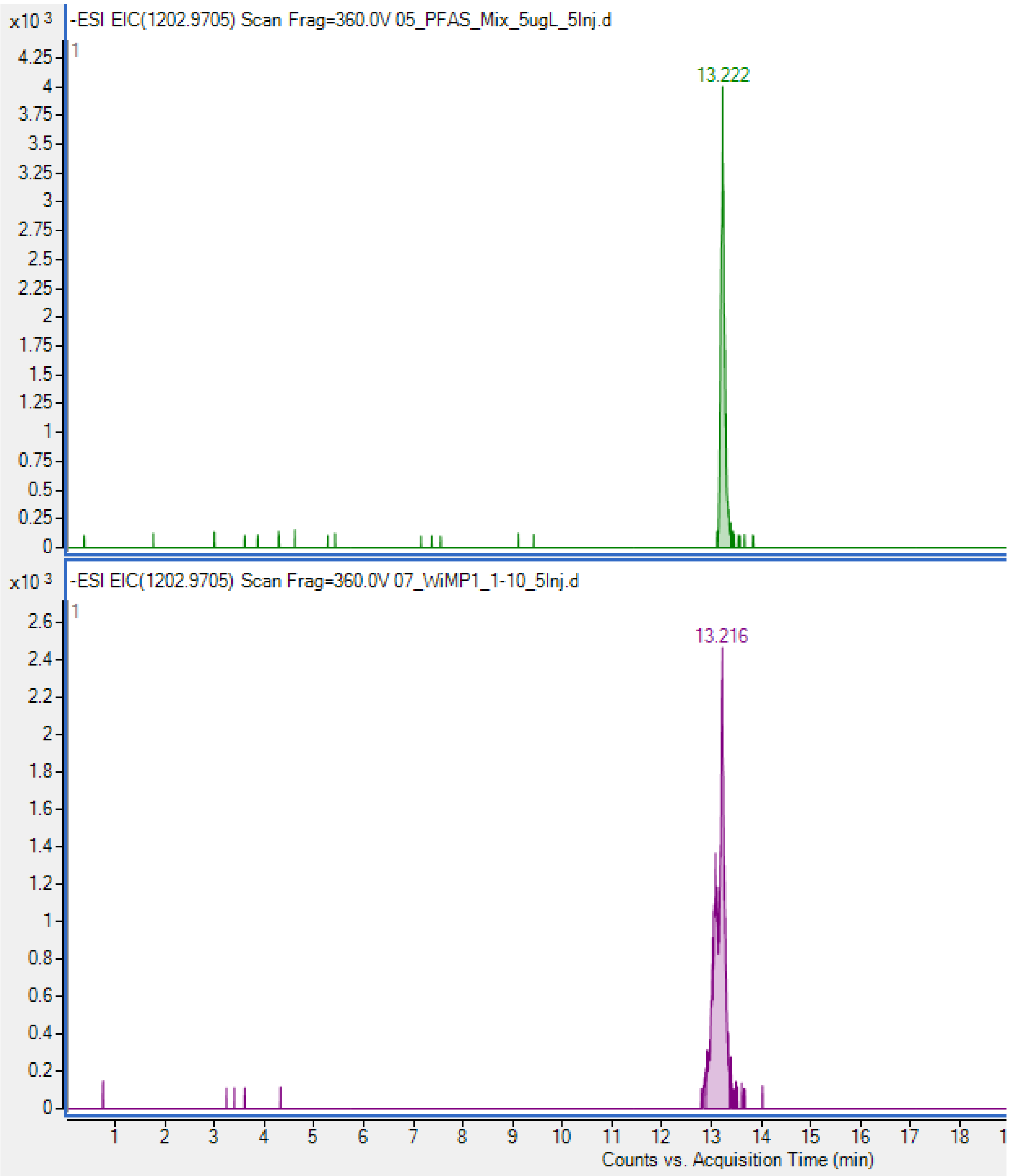

Fig. S1.30 Top: EIC of diSAmPAP standard 5 µg/L, bottom: 1/10 dilution of sample 3

Sample 4 Confirmation of the identification with an authentic standard

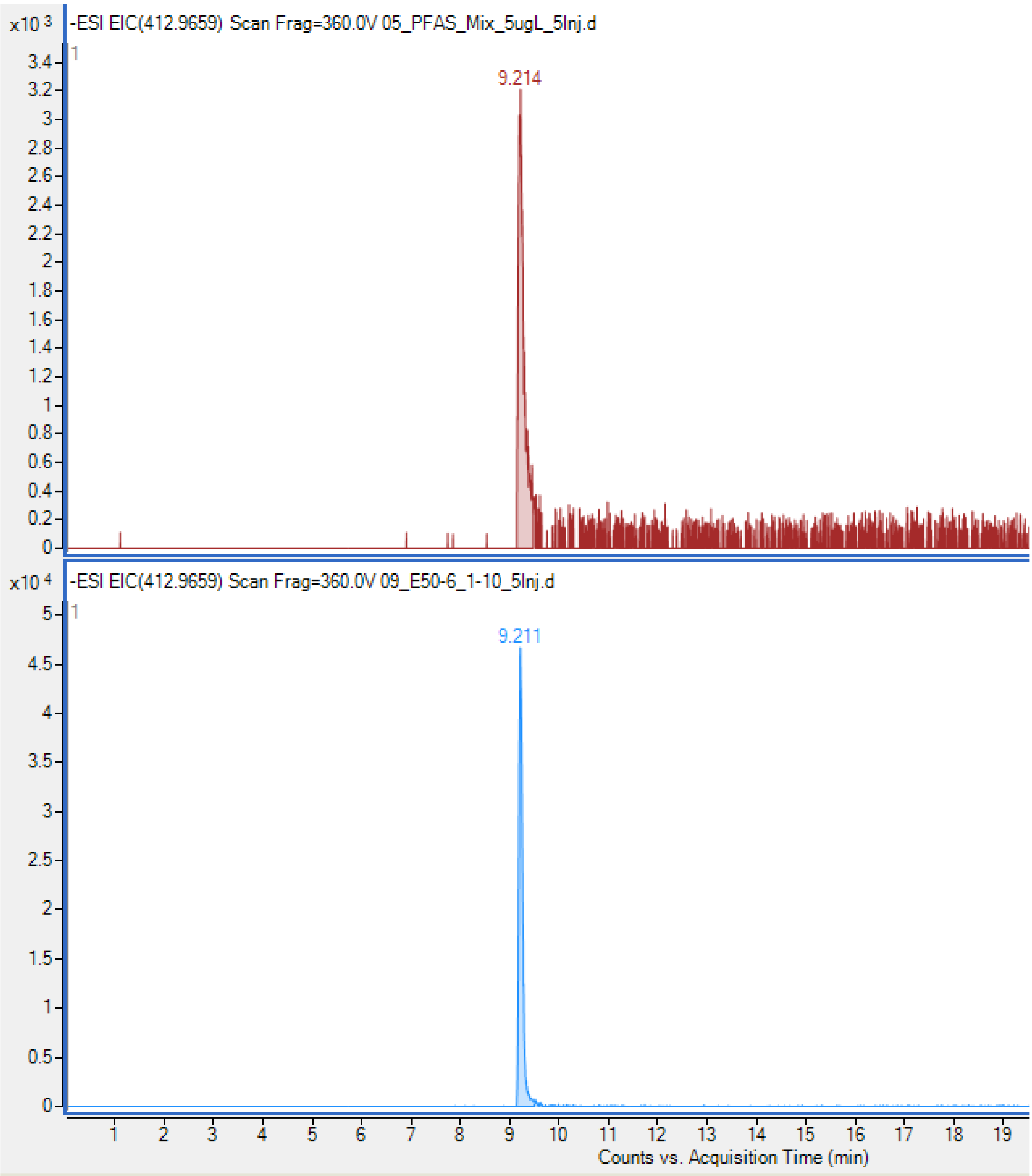

Fig. S1.31 Top: EIC of PFOA standard 5 µg/L, bottom: 1/10 dilution of sample 4

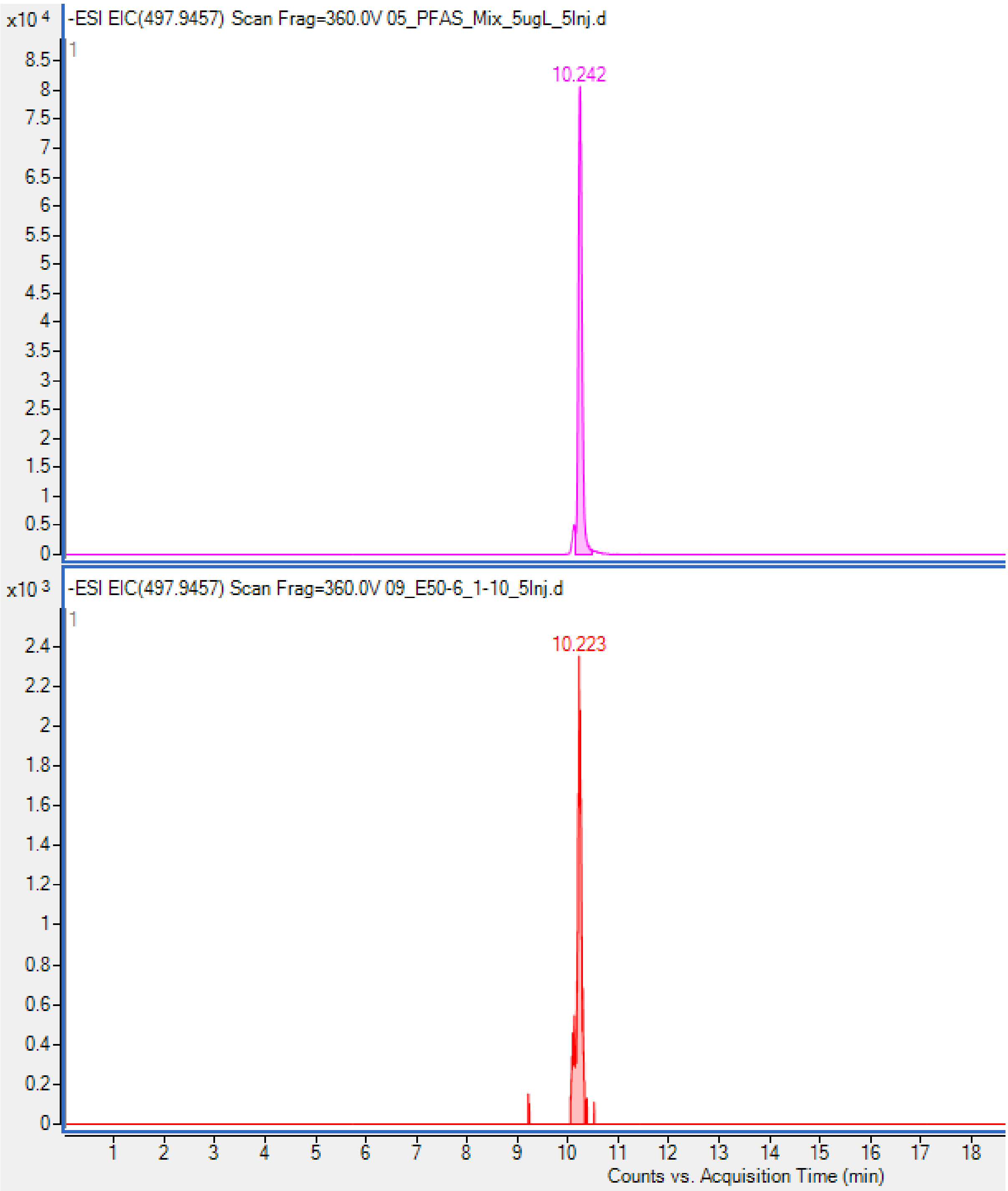

Fig. S1.32 Top: EIC of FOSA standard 5 µg/L, bottom: 1/10 dilution of sample 4

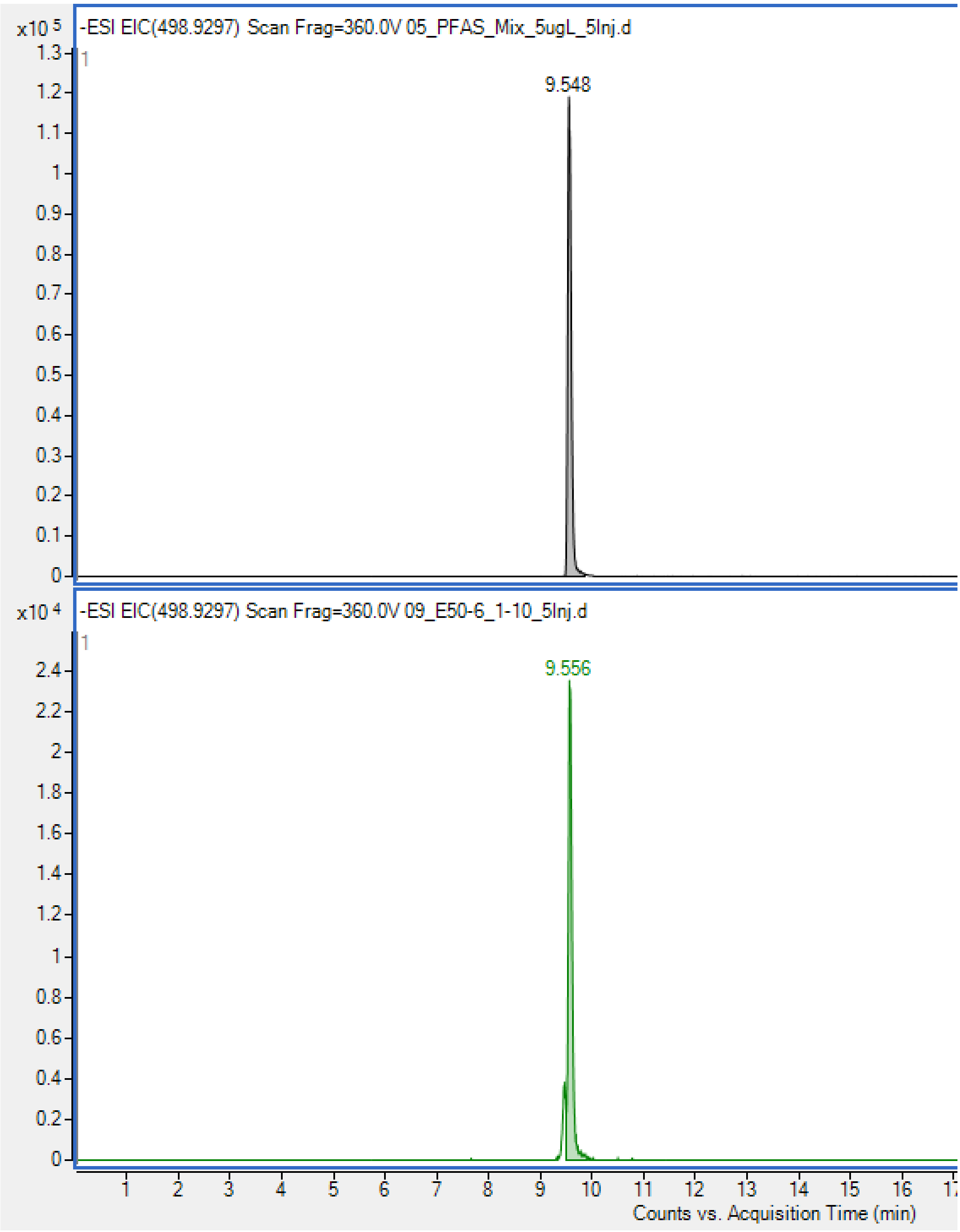

Fig. S1.33 Top: EIC of PFOS standard 5 µg/L, bottom: 1/10 dilution of sample 4

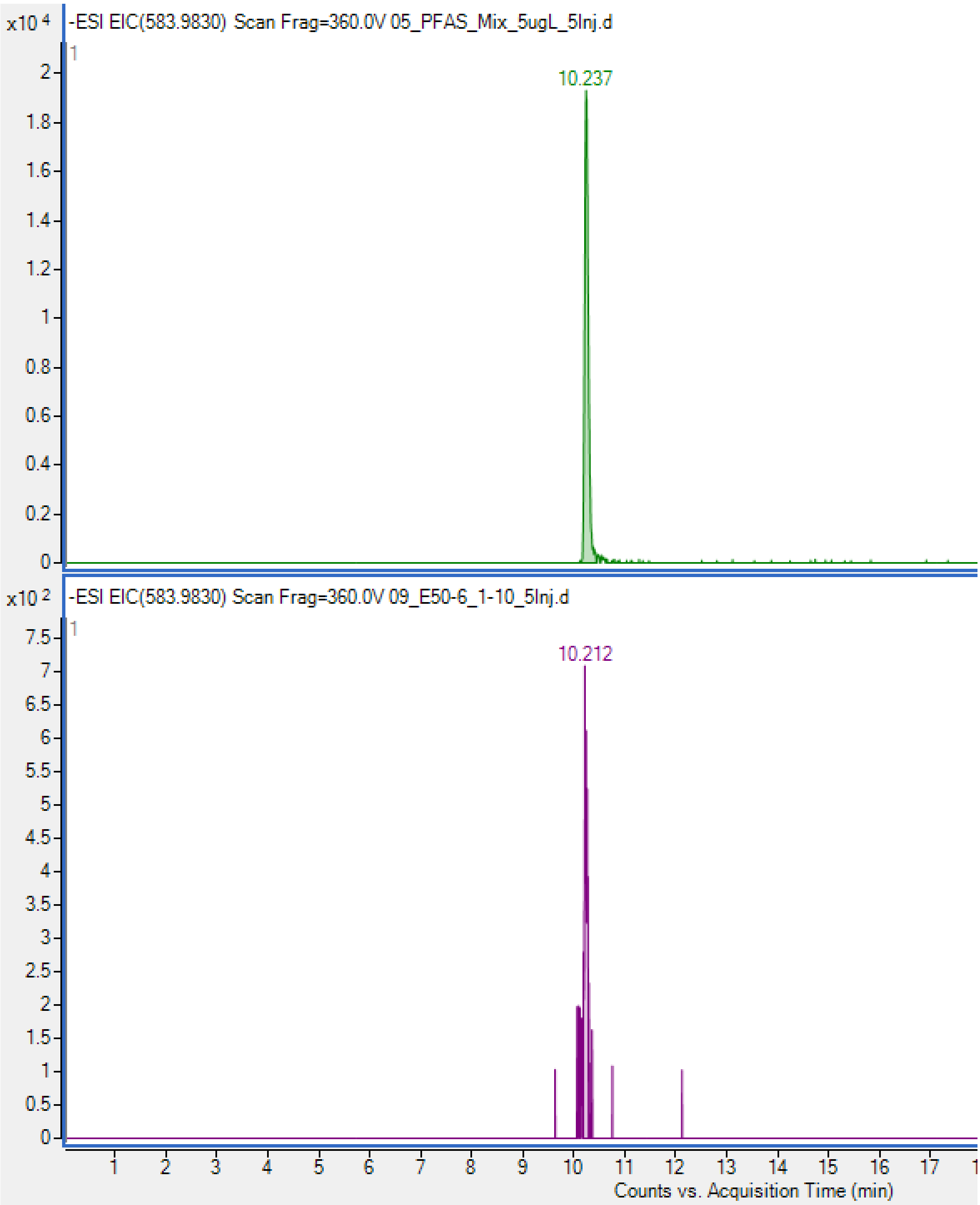

Fig. S1.34 Top: EIC of EtFOSAA standard 5 µg/L, bottom: 1/10 dilution of sample 4

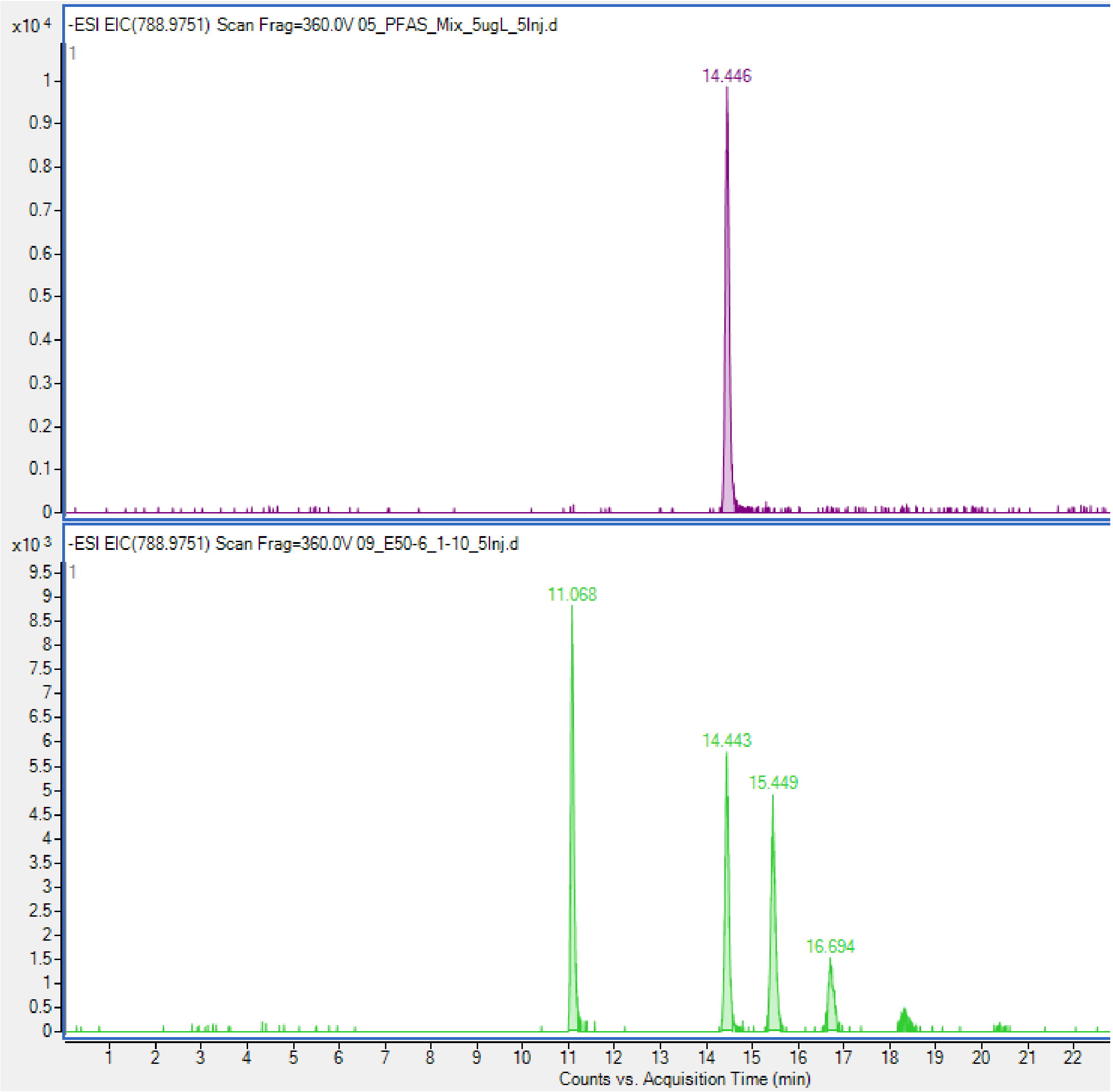

Fig. S1.35 Top: EIC of 6:2 triPAP standard 5 µg/L, bottom: 1/10 dilution of sample 4

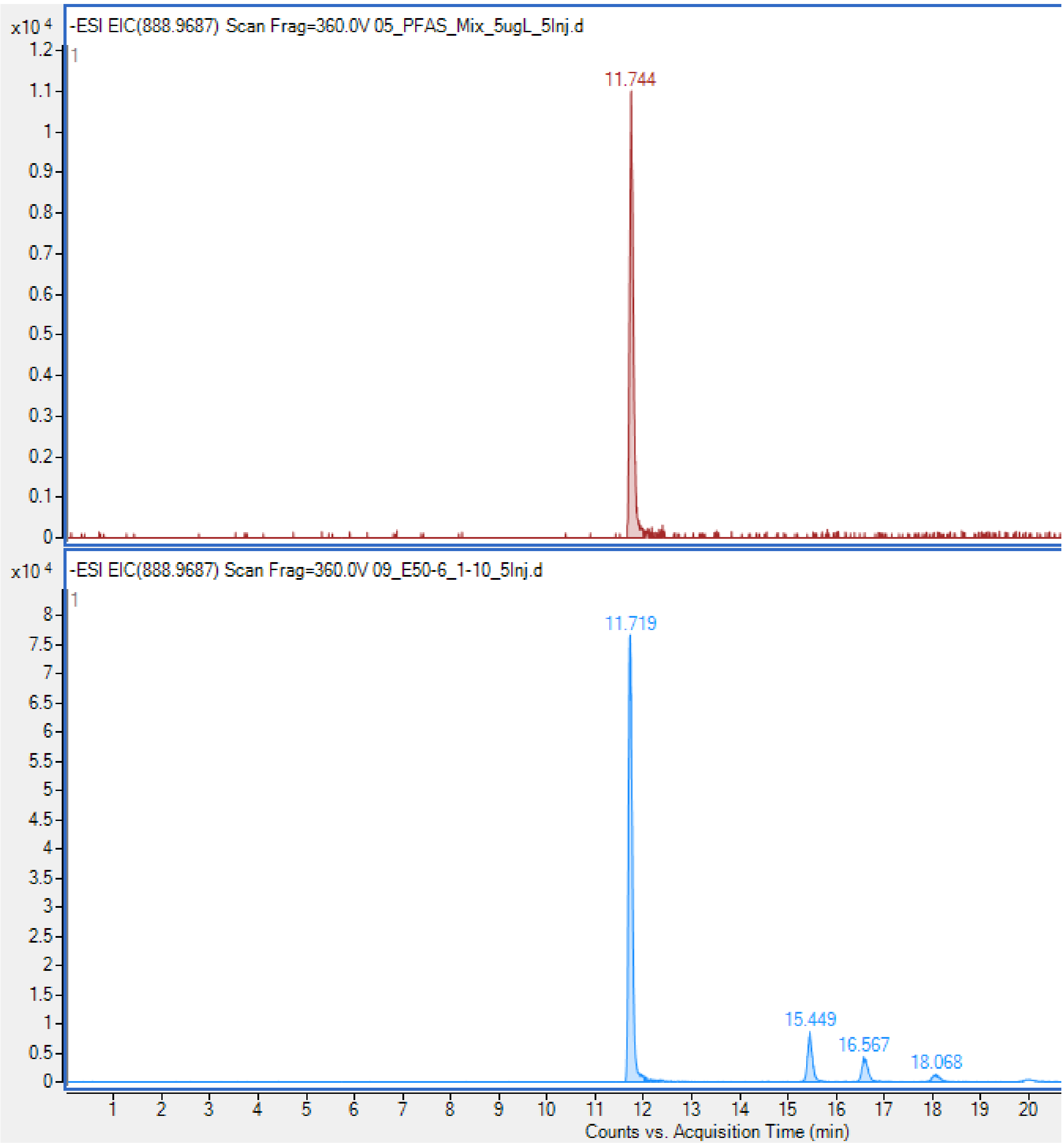

Fig. S1.36 Top: EIC of 6:2 / 8:2 diPAP standard 5 µg/L, bottom: 1/10 dilution of sample 4

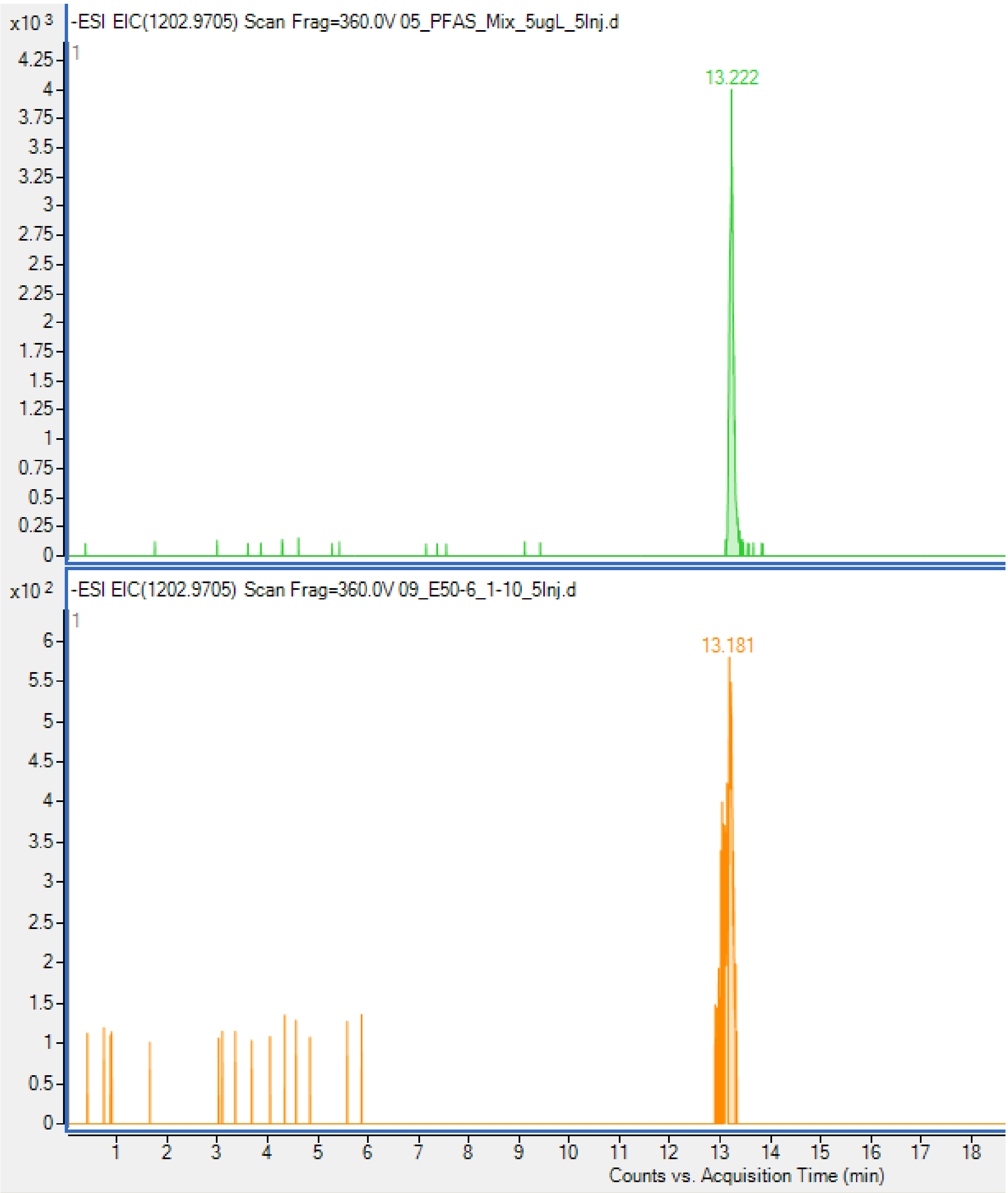

Fig. S1.37 Top: EIC of diSAmPAP standard 5 µg/L, bottom: 1/10 dilution of sample 4

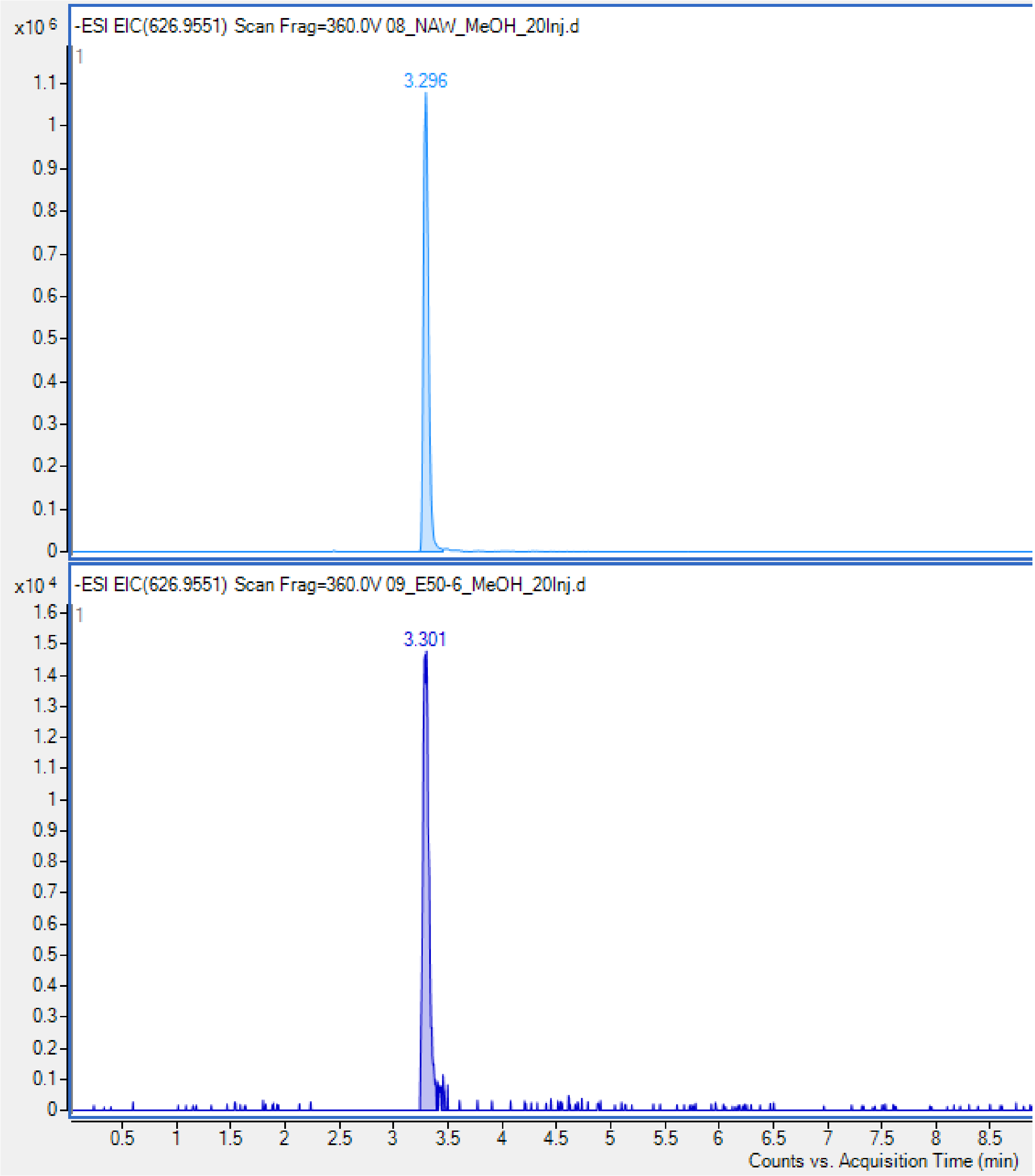

Fig. S1.38 10:2 FTSA RT match with sample 2. Top: Sample 2; bottom: Sample 4

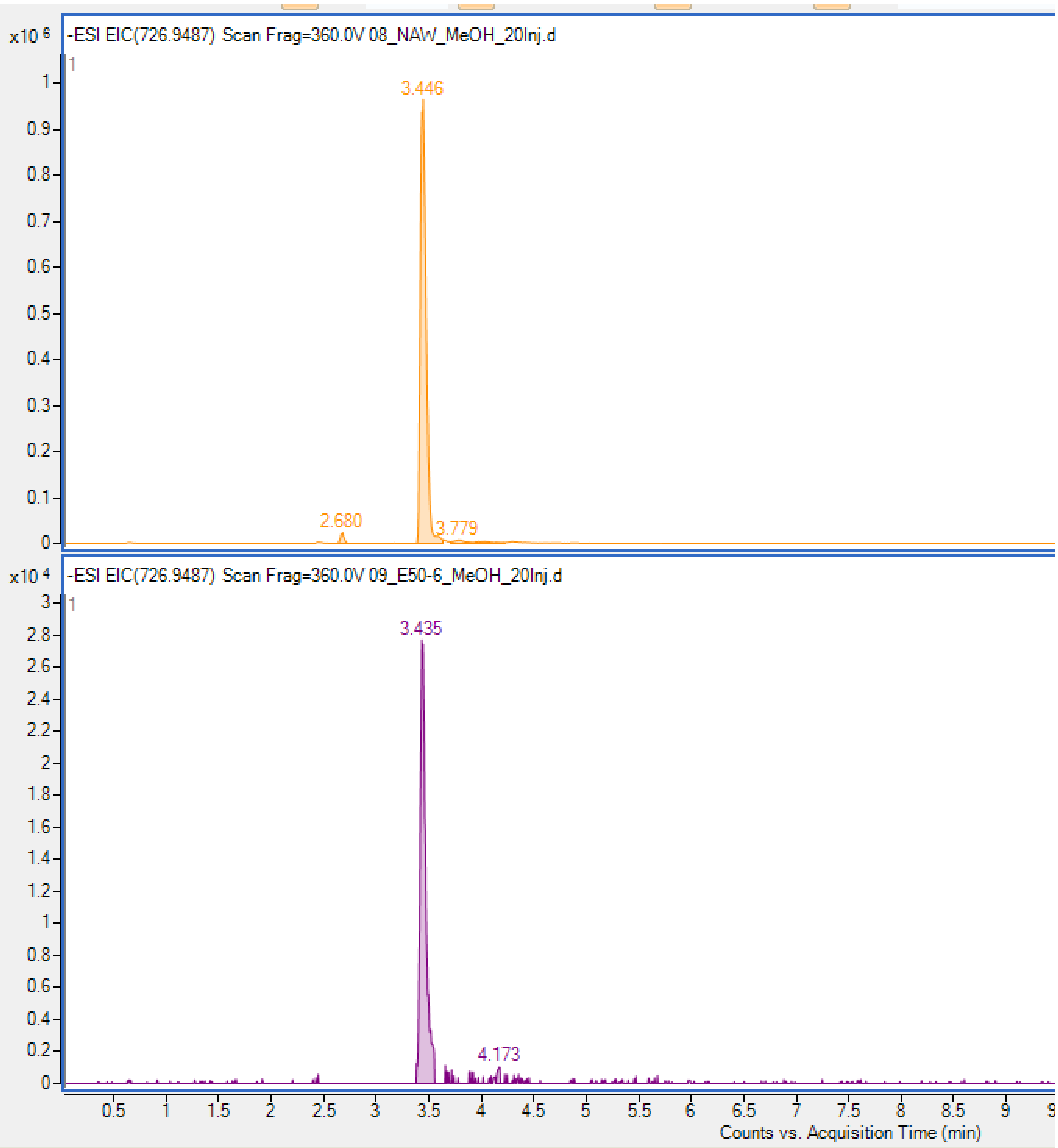

Fig. S1.39 12:2 FTSA RT match with sample 2. Top: Sample 2; bottom: Sample 4

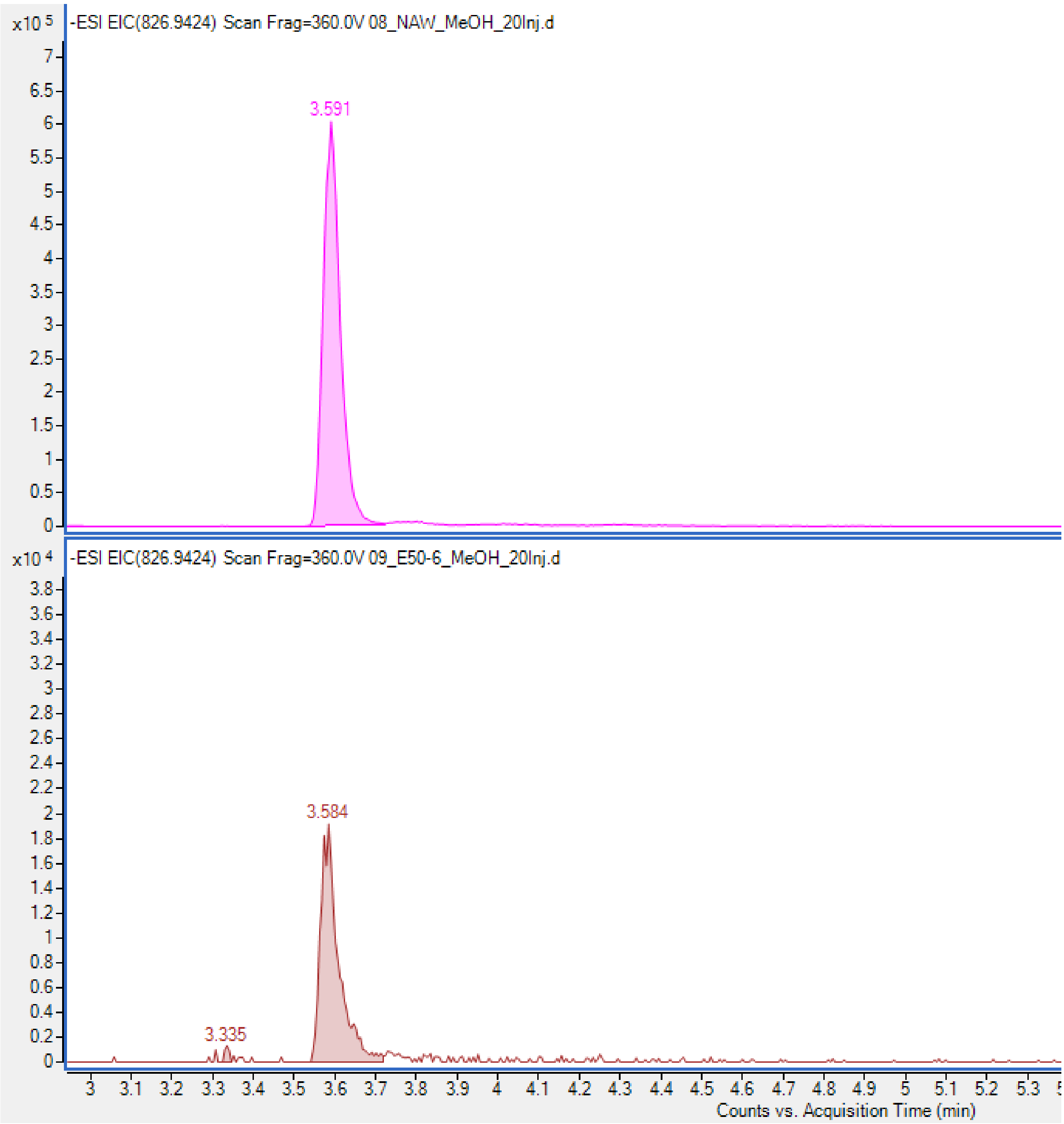

Fig. S1.40 14:2 FTSA RT match with sample 2. Top: Sample 2; bottom: Sample 4

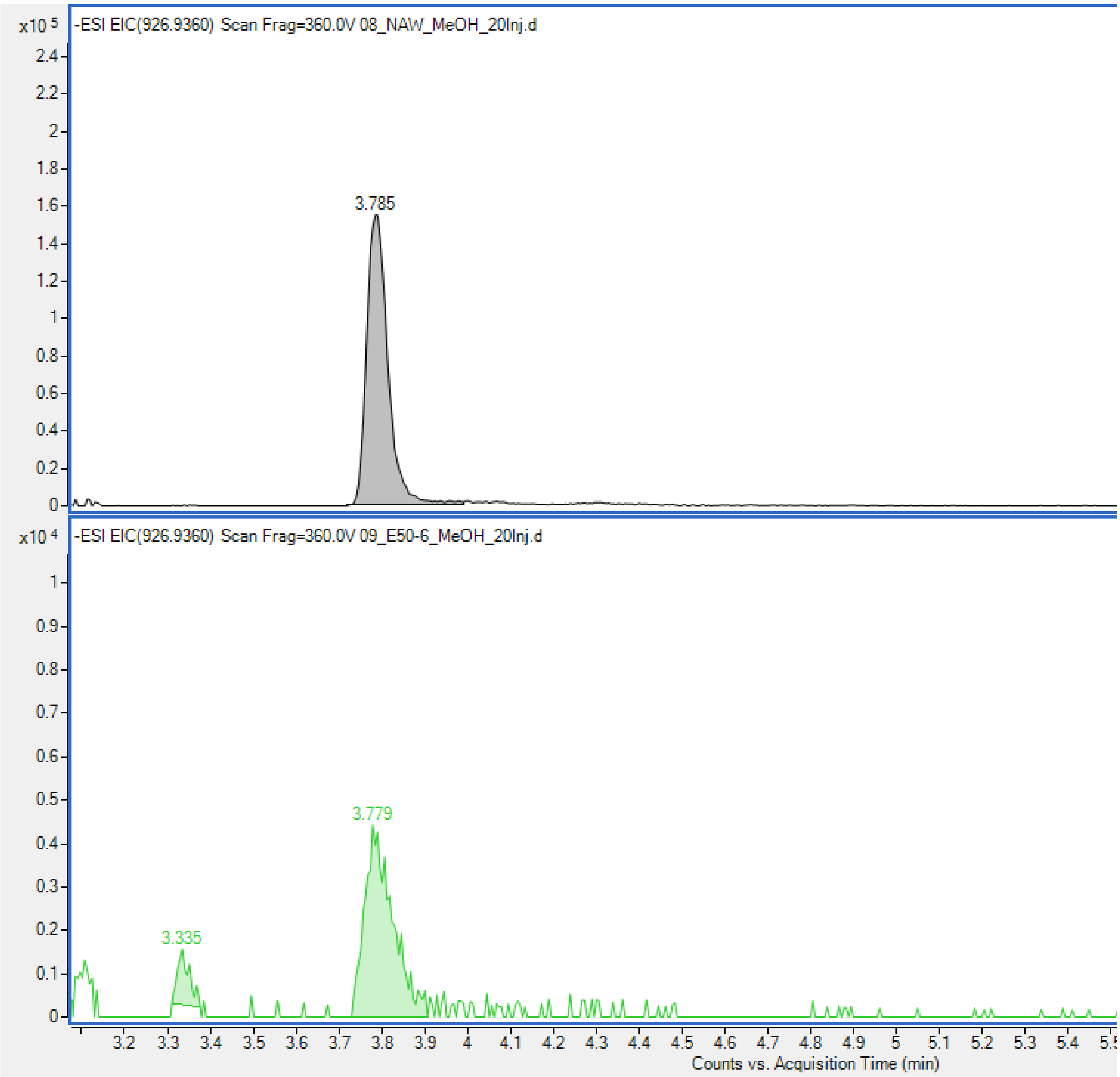

Fig. S1.41 16:2 FTSA RT match with sample 2. Top: Sample 2; bottom: Sample 4

Targeted MS/MS measurements

These measurements were performed on an Agilent 1260 LC coupled to an Agilent 6490 QqQ instrument with following instrumental parameters: Gas temperature: 150 °C, gas flow: 12 L/min, Nebulizer: 45 psi, SheathGasHeater: 380, SheathGasFlow: 12, Capillary: 3000 V, VCharging: 0. The same column was chosen as described in the main method, the temperature was set to 35 °C. 5 µL of the sample were injected. The eluents were chosen as in the main method. The gradient profile started with 15% B, followed by a linear change to 100% B within 10 min, kept isocratic for another 5 min.

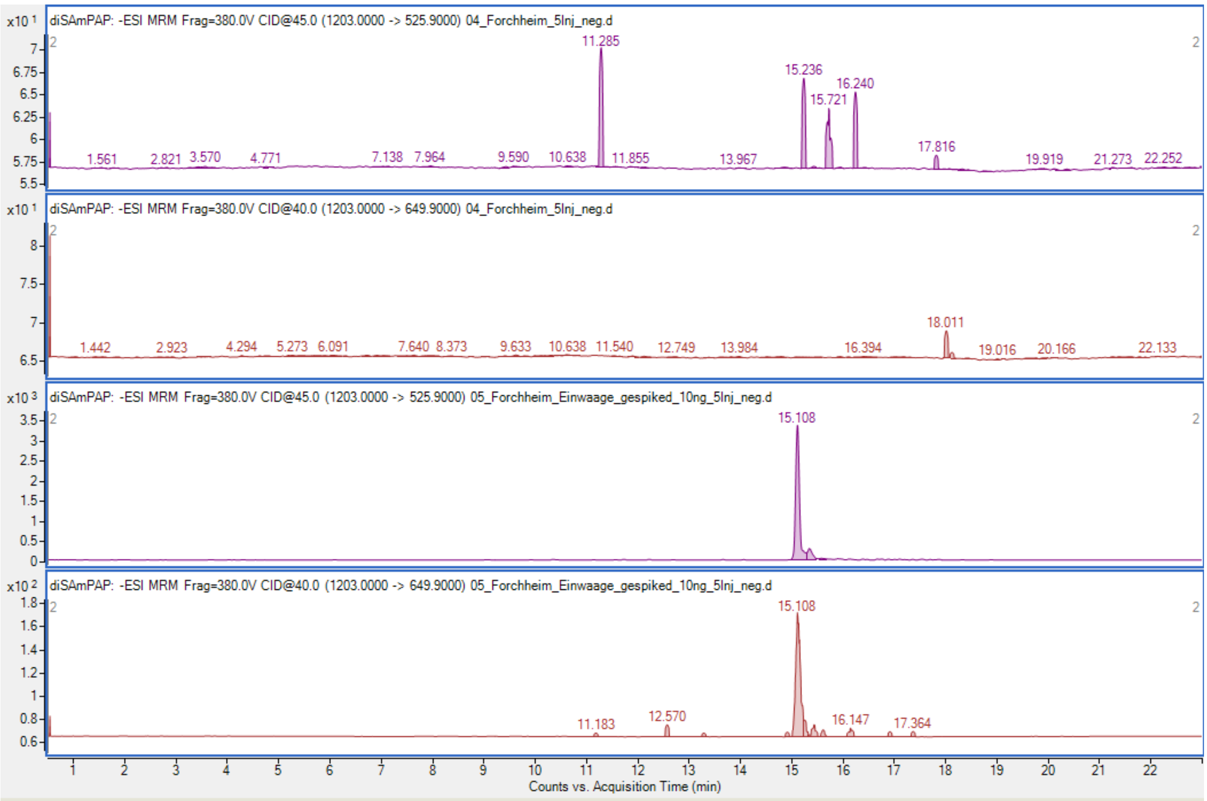

Fig. S1.42 diSAmPAP (1203 -> 525.9, purple; 1203 -> 649.9, red). Top two chromatograms: uncontaminated soil sample, bottom 2 chromatograms: spiked uncontaminated soil sample

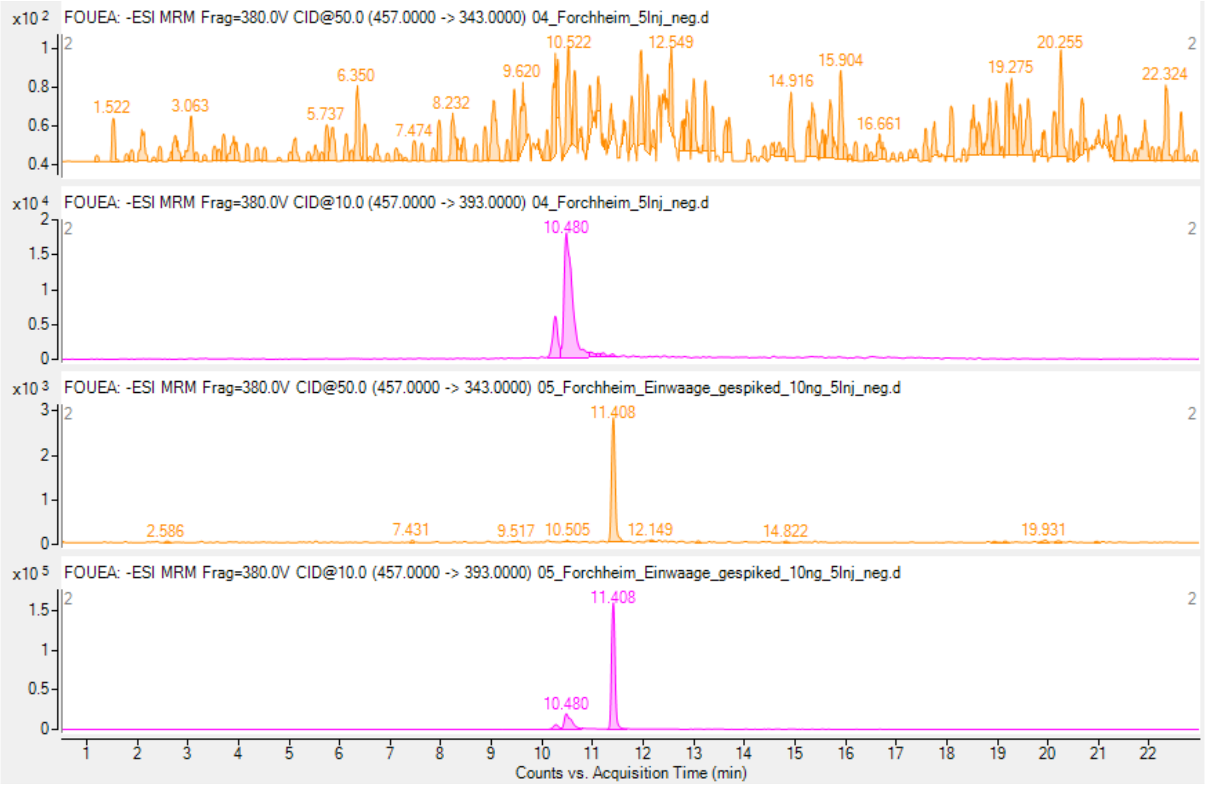

Fig. S1.43 8:2 PFCA (457 -> 343, orange; 457 -> 393, pink). Top two chromatograms: uncontaminated soil sample, bottom 2 chromatograms: spiked uncontaminated soil sample

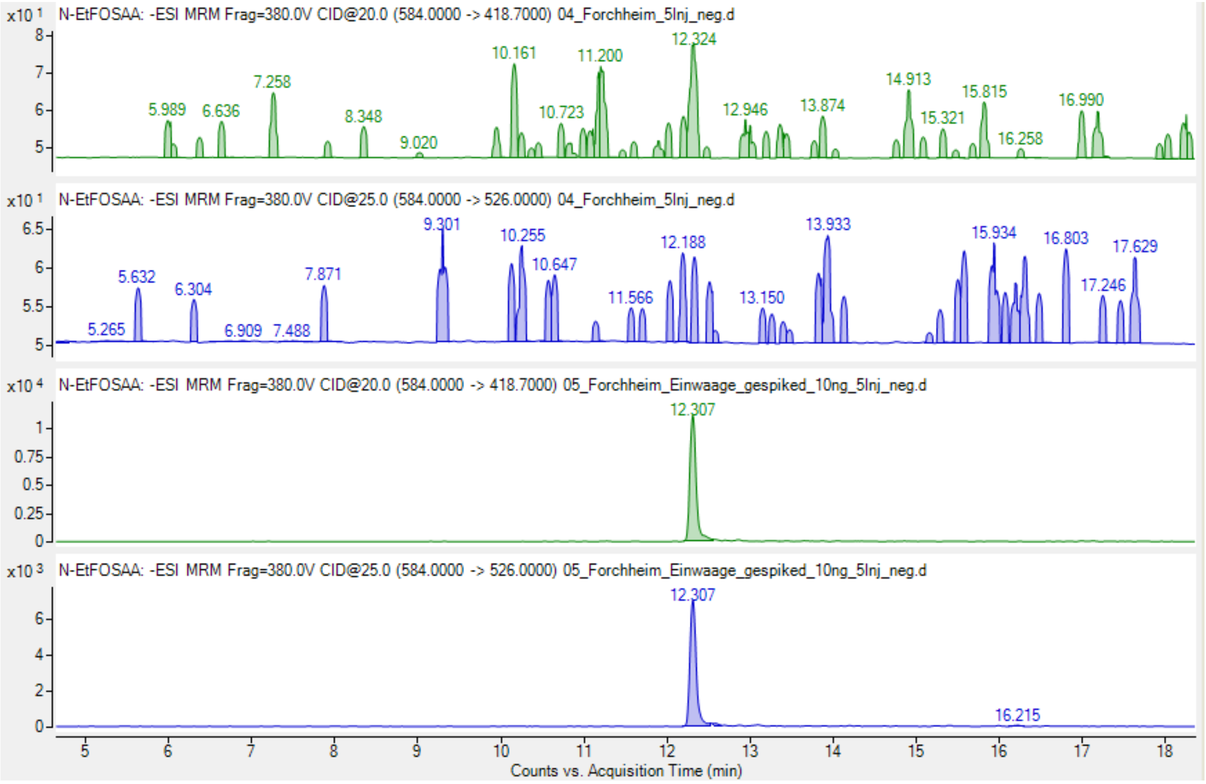

Fig. S1.44 EtFOSAA (584 -> 418.7, green; 584 -> 526, blue). Top two chromatograms: uncontaminated soil sample, bottom 2 chromatograms: spiked uncontaminated soil sample

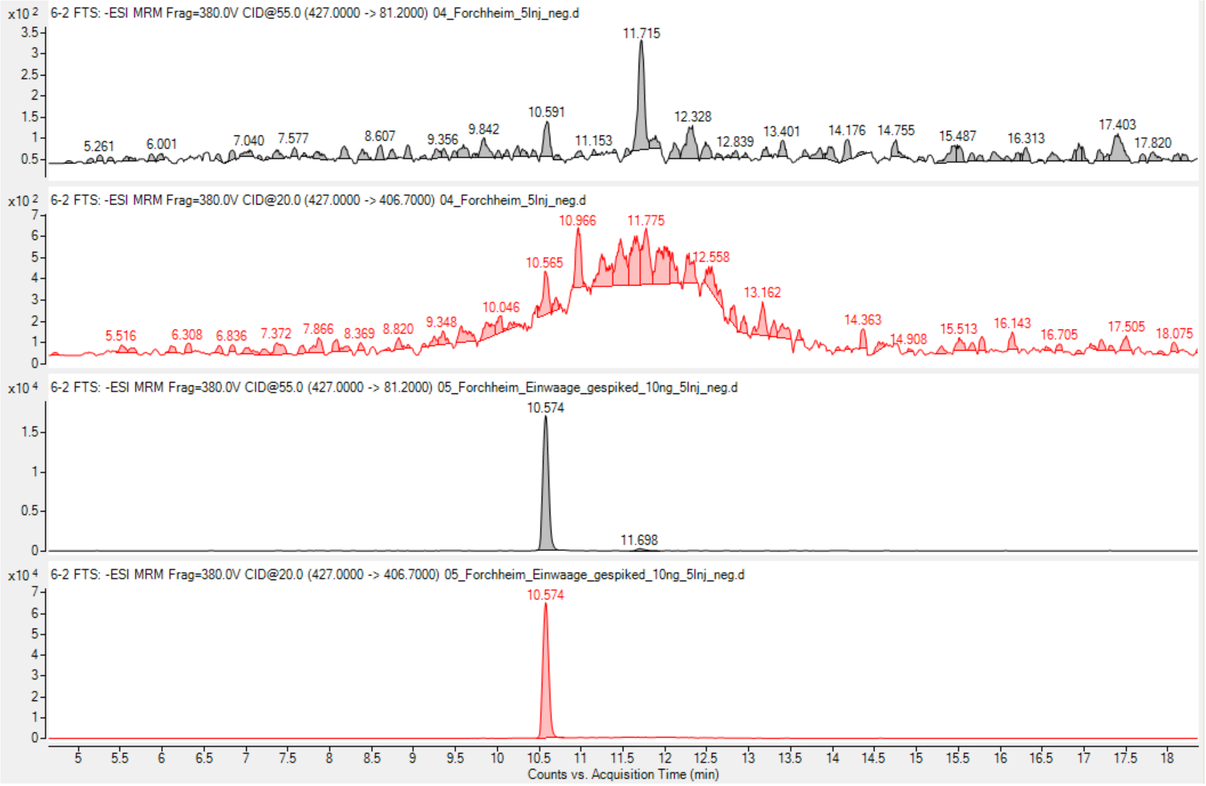

Fig. S1.45 6:2 FTS (427 -> 81.2, black; 427 -> 406.7, red). Top two chromatograms: uncontaminated soil sample, bottom 2 chromatograms: spiked uncontaminated soil sample

Confirmation of the identification with an authentic standard for samples 2, 3, and 4

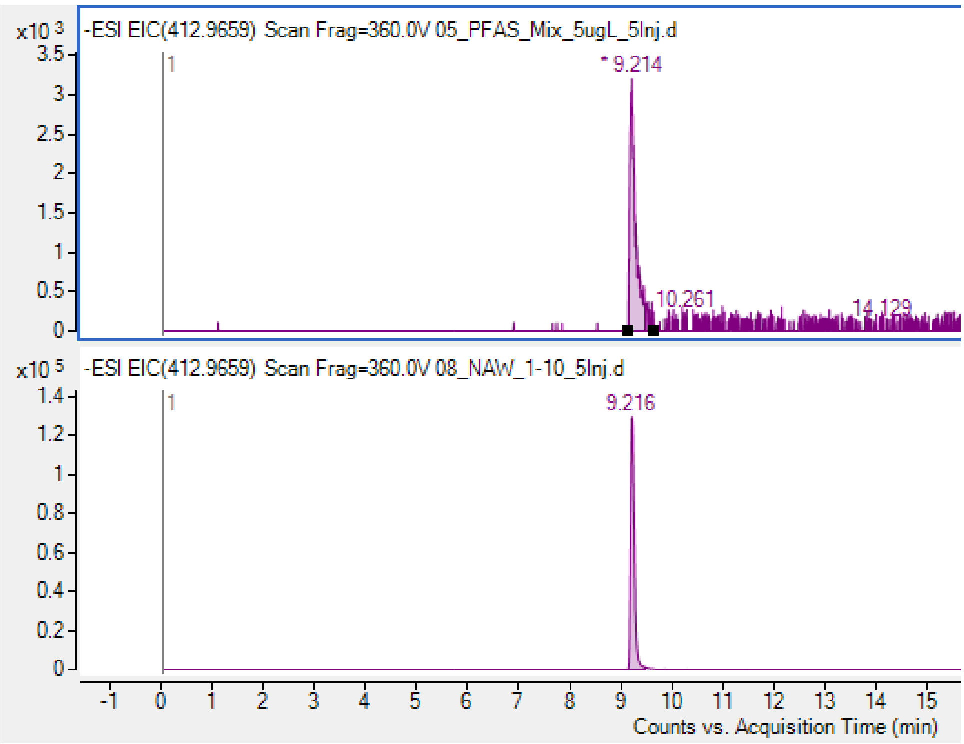

Fig. S1.46 Top: EIC of PFOA standard 5µg/L, bottom: 1/10 dilution of sample 2

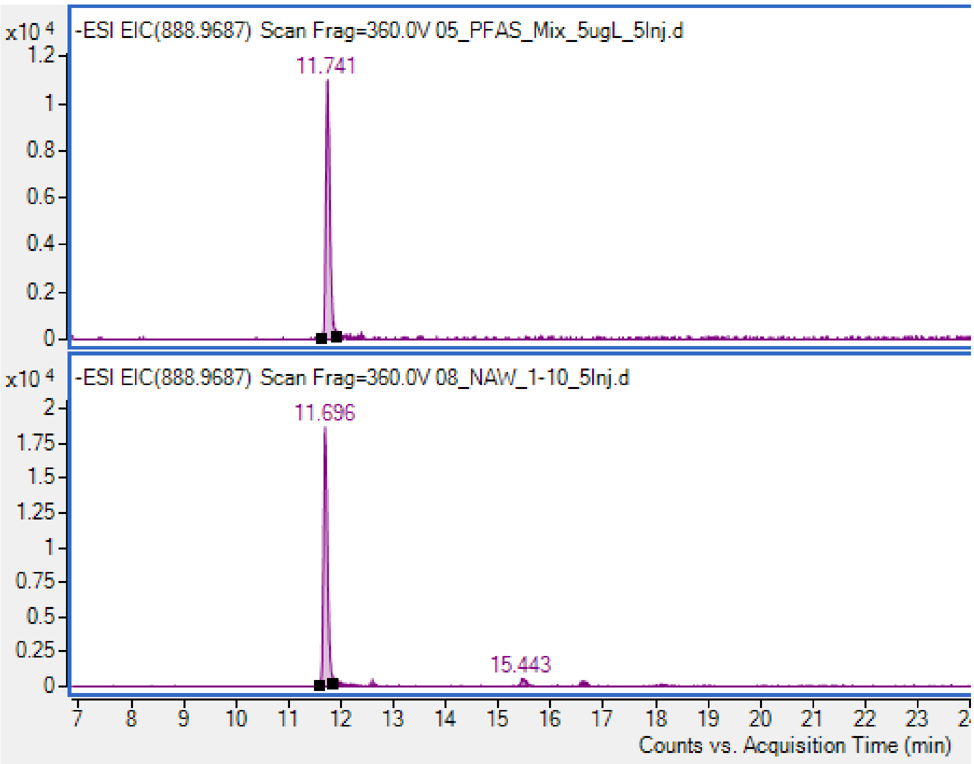

Fig. S1.47 Top: EIC of 6:2/8:2 diPAP standard 5µg/L, bottom: 1/10 dilution of sample 2

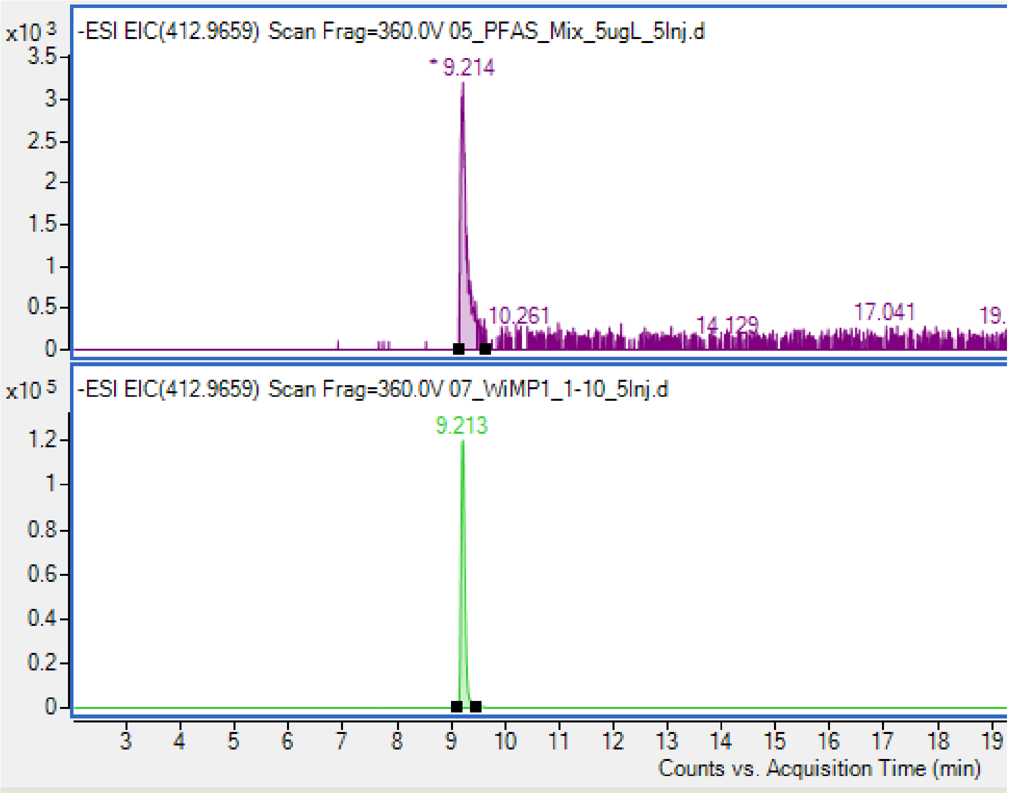

Fig. S1.48 EIC of PFOA standard 5 µg/L, bottom: 1/10 dilution of sample 3

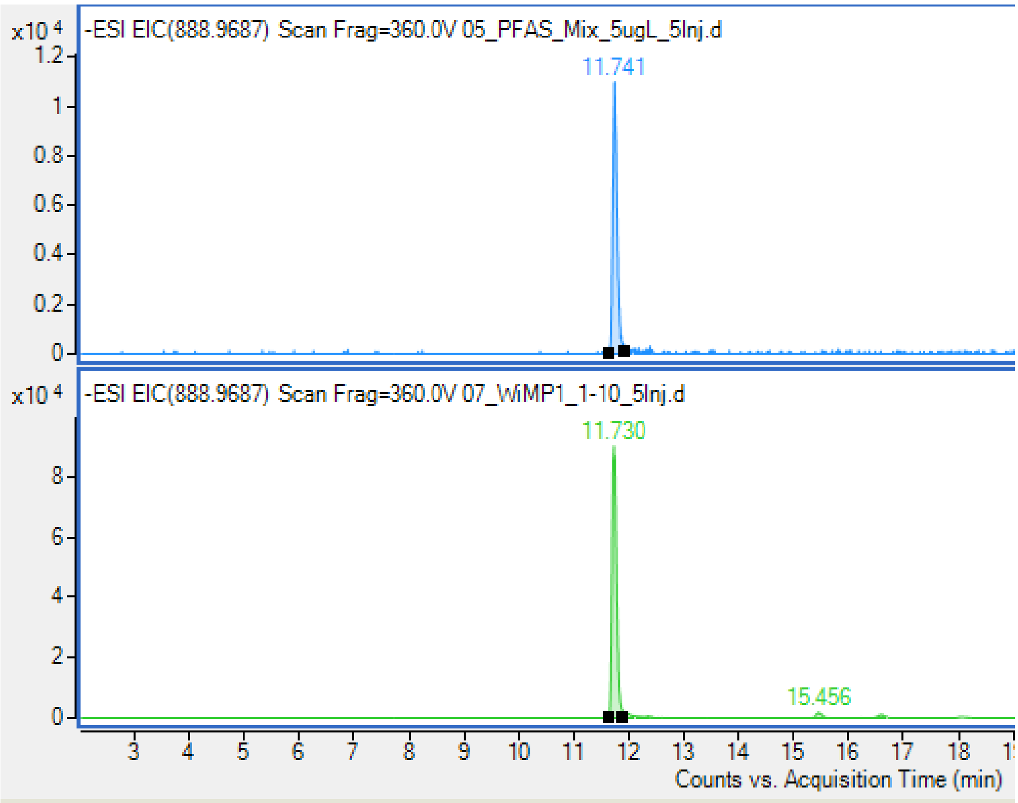

Fig. S1.49 EIC of 6:2/8:2 diPAP standard 5 µg/L, bottom: 1/10 dilution of sample 3

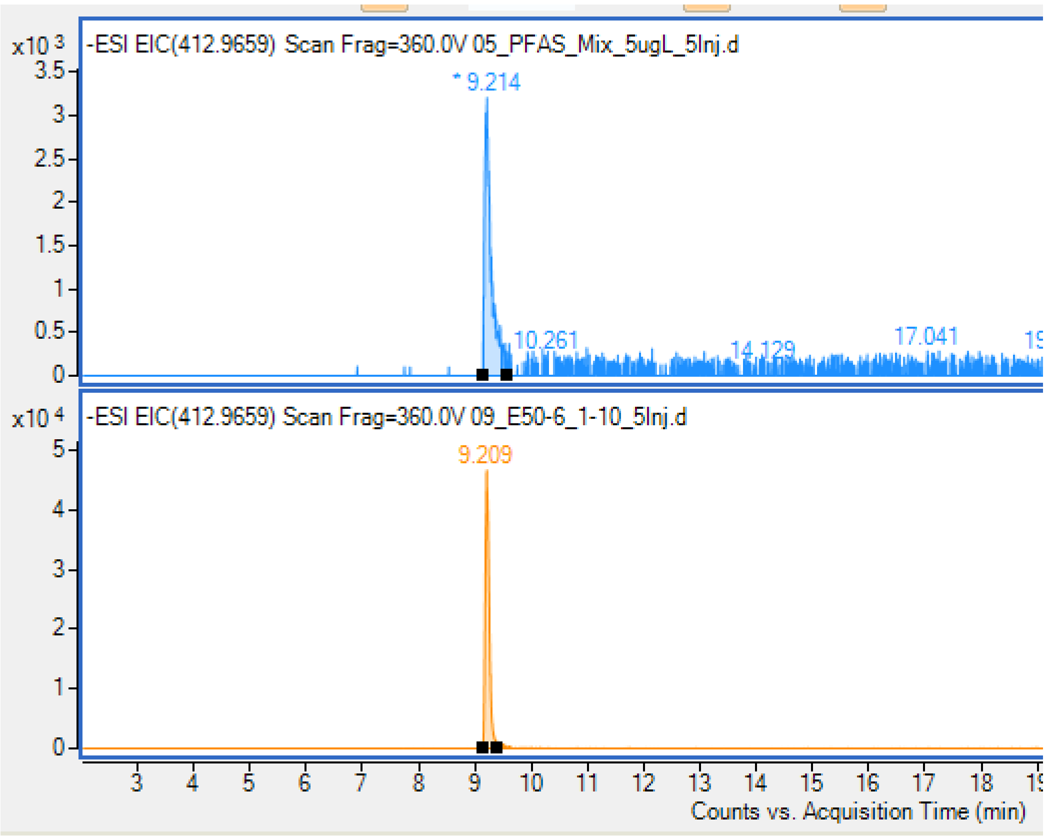

Fig. S1.50 Top: EIC of PFOA standard 5 µg/L, bottom: 1/10 dilution of sample 4

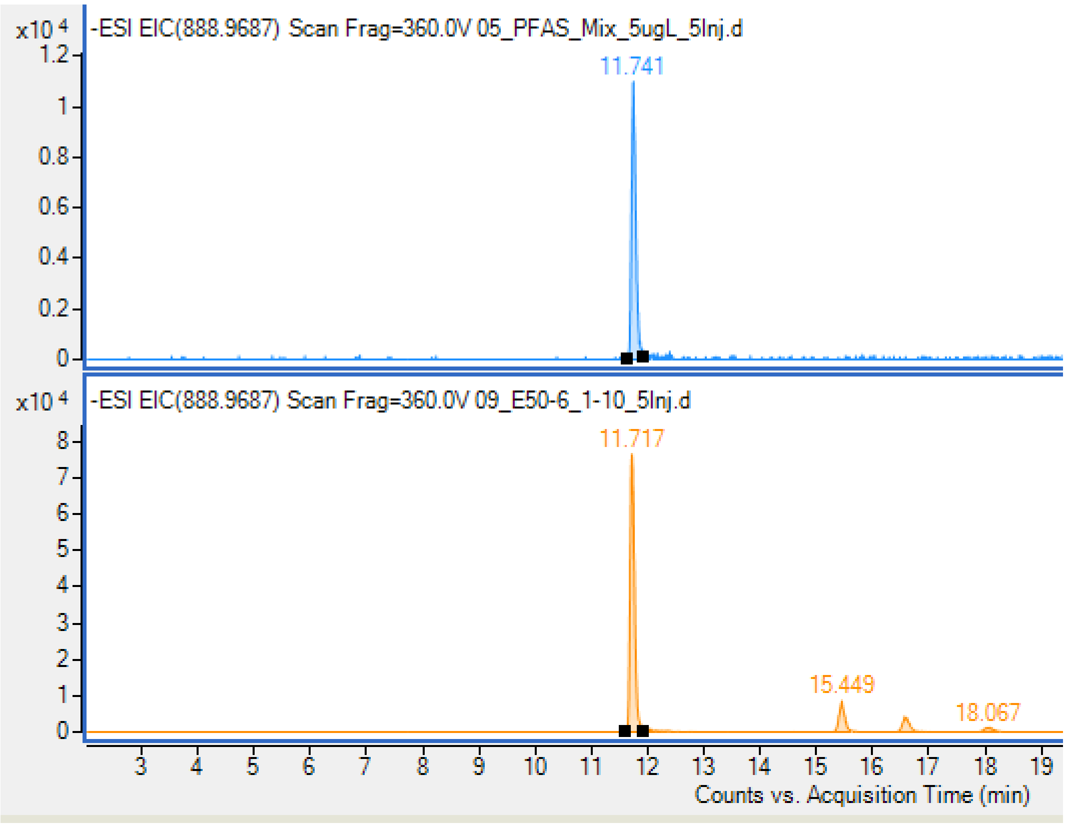

Fig. S1.51 Top: EIC of 6:2/8:2 diPAP standard 5 µg/L, bottom: 1/10 dilution of sample 4
